# A novel Z-number based multi-stage assessment framework for problem-based learning in practical courses

**DOI:** 10.1371/journal.pone.0349114

**Published:** 2026-05-18

**Authors:** Limin Yu, Zhe Chen, Zeyu Qin

**Affiliations:** Shandong Jiaotong University, Jinan, China; King Abdulaziz University Faculty of Medicine, SAUDI ARABIA

## Abstract

Confronting the growing disparity between standardized evaluation systems and personalized competency development in practical education, this study proposes a novel data-driven framework integrating Multi-Criteria Group Decision Making (MCGDM) to enhance curriculum assessment within Problem-Based Learning (PBL) environments. Specifically, the framework incorporates Z-number theory to effectively capture the uncertainty and reliability inherent in expert evaluations, addressing the challenges posed by subjective and imprecise human judgments. The well-established Multi-Attributive Border Approximation Area Comparison (MABAC) method is extended through the integration of Z-number modeling to enhance robustness in ranking and decision-making processes. A multi-stage assessment process is designed, encompassing a Pass check phase, a Score determination phase, and a Grading phase, aligned with the pedagogical principles of PBL. Furthermore, a hybrid Entropy-Criteria Importance Through Intercriteria Correlation (CRITIC) weighting scheme under Z-number representation is introduced to objectively determine the importance of evaluation criteria, considering both information dispersion and inter-criteria correlation. The proposed method is applied to a real-life case study involving 24 students, evaluated by multiple stakeholder groups, including peer teams, instructors, and industry experts. Sensitivity and comparative analyses suggest that the proposed framework provides a robust and reliability-aware assessment procedure within the studied course context. The findings indicate its potential to support a more transparent and structured evaluation process, although broader generalizability requires further validation across cohorts, courses, and institutions.

## 1 Introduction

Problem-Based Learning (PBL), originally developed in the 1960s to reform medical education, has since evolved into a transformative pedagogical approach across a wide range of disciplines, including engineering [1]. Its focus on inquiry, collaboration, and problem-solving aligns with modern engineering education and the UN Sustainable Development Goals (SDGs) [2]. In recent years, PBL has been increasingly integrated into engineering curricula to foster interdisciplinary competencies, technological innovation, and systems thinking. Applications range from AI-based waste management systems and wireless sensor networks in smart agriculture to virtual reality simulations for green manufacturing processes. These implementations reflect PBL’s growing relevance as a means of equipping students with critical thinking, technical fluency, and collaborative skills essential for tackling complex, real-world engineering problems. The following literature review further explores the theoretical foundations, implementation strategies, and educational outcomes of PBL within engineering disciplines.

With support from the SDGs, the integration of emerging technologies into engineering education has advanced interdisciplinary competency development through PBL. For example, in an AI course by Vargas, students worked on a smart waste-sorting system project, optimizing Convolutional Neural Network (CNN) algorithms and addressing data bias, which improved their AI ethics awareness by 25% [3]. Similarly, Wang and Cui’s Wireless Sensor Network (WSN) project in agricultural engineering enhanced students’ ability to integrate hardware design with cloud-based data management [4]. In green manufacturing, Chiou’s team used a Virtual Reality (VR) platform to optimize a solar panel production line, fostering a systems-level engineering mindset [5].

Research in educational psychology confirms that structured collaboration is key to PBL’s success, grounded in Vygotsky’s sociocultural theory and Chickering and Gamson’s work on best practices in education [6]. Teamwork is essential in PBL, as students tackle complex problems requiring diverse perspectives and collective reasoning. This collaboration enhances critical thinking and mirrors real-world professional contexts, where problem-solving is collaborative. Studies show that peer interaction in PBL increases motivation, accountability, and knowledge retention, making teamwork not just a necessity but a powerful pedagogical tool for academic and professional growth.

**Fig 1:**
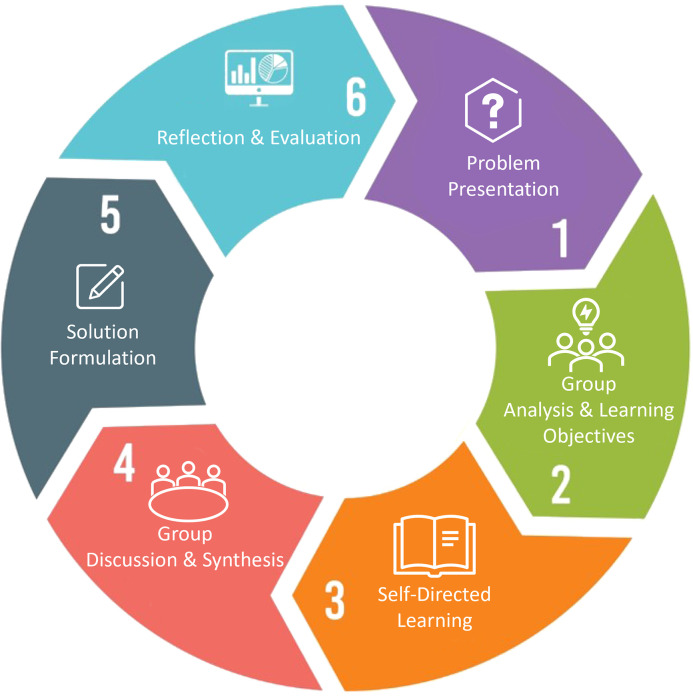
The PBL process.

While PBL encourages students to learn and solve problems collaboratively, its workflow can be characterized as a constructive, self-directed, collaborative, and contextual process [7]. Within this framework, each student is expected to engage in independent learning. The typical workflow of PBL is illustrated in Fig 1 and comprises six main stages. Before the process begins, students are organized into small groups. In the problem presentation stage, students are introduced to a complex, ill-structured, and open-ended problem. They then proceed to clarify the problem, identifying its inputs, expected outputs, the necessary knowledge to acquire, and formulating initial hypotheses. During the self-directed learning stage, each student or subgroup conducts independent research aligned with the established learning objectives. Subsequently, students reconvene to share their findings; the group engages in critical discussions to synthesize the newly acquired knowledge, revise initial hypotheses, and deepen their problem analysis through collaborative reasoning. Based on these discussions and the accumulated knowledge, students develop a solution, explanation, or response to the problem, which may take the form of a report, presentation, model, or other deliverable. Finally, students typically engage in reflective activities focused on the knowledge acquired, the learning methods employed, group dynamics, and the strengths and limitations of their proposed solutions. Self-assessment and peer assessment are often incorporated into this final stage to reinforce critical reflection and continuous improvement.

As the final stage of the PBL pedagogical approach, evaluation serves not only as a mechanism for feedback and culmination but, more importantly, as a critical tool for fostering deep thinking and metacognitive reflection in students [8,9]. Given PBL’s emphasis on self-directed inquiry and collaborative learning, traditional knowledge-recall assessments are insufficient for comprehensively measuring learning outcomes. Instead, a well-designed evaluation system should emphasize students’ holistic performance throughout the problem-solving process, encompassing analytical thinking, self-regulated learning, teamwork proficiency, and the application of integrated knowledge [10]. By combining formative and summative assessment strategies, educators can evaluate learners’ progress more comprehensively while providing timely, constructive feedback. Simultaneously, students gain clearer insights into their strengths and weaknesses through reflective practices, thus promoting continuous learning improvement. Moreover, assessment functions as a motivational catalyst, enhancing students’ engagement with learning objectives and strengthening their academic commitment. In this regard, assessment in PBL transcends its traditional evaluative role to become a pivotal driver of deeper learning engagement and comprehensive competency development [11].

Despite the widespread recognition of assessment’s pivotal role in fostering deep learning within PBL environments [12], several critical gaps remain in current evaluation practices:

- The multi-source assessment paradigm, involving instructors, tutors, industry experts, and peer evaluators, lacks robust mechanisms to reconcile heterogeneous data types and varying authority weights among stakeholders, often resulting in oversimplified averaging approaches that dilute domain-specific expertise. Moreover, the varying reliability of different evaluators is seldom accounted for, leading to potential biases and inconsistent aggregation of assessment results [4].
- While team collaboration constitutes a cornerstone of PBL pedagogy, existing evaluation systems fail to adequately decouple individual contributions from group outcomes, perpetuating measurement uncertainties caused by social loafing artifacts and cognitive biases such as halo effects and anchoring biases [13,14].
- Prevailing assessment frameworks prioritize static competency snapshots over dynamic capability trajectories, offering limited insights into learners’ metacognitive evolution across iterative problem-solving phases [12].

Although prior studies have advocated mixed-method assessments, few provide operationalizable models addressing these interconnected challenges through mathematically rigorous yet pedagogically meaningful integrations. This gap underscores the urgent need for an adaptive evaluation architecture that systematically harmonizes multi-stakeholder perspectives, quantifies latent competencies, and mitigates subjectivity risks, an imperative this study addresses through its novel MCGDM-driven assessment framework.

The remainder of this paper is structured as follows. Section 2 provides a detailed review of the theoretical foundations underpinning this study, including PBL in engineering education, MCGDM in course evaluation, Z-number theory, and the Multi-Attributive Border Approximation Area Comparison (MABAC) method. Section 3 introduces the key definitions and the proposed grading framework based on Z-number-enhanced MABAC (Z-MABAC) method, consisting of the pass check phase, score determination phase, and grading phase, in alignment with the evaluation process of PBL. Section 4 presents the implementation of the proposed framework through a real-world case study conducted in an educational context, illustrating the step-by-step application of the proposed method. Section 5 provides a comparative analysis with other established Z-number-based methods, validating the robustness and effectiveness of the proposed approach. Finally, Section 6 summarizes the major findings, practical implications, and future research directions of this work.

## 2 Literature review

This section is divided into 4 parts. Section 2.1 reviews the PBL in engineering education, Section 2.2 shows the application of MCGDM in course evaluation, Section 2.3 introduces the concept and developments of Z-numbers, Section 2.4 presents the MABAC method, and their integration in addressing uncertainty and reliability in decision-making contexts. A summary of the key literature reviewed is presented in Table 1.

**Table 1:** Summary of key literature in PBL, MCGDM, Z-numbers, and MABAC methods.

Author	Focus	Findings	Gaps	Contribution
[15] Nnamdi et al. (2025)	Problem-based learning in biomedical engineering in the era of generative AI	Problem-based learning in biomedical engineering in the era of generative AI	Problem-based learning in biomedical engineering in the era of generative AI	Problem-based learning in biomedical engineering in the era of generative AI
[16] Mahtani et al. (2024)	Project-based learning innovation in electro-mechanical engineering education	Project-based learning innovation in electro-mechanical engineering education	Project-based learning innovation in electro-mechanical engineering education	Project-based learning innovation in electro-mechanical engineering education
[17] Rodriguez-Sanchez et al.	Combined problem- and project-based learning in industrial engineering	Combined problem- and project-based learning in industrial engineering	Combined problem- and project-based learning in industrial engineering	Combined problem- and project-based learning in industrial engineering
[18] Ariza (2022)	PBL with in-home laboratories during COVID-19	PBL with in-home laboratories during COVID-19	PBL with in-home laboratories during COVID-19	PBL with in-home laboratories during COVID-19
[19] Alsamaray (2017)	AHP-based evaluation of student team-member contributions in cooperative learning	AHP-based evaluation of student team-member contributions in cooperative learning	AHP-based evaluation of student team-member contributions in cooperative learning	AHP-based evaluation of student team-member contributions in cooperative learning
[20] Alcázar-Ortega et al. (2023)	Multi-criteria student evaluation in university education	Multi-criteria student evaluation in university education	Multi-criteria student evaluation in university education	Multi-criteria student evaluation in university education
[21] Haktanır & Kahraman (2025)	Intuitionistic Z-AHP and Z-TOPSIS for hydrogen storage technology selection	Intuitionistic Z-AHP and Z-TOPSIS for hydrogen storage technology selection	Intuitionistic Z-AHP and Z-TOPSIS for hydrogen storage technology selection	Intuitionistic Z-AHP and Z-TOPSIS for hydrogen storage technology selection
[22] Capuano et al. (2020)	Fuzzy multi-criteria group decision making for peer assessment	Fuzzy multi-criteria group decision making for peer assessment	Fuzzy multi-criteria group decision making for peer assessment	Fuzzy multi-criteria group decision making for peer assessment
[23] Alam et al. (2023)	Intuitionistic Z-numbers for supplier selection under uncertainty	Intuitionistic Z-numbers for supplier selection under uncertainty	Intuitionistic Z-numbers for supplier selection under uncertainty	Intuitionistic Z-numbers for supplier selection under uncertainty
[24] Tan et al. (2024)	Z-number-enhanced MABAC for product design concept evaluation	Z-number-enhanced MABAC for product design concept evaluation	Z-number-enhanced MABAC for product design concept evaluation	Z-number-enhanced MABAC for product design concept evaluation

### 2.1 PBL in engineering education

Problem-Based Learning (PBL) has gained significant momentum in engineering education in recent years, driven largely by the demands of Industry 4.0 and the increasing emphasis on interdisciplinary, experiential learning. In biomedical engineering, a longitudinal three-year study conducted at Georgia Tech and Emory University implemented an advanced PBL framework tailored for AI applications in biomedicine. The program involved 92 undergraduate and 156 graduate students, resulting in notable outcomes including 16 student publications and the development of computational methods addressing real-world biomedical problems [15]. Similarly, in electromechanical engineering, research introduced a PBL model centered on real-world problem-solving and critical thinking. Student feedback highlighted high levels of satisfaction, especially in areas such as problem analysis, solution design, and the integration of theoretical knowledge with industrial practice [16]. Industrial engineering programs have also embraced PBL methodologies; for instance, the “Insights 4.0” initiative combined PBL with project-based learning, leading to increased student engagement and improved academic performance. Participants reported higher final grades alongside enhanced collaboration skills and both technical and soft competencies, underscoring PBL’s effectiveness in this domain [17]. Moreover, during the COVID-19 pandemic, engineering programs adapted by integrating at-home laboratories with PBL approaches to maintain educational quality [18]. This strategy not only preserved academic performance but also elevated student motivation and self-efficacy, demonstrating PBL’s efficacy in remote and hybrid learning environments.

Collectively, these examples underscore PBL’s versatility and profound impact within engineering education, equipping students with critical thinking, practical problem-solving abilities, and the adaptability essential for navigating today’s complex, interdisciplinary challenges. As educational demands evolve, PBL continues to stand out as a robust framework for preparing future-ready engineers.

### 2.2 MCGDM in course evaluation

MCGDM has proven effective in addressing the complexity inherent in student course evaluation. Traditional evaluation methods often rely on single or limited criteria, which fail to capture the multidimensional nature of learning outcomes. In contrast, MCGDM provides a structured framework that integrates perspectives from multiple stakeholders, such as students, instructors, and educational experts, thereby enabling a more comprehensive and equitable assessment. For example, an empirical study at Gulf University employed the Analytic Hierarchy Process (AHP) to evaluate student contributions in cooperative learning, successfully resolving conflicts in subjective assessments and facilitating more accurate credit distribution [19]. Additionally, MCGDM methods have been applied to optimize teaching plans and grading processes in university courses, as demonstrated in studies on energy market education and chemistry performance evaluations [20].

A key strength of MCGDM lies in its capacity to simultaneously consider a broad range of criteria without reducing evaluation outcomes to oversimplified metrics, including cognitive skills, creativity, teamwork, and presentation quality. Techniques such as AHP, Technique for Order Preference by Similarity to Ideal Solution (TOPSIS), and Simple Additive Weighting (SAW) enable the structured weighting and ranking of these diverse factors.

This approach is exemplified by Kara and Yıldırım’s study [25], which employed AHP and TOPSIS to assess high school chemistry students’ performance tasks, ensuring balanced consideration of psychomotor, cognitive, and affective domains. This approach is further illustrated by Yıldırım et al. [21], who developed an integrated intuitionistic Z-AHP and Z-TOPSIS methodology for hydrogen storage technology selection, explicitly incorporating both judgment reliability and decision-makers’ hesitancy under intuitionistic fuzzy environments. Moreover, MCGDM frameworks have demonstrated enhancements in fairness and transparency, particularly within group-based learning environments and interdisciplinary evaluations [26,27]. These methods also provide systematic alignment between teaching outcomes and program objectives, a critical consideration in outcome-based education models [28].

To further address the fuzziness and subjectivity inherent in human evaluations, researchers have integrated fuzzy logic, hesitant fuzzy sets, and probabilistic reasoning into MCGDM models. For instance, the Fuzzy Ordered Priority Approach for Multi-Criteria decision-making (FOPA-MC) model combines fuzzy logic with group decision-making for peer evaluation, effectively capturing the vagueness of subjective inputs [22]. Similarly, probabilistic hesitant fuzzy TODIM (an acronym in Portuguese for Interactive and Multi-Criteria Decision Making) and Evaluation based on Distance from Average Solution (EDAS) methods have been applied in evaluating Chinese language instruction quality, yielding promising results in mitigating evaluator hesitation and ambiguity [29].

Despite these advancements, several challenges remain. Fuzzy MCGDM models often involve high computational complexity and can be sensitive to initial assumptions such as criteria weights or decision-maker preferences. Furthermore, the need for adequate training of evaluators to use these advanced tools poses a risk to the consistency and reliability of assessment outcomes. A recent systematic review highlights the necessity for standardized frameworks and broader empirical validation across diverse disciplines [30].

In response to these challenges, researchers are exploring more robust tools for managing uncertain information. Among these, Z-number theory stands out for its ability to handle incomplete data while explicitly characterizing the reliability of information. Its unique capacity to fuse fuzzy and probabilistic information offers a flexible and accurate framework for modeling uncertainty, thereby more faithfully reflecting the complexity of real-world decision-making scenarios [23].

### 2.3 Z-number

In the realm of MCGDM, the complexity of decision structures and evaluation criteria often complicates the process, even for experts who are thoroughly familiar with the available alternatives. To address this challenge, the concept of the Z-number has been introduced to enhance the representation and measurement of the reliability associated with linguistic information. A Z-number is defined as Z=(A,B), where *A* represents the expert’s evaluation or preference, and *B* denotes the degree of reliability or possibility associated with *A*. This dual-dimensional structure provides a novel approach to modeling both uncertainty and reliability within decision-making contexts [23].

To promote the understanding and application of Z-numbers, extensive research has been conducted, leading to the development of various extended forms, such as the Z*-number, Z-advanced number, and Z + -number, which have been applied to a range of MCGDM problems [31]. However, due to the inherent complexity of directly manipulating and solving Z-numbers in practical applications, scholars have explored transforming Z-numbers into more tractable representations, such as Triangular Fuzzy Numbers (TFNs) and trapezoidal fuzzy numbers, thereby leveraging existing fuzzy set methodologies to solve decision-making problems more efficiently [24].

Kang delved into Z-number operations and established a transformation rule to convert Z-numbers into fuzzy numbers and trapezoidal fuzzy numbers [32]. Božanić transformed Z-numbers into TFNs and applied them in camp location selection by integrating level-based weight assessment and multi-attributive ideal-real comparative analysis [33]. Garg proposed a method for converting Z-numbers into granulated Z-numbers, further expanding the application scope of Z-numbers [34].

These studies indicate that converting Z-numbers into TFNs or trapezoidal fuzzy numbers has become a widely accepted and validated approach for facilitating their practical application in decision-making. This transformation not only simplifies the computational process but also ensures the effective utilization of Z-number theory in various contexts. While Z-numbers have demonstrated remarkable capabilities in addressing uncertainty and reliability, they still fall short in fully capturing the subjectivity of Decision-Makers (DMs) in certain scenarios, such as mortise and tenon structure selection. To comprehensively address the intertwined issues of subjectivity, uncertainty, and reliability, this study integrates rough numbers with Z-numbers.

### 2.4 Multi-attributive border approximation area comparison (MABAC) method

Since the MABAC method was introduced by Pamučar and Ćirović, it has been applied to address MAGDM problems, such as the selection of transport and processing resources at logistics centers [35]. Its efficiency was demonstrated by comparing it with the COPRAS, TOPSIS, MOORA, and VIKOR methods. Subsequently, Pamučar improved the original MABAC technique, enhancing its applicability in more complex decision-making scenarios [36]. Zhao integrated the notion of cumulative prospect theory into the original MABAC approach and developed the IF-MABAC method, which is known as the cumulative prospect theory IF-MABAC method [37]. Liu and Zhang proposed a MABAC method for dealing with complex and uncertain decision-making situations, which considers the distance between alternatives and the Border Approximation Area (BAA) [38]. Jana developed a MCGDM technique based on a traditional MABAC model with bipolar fuzzy numbers by introducing two aggregation operators [39].

**Fig 2:**
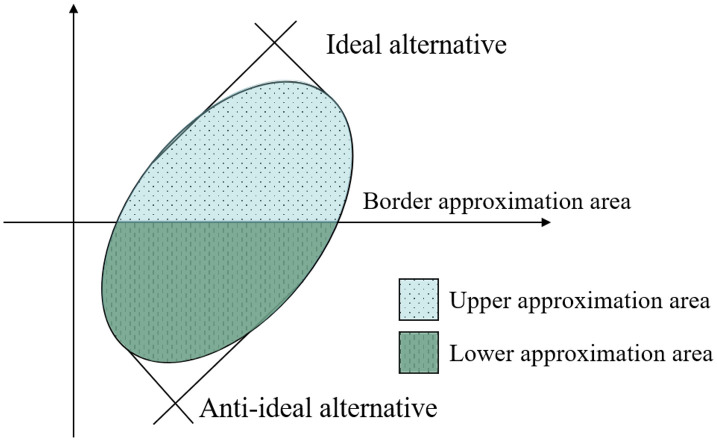
The upper and the lower approximation area.

In the MABAC method, for each criterion, the Border Approximation Area (BAA) is determined by calculating the geometric mean of the corresponding elements in the weighted decision matrix. Alternatives whose criterion values fall within the upper approximation area are considered close to the ideal solution for that specific criterion, whereas those located in the lower approximation area are regarded as closer to the anti-ideal solution, as illustrated in Fig 2. After calculating the distances from the BAA across all criteria, the arithmetic mean of these distances is computed for each alternative to derive its overall performance score. Finally, alternatives are ranked based on these aggregated values, with higher arithmetic means indicating superior performance.

By integrating Z-number theory into the MABAC method, this approach further strengthens the robustness of the evaluation process under conditions of uncertainty. The Z-number-based MABAC not only considers experts’ preference information but also explicitly incorporates the reliability of these judgments. This enhancement enables more transparent, interpretable, and reliable decision outcomes compared with conventional MABAC or other traditional evaluation methods [24].

## 3 Methodology

This study is a retrospective analysis of student assessment data collected in a PBL practical course delivered as part of the regular curriculum. The study protocol for the retrospective research use of these records was reviewed and approved by the ethics committee of the School of Art & Design, Shandong Jiaotong University (final approval dated 30 October 2025). Written informed consent was obtained from all participating students.

This study constitutes a retrospective analysis of student assessment data collected in a PBL practical course that was delivered as part of the regular curriculum. The corresponding author, who served as the course instructor, obtained permission from the relevant academic unit and administration before using these assessment records for research purposes, and written informed consent was obtained from all participating students. During normal course delivery and grade administration, the corresponding author necessarily had access to identifiable student information (including names and student ID numbers) as part of routine educational management, independent of the present research.

After the course had been completed and all grades had been finalised, and before any research analyses were initiated, the corresponding author exported the assessment records and anonymised them specifically for research use. In this anonymisation process, all direct identifiers were removed and replaced with randomly generated study codes; class and group labels were recoded, and any potentially identifying free-text information was removed or aggregated. The re-identification key linking study codes to individual students was stored securely by the corresponding author and was not shared with the other co-authors. Thus, for the purposes of this study, all co-authors only accessed a fully anonymised dataset and did not handle any identifiable information once the study formally started.

The study protocol, covering the retrospective use of these anonymised records, was reviewed and approved by the Ethics Committee of the relevant institution, which functions as an accredited Institutional Review Board (IRB). The consent form provided to students explained the study’s purpose, procedures, potential risks, and confidentiality safeguards. All procedures comply with the ethical standards of the Declaration of Helsinki and the Belmont Report.

In this PBL course, student performance is categorized into five distinct grades: A, B, C, D, and E, corresponding to score intervals of [90, 100], [80, 90), [70, 80), [60, 70), and below 60 (Fail), respectively. To develop a comprehensive evaluation system tailored for practice-oriented courses in application-focused universities, it is necessary to first define the overarching structure of the practical learning process. Considering that practical learning involves the development of multiple competencies, this study adopts a hierarchical analytical framework, which is commonly used to evaluate student performance across various stages and dimensions of learning in practice-based education.

**Fig 3:**
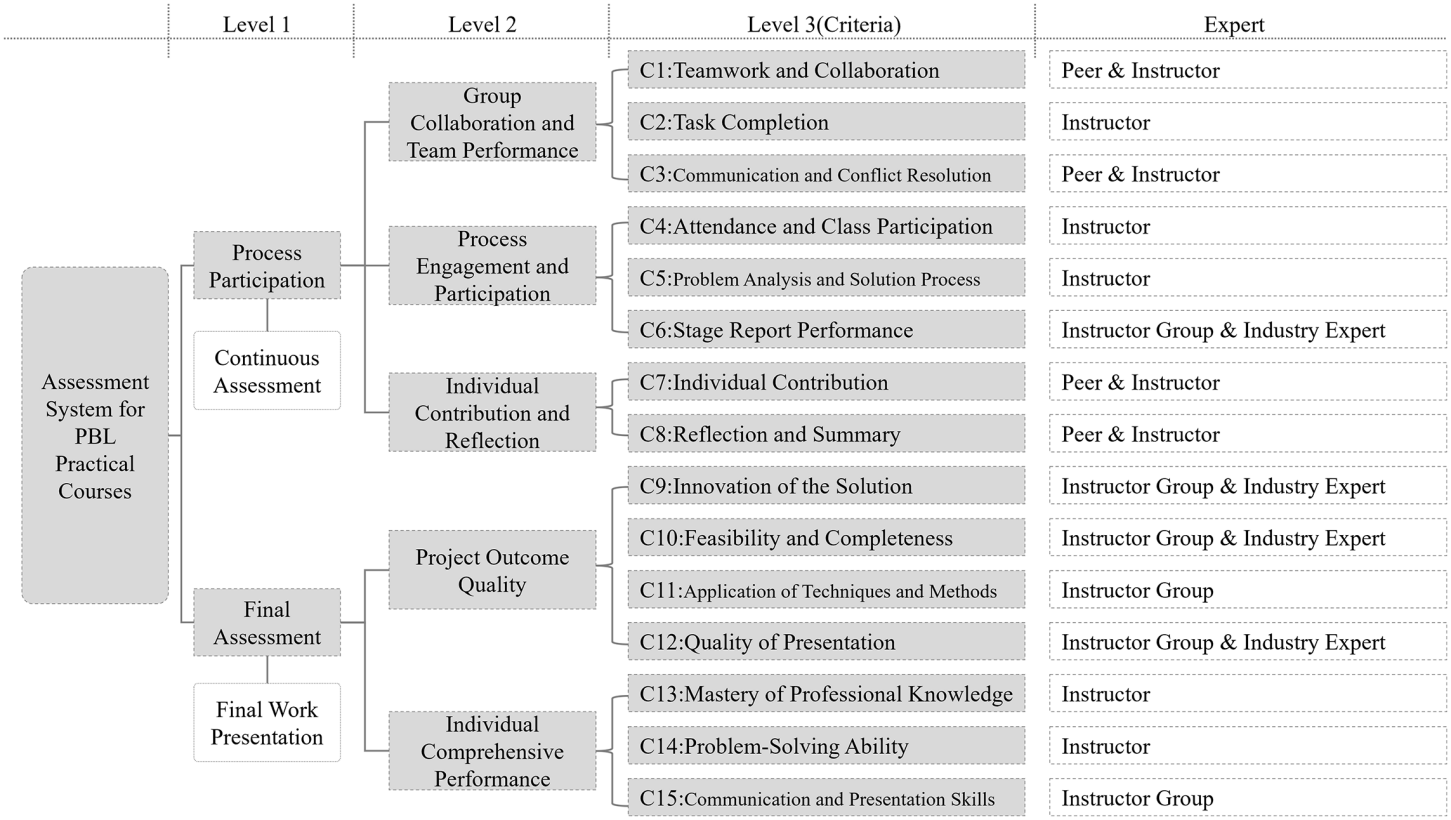
The multi-level analytic hierarchy framework.

As illustrated in Fig 3, the evaluation framework is constructed around two primary components: continuous assessment and final assessment. Continuous assessment prioritizes students’ ongoing engagement throughout the course, focusing on process-oriented behaviors such as teamwork, class participation, problem-solving, and individual reflection. In contrast, the final assessment emphasizes the quality of project deliverables and comprehensive individual performance at the course’s conclusion. After extensive literature review, expert consultation, and consideration of operational features of application-oriented institutions, a hierarchical assessment model was developed. At the top tier (Level 1), the model encapsulates overall course evaluation. This is decomposed at Level 2 into continuous and final assessment categories. Each category is further broken down into sub-categories and specific measurable indicators at Level 3, which constitute the criteria evaluated by experts. Each sub-category designates specific evaluators, including individual instructors, instructor teams, industry experts, and peer groups, to ensure balanced and multifaceted assessment perspectives.

However, it is important to acknowledge that despite the structured multi-criteria assessment framework and diversified evaluator composition, challenges persist in ensuring the reliability of subjective judgments, particularly those made by student peers. In practice-oriented courses that involve Peer Assessment (PA) as a component of continuous evaluation, students often lack sufficient domain expertise and evaluative consistency. Their assessments are prone to variability and uncertainty, influenced by factors such as limited judgmental maturity, interpersonal bias, or inconsistent interpretation of evaluation criteria. These limitations introduce epistemic and reliability-related uncertainties into the decision-making process.

In this PBL course, student performance is graded into five categories: A (90–100), B (80–90), C (70–80), D (60–70), and E (Fail). To develop a comprehensive evaluation system, the study adopts a hierarchical framework, often used in practice-based education. This framework consists of two main components: continuous assessment and final assessment. Continuous assessment focuses on ongoing student engagement, including teamwork, participation, problem-solving, and reflection, while final assessment evaluates the quality of the project and individual performance. After consulting literature and experts, a hierarchical assessment model was created, with top-level evaluations broken down into continuous and final assessments, each further divided into specific measurable criteria at Level 3. These criteria are assessed by various evaluators, such as instructors, industry experts, and peers, to ensure diverse perspectives, as shown in Fig 3.

Despite the structured framework, challenges remain in ensuring the reliability of subjective judgments, especially peer assessments, due to factors like lack of domain expertise, inconsistency in judgment, and bias, which introduce uncertainties into the evaluation process.

To strengthen the theoretical justification of the Level 3 criteria, Table 2 provides a literature support mapping for all fifteen criteria in Fig 3. Each criterion from C1 to C15 is linked to a representative study in educational assessment and PBL related research, ensuring that every criterion is grounded in established constructs and widely used evaluation dimensions in practice oriented courses. By organizing the criteria under the Level 2 dimensions and associating each criterion with distinct supporting evidence, Table 2 clarifies the rationale for selecting these criteria and improves the transparency and academic rigor of the assessment design.

**Table 2:** Summary of supporting literature for the 15 Level 3 assessment criteria.

Level 2 dimension	Criteria (Level 3)	Supporting reference for each criterion
Group Collaboration and Team Performance	C1	Schürmann et al. (2024) [40]
	C2	Zen et al. (2022) [41]
	C3	Thite et al. (2024) [42]
Process Engagement and Participation	C4	Adesina et al. (2023) [43]
	C5	Chen et al. (2024) [44]
	C6	Gomez-del Rio & Rodriguez. (2022) [45]
Individual Contribution and Reflection	C7	Katsenos & Pierrakeas. (2025) [46]
	C8	Rook et al. (2025) [47]
Project Outcome Quality	C9	Abdellatif & El-Wakeel. (2025) [48]
	C10	Yakob et al. (2023) [49]
	C11	Vargas et al. (2024) [50]
	C12	Di Palma et al. (2025) [51]
Individual Comprehensive Performance	C13	Syeed et al. (2022) [52]
	C14	Zhang et al. (2023) [53]
	C15	Taylor et al. (2024) [54]

**Fig 4:**
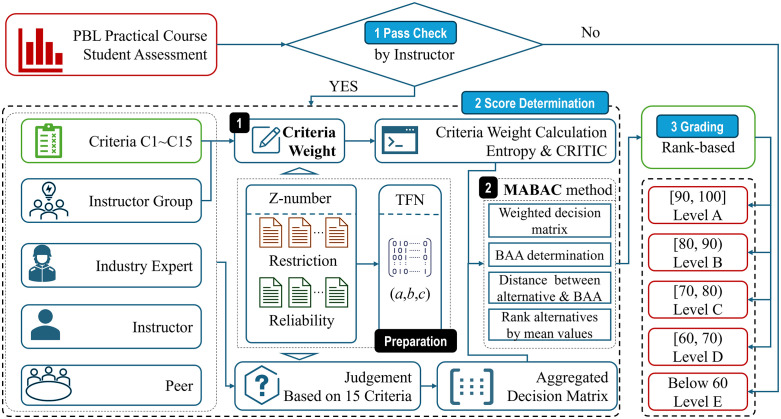
Framework of PBL practical course student assessment.

To address these concerns, this study integrates Z-number theory into the evaluation framework. Unlike traditional TFNs, Z-numbers incorporate a reliability measure alongside linguistic information, enabling the model to capture both the vagueness and the perceived trustworthiness of peer evaluations. By combining Z-number theory with the MABAC method, the proposed approach enhances the robustness of the evaluation under uncertainty. The methodological process, from Z-number construction to final ranking via MABAC, is illustrated in Fig 4. This procedure consists of three main phases: Pass Check, Score Determination, and Grading.

### 3.1 Pass check phase

The evaluation process begins with the Pass Check phase, during which instructors perform an initial qualitative screening of each student’s overall engagement and integrity throughout the course. This phase serves as a gatekeeping mechanism to ensure that only those who meet the fundamental requirements of participation are eligible for further assessment. The evaluation in this stage focuses on key behavioral indicators, including class attendance, task engagement, ethical conduct, and active involvement in team-based project activities.

Students who exhibit severe deficiencies are assigned a Fail status. These deficiencies may include chronic absenteeism, failure to complete assigned project tasks, or breaches of academic integrity. Examples of misconduct include plagiarism and other violations of ethical standards. These students are directly categorized into Grade E, which reflects a critically low level of engagement incompatible with the pedagogical goals of PBL courses. Notably, the assignment of an E grade is reserved for exceptional circumstances and is exceedingly rare in practice.

To uphold the fairness and credibility of the evaluation process, students who receive a Fail status are excluded from the subsequent performance ranking. This exclusion applies not only to their own assessments but also to the peer evaluations they provided. There are two main reasons for this. First, students who fail to meet basic participation standards likely have an incomplete or biased understanding of team dynamics and peer contributions. Second, including evaluations involving these students could introduce noise and distortion into the decision matrix, thereby compromising the consistency and reliability of the group decision-making results. Their removal ensures the integrity and robustness of the evaluation process.

Given the subjectivity and complexity inherent in evaluating performance within PBL-oriented curricula, students who pass this initial screening proceed to the Score Determination phase, where their performance is systematically assessed using the proposed Z-MABAC method.

### 3.2 Score determination phase

Following the preliminary filtering conducted in the Pass check phase, the evaluation process advances to the score determination phase. Given the diverse and PBL courses, a comprehensive evaluation must incorporate multiple perspectives and criteria. To this end, the evaluation framework includes 15 criteria spanning both continuous and final assessment components, each reflecting a specific dimension of student performance. These criteria are assessed by a heterogeneous group of evaluators, including the Instructor (main course teacher), an Instructor Group, an Industry Expert, and Peer groups composed of student teams.

**Fig 5:**
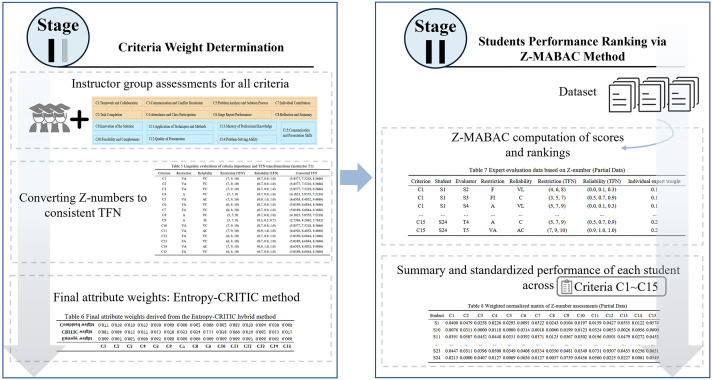
Workflow of stage 1 and stage 2.

To support a more structured, consistent, and reliability-aware evaluation of student performance in the studied PBL setting, the score determination phase adopts a structured two-stage decision-making framework based on Z-number theory and fuzzy MCGDM. The entire process begins with the transformation of linguistic evaluations into TFNs, followed by a systematic decomposition into two major stages. In stage 1, criteria weights are determined using instructor group evaluations. In stage 2, the Z-MABAC method is implemented in alternative ranking. This two-stage architecture is also illustrated in Fig 5. and described in detail below.

#### 3.2.1 Preparation: Z-number convert into TFNs.

Subjective assessments often involve uncertainty and inconsistent reliability. This issue is particularly evident in peer evaluations. To address this challenge, the present study adopts the Z-number formalism. In this formalism, each evaluation is expressed as a Z-number Z=(A,B).A denote the restriction of the variable, using a membership function $u_{A}:X\to \left[0,1\right]$ assigns a value in this interval to each element x∈X. Then we have $A=\left\{\left\langle x,u_{A}(x)\right\rangle |x\in [\text{0},\text{1}]\;\right\}$. B denote the reliability measure, similarly we have the membership function $B=\left\{\left\langle x,u_{B}(x)\right\rangle |x\in [\text{0},\text{1}]\;\right\}$.

Before proceeding with the evaluation stages, all qualitative assessments expressed as Z-numbers must be converted into a computationally tractable format. This applies to two sources of evaluation data. Instructor group’s Z-number assessments on criteria importance (used in Stage 1) and evaluators’ Z-number assessments on student performance across criteria (used in Stage 2).

As is shown in Table 3, a linguistic-to-fuzzy mapping table was established for both importance and reliability, ensuring consistent interpretation across expert groups. For instance, if an evaluator considers a criterion to be Very Important and expresses High Certainty about this judgment, it is represented as a Z-number with the restriction part A=(7,9,10) and the reliability part B=(0.7,0.8,1.0), corresponding respectively to the linguistic terms Very Important (FA) and Very Certain (VC).

**Table 3:** Z-number linguistic scale for criteria evaluation (important & reliable).

Symbol	Restriction TFN (A)	Interpretation (Importance / Evaluation)	Symbol	Reliability TFN (B)	Interpretation (Certainty)
VI	(1, 1, 3)	Very Inaccurate / Very Poor / Very Unimportant	VL	(0.0, 0.1, 0.3)	Very Low / Very Uncertain
I	(2, 4, 6)	Inaccurate / Poor / Unimportant	L	(0.1, 0.3, 0.5)	Low / Uncertain
FI	(3, 5, 7)	Fairly Inaccurate / Fair / Moderately Important	M	(0.3, 0.5, 0.7)	Medium / Moderately Certain
F	(4, 6, 8)	Fair / Acceptable / Moderately Accurate	C	(0.5, 0.7, 0.9)	Certain
A	(5, 7, 9)	Accurate / Good / Important	VC	(0.7, 0.9, 1.0)	Very Certain
FA	(6, 8, 10)	Fairly Accurate / Very Good / Very Important	AC	(0.9, 1.0, 1.0)	Almost Certain / Extremely Certain
VA	(7, 9, 10)	Very Accurate / Excellent / Very Important	–	–	–

The weight of each expert in the aggregation process is determined based on a two-level structure:

- For each criterion, the relative weights of contributing evaluator groups are defined in accordance with the course’s instructional design.
- Within each evaluator group, all individual members are assigned equal weight, reflecting a uniform contribution assumption.

For example, consider Criterion C1, which is jointly evaluated by peer students and the course instructor, each contributing 50% to the final score. In this context, peer evaluations are conducted within student teams, and each student is assessed only by their teammates, excluding self-assessment. Suppose a team consists of five members. For any given student in the team, the remaining four peers each contribute equally to the peer evaluation component. Therefore, each peer is assigned a weight of 0.125 (i.e., 0.5 ÷ 4), and the instructor holds a weight of 0.5 in the aggregation of this criterion.

After standardizing all evaluations into Z-number representations, the assessment proceeds using the Z-MABAC method. This method facilitates aggregation, normalization, and comparison of Z-valued assessments across criteria and evaluators, while preserving both performance ratings and their associated reliability.

To make the Z-number applicable for computational processing, each Z-number can be transformed into a single representative TFN. This is achieved by computing the expected value of the fuzzy set modified by the reliability function, as proposed by Zadeh. The $u_{A}\left(x\right)$ and $u_{B}\left(x\right)$ mentioned before can be converted to triangular membership functions, and defuzzified value *α* can be calculated by the following equation.


$$\begin{array}{c} \alpha =\frac{\int x\mu_{A}\left(x\right)dx}{\int \mu_{A}\left(x\right)dx} \end{array}$$ (1)

Where $u_{A}\left(x\right)$ is the membership function of the fuzzy restriction component, modulated by the reliability component. This transformation enables the integration of both subjective evaluations and their associated confidence levels into a unified TFN format suitable for further processing in MABAC.

Then we have


$$\begin{array}{c} Z=\left\{\left\langle x,\mu_{Z}(x)\right\rangle \;|\;\mu_{Z}(x)=\mu_{A}\left(\frac{x}{\sqrt{\alpha }}\right),\;x\in [\text{0},\text{1}]\right\} \end{array}$$ (2)

Z-number then can be converted into TFNs, shown as


$$\begin{array}{c} Z_{i}=\left(a_{i},b_{i},c_{i}\right) \end{array}$$ (3)

This transformation adjusts the evaluated importance or performance by accounting for the evaluator’s confidence, thereby ensuring that assessments with higher uncertainty exert proportionally less influence on the final decision. To facilitate clarity and reproducibility, all relevant symbols and definitions used throughout the following stages are summarized in Table 4.

**Table 4:** Notation and definitions.

Symbol	Description
*m*	The number of students included in the final ranking (excluding those assigned Grade E)
*n*	Number of criteria
*k*	Number of evaluators
*M*	The total number of students included in the final ranking (excluding those assigned Grade E)
${\tilde{Z}}_{ijk}=\left({\tilde{A}}_{ijk},{\tilde{R}}_{ijk}\right)$	Z-number given by expert kk for alternative ii on criterion *j*
${\tilde{A}}_{ijk}=\left(\overset{\text{1}}{\underset{ijk}{a}},\overset{\text{2}}{\underset{ijk}{a}},\overset{\text{3}}{\underset{ijk}{a}}\right)$	Restriction TFN of ${\tilde{Z}}_{ijk}$
${\tilde{R}}_{ijk}=\left(\overset{\text{1}}{\underset{ijk}{r}},\overset{\text{2}}{\underset{ijk}{r}},\overset{\text{3}}{\underset{ijk}{r}}\right)$	Reliability TFN of ${\tilde{Z}}_{ijk}$
$\begin{array}{c} \overset{\left(E\right)}{\underset{j}{w}} \end{array}$, $\text{\textbackslash{}hspace\{0.33em}\;\}\overset{\left(C\right)}{\underset{j}{w}}$, $w_{j}$	Weight of criterion *j*, determined by Entropy, CRITIC and hybrid Entropy-CRITIC method
*λ*	Balance coefficient between Entropy and CRITIC weights
$\omega_{k}$	Weight of expert *k*, assigned subjectively
$D_{ij}$	Final border proximity coefficient (MABAC score) of alternative *i* on criterion *j*
$\pi_{x}\;$	Proportion of students in grade *x*, where x∈{A,B,C,D} and $\sum \pi_{x}=1$

#### 3.2.2 Stage 1: Criteria weight determination.

This stage determines the objective weights of evaluation criteria based on instructor group assessments. To capture both the dispersion of criteria information and the mutual independence among criteria, a hybrid weighting method combining Entropy and CRITIC is adopted. In this hybrid scheme, the Entropy component reflects the degree of dispersion in the evaluation information, while the CRITIC method is used to determine the weight of each criterion based on contrast intensity and inter-criteria conflict. Specifically, the weight of each criterion is determined by calculating the contrast intensity between that criterion and all other criteria, considering how much the criterion contrasts with the other criteria in the decision matrix [55,56]. The detailed computational steps are presented as follows.

**Step 1:** Each instructor provides a Z-number evaluation of the importance of every criterion. After converting these Z-numbers to TFNs, they are aggregated to form a consensus fuzzy value for each criterion:


$$\begin{array}{c} {\overset{―}{Z}}_{j}=\frac{\text{1}}{K}\sum_{k=1}^{K}\overset{*}{\underset{jk}{\tilde{Z}}} \end{array}$$ (4)

where *K* is the number of experts in the instructor group.

**Step 2:** Each aggregated TFN ${\overset{―}{Z}}_{j}=\left(a_{j},b_{j},c_{j}\right)$ is normalized across all criteria:


$$\begin{array}{c} P_{ij}=\left(\frac{a_{ij}}{\sum_{i}a_{ij}},\text{\textbackslash{}hspace\{0.33em}\}\frac{b_{ij}}{\sum_{i}b_{ij}},\text{\textbackslash{}hspace\{0.33em}\}\frac{c_{ij}}{\sum_{i}c_{ij}}\right) \end{array}$$ (5)

**Step 3:** Compute the entropy of each criterion by the following equations.


$$\begin{array}{c} e_{j}=-\frac{\text{1}}{ln\left(m\right)}\sum_{i=1}^{m}P_{ij}lnP_{ij}\text{\textbackslash{}hspace\{0.33em}\} \end{array}$$ (6)

$$\begin{array}{c} \text{\textbackslash{}hspace\{0.33em}\;\}\overset{\left(E\right)}{\underset{j}{w}}=\frac{\left|1-e_{j}\right|}{\sum_{j=1}^{n}|1-e_{j}|} \end{array}$$ (7)

**Step 4:** Compute the standard deviation and inter-criteria correlation to derive CRITIC weights by the following equations.


$$\begin{array}{c} C_{j}=\sigma_{j}\sum_{k=1}^{n}(1-r_{jk})\text{\textbackslash{}hspace\{0.33em}\;\} \end{array}$$ (8)

$$\begin{array}{c} \overset{\left(C\right)}{\underset{j}{w}}=\frac{C_{j}}{\sum_{j=1}^{n}C_{j}} \end{array}$$ (9)

**Fig 6:**
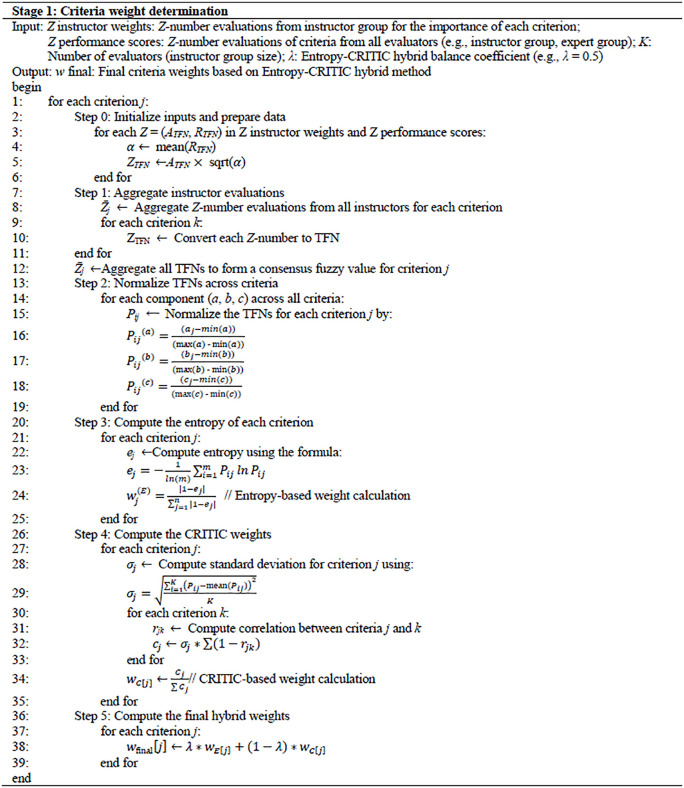
Pseudocode of Stage 1.

**Step 5:** Final hybrid weights are combined by entropy and CRITIC weights:


$$\begin{array}{c} w_{j}=\lambda \cdot \overset{\left(E\right)}{\underset{j}{w}}+(1-\lambda )\cdot \overset{\left(C\right)}{\underset{j}{w}} \end{array}$$

where λ∈[0,1] controls the fusion degree, λ close to 1 emphasizes the Entropy-based weights, while close to 0 emphasizes the CRITIC-based weights, and typically set λ=0.5. The pseudocode of this stage is shown in Fig 6.

#### 3.2.3 Stage 2: Students performance ranking via Z-MABAC method.

With the criteria weights determined, the Z-MABAC method is applied to rank student alternatives based on expert and peer evaluations. Each expert provides a Z-number ${\tilde{Z}}_{ijk}=\left({\tilde{A}}_{ijk},{\tilde{R}}_{ijk}\right)$. The Z-numbers are converted into TFNs according to Eqs. (1) to (3) at the preparation stage.

**Step 1:** Aggregate TFNs Across Experts. TFNs for each student *i* and criterion *j* using a weighted average:


$$\begin{array}{c} \overset{*}{\underset{ij}{\tilde{Z}}}=\sum_{k=1}^{K}\omega_{k}\cdot \overset{*}{\underset{ijk}{\tilde{Z}}} \end{array}$$ (10)

This yields a unified fuzzy decision matrix $\tilde{X}=[\overset{*}{\underset{ij}{\tilde{Z}}}{]}_{m\times n}$.

**Step 2:** Normalize each element $\overset{*}{\underset{ij}{\tilde{Z}}}=\left(a_{ij},b_{ij},c_{ij}\right)$ using fuzzy min–max normalization. For benefit criteria:


$$\begin{array}{c} {\tilde{N}}_{ij}=\left(\frac{a_{ij}-\overset{min}{\underset{j}{a}}}{\overset{max}{\underset{j}{a}}-\overset{min}{\underset{j}{a}}},\text{\textbackslash{}hspace\{0.33em}\}\frac{b_{ij}-\overset{min}{\underset{j}{b}}}{\overset{max}{\underset{j}{b}}-\overset{min}{\underset{j}{b}}},\text{\textbackslash{}hspace\{0.33em}\}\frac{c_{ij}-\overset{min}{\underset{j}{c}}}{\overset{max}{\underset{j}{c}}-\overset{min}{\underset{j}{c}}}\right) \end{array}$$ (11)

In this study, all 15 criteria are formulated as benefit-type, meaning that higher values indicate better performance. Therefore, normalization follows the benefit-type formula above for all criteria. Once for cost criteria, the numerator becomes $\overset{max}{\underset{j}{a}}-a_{ij}$.

**Step 3:** Multiply normalized TFNs with attribute weights $w_{j}$, derived from the Entropy-CRITIC hybrid method:


$$\begin{array}{c} {\tilde{V}}_{ij}=w_{j}\cdot {\tilde{N}}_{ij} \end{array}$$ (12)

**Step 4:** Compute the BAA for each criterion:


$$\begin{array}{c} {\tilde{G}}_{j}=\frac{\text{1}}{m}\sum_{i=1}^{m}{\tilde{V}}_{ij} \end{array}$$ (13)

**Step 5:** The final MABAC score is the distance from the BAA:


$$\begin{array}{c} D_{ij}=\text{Fuzzy Distance}\left({\tilde{V}}_{ij},\text{\textbackslash{}hspace\{0.33em}\}{\tilde{G}}_{j}\right) \end{array}$$ (14)

Which can be calculated using the vertex method:


$$\begin{array}{c} D_{ij}=\sqrt{\frac{\text{1}}{\text{3}}\left[(a_{\text{1}}-a_{\text{2}}{)}^{\text{2}}+(b_{\text{1}}-b_{\text{2}}{)}^{\text{2}}+(c_{\text{1}}-c_{\text{2}}{)}^{\text{2}}\right]} \end{array}$$ (15)

Here, $\left(a_{\text{1}},\;b_{\text{1}},\;c_{\text{1}}\right)$ and $\left(a_{\text{2}},\;b_{\text{2}},\;c_{\text{2}}\right)$ represent the TFNs of ${\tilde{V}}_{ij}$ and ${\tilde{G}}_{j}$ respectively.

**Step 6:** Final Score and Ranking. Aggregate the distances across all criteria for each alternative:


$$\begin{array}{c} S_{i}=\sum_{j=1}^{n}D_{ij} \end{array}$$ (16)

**Fig 7:**
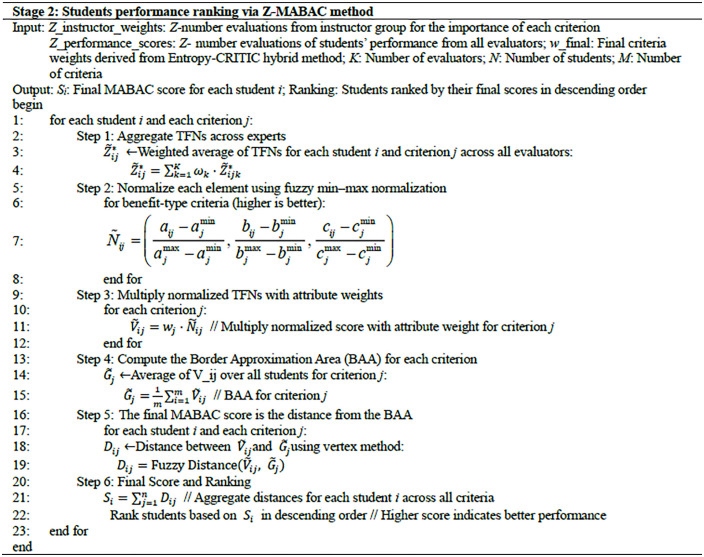
Pseudocode of stage 2.

Rank all students based on the descending order of $S_{i}$. A higher score indicates stronger performance relative to the group under the weighted, uncertainty-aware multi-criteria framework. The pseudocode of this stage is shown in Fig 7.

### 3.3 The grading phase

With the exclusion of students who failed to meet the basic participation or ethical standards during the Pass Check phase, the remaining student cohort proceeds to the final Grading process. These students have demonstrated adequate engagement and are deemed eligible for ranking and grade assignments. Based on institutional records and empirical observations from prior course implementations, the distribution of grades among this qualified population generally adheres to a predefined ratio.

Let $\pi =\left[\pi_{A},\pi_{B},\pi_{C},\pi_{D}\right]$ denote the proportion vector for Grades A through D, where x∈{A,B,C,D} and $\sum \pi_{x}=1$. In this study, the adopted distribution is π=[0.20,0.45,0.30,0.05]. Let *M* be the total number of students who passed the initial screening and are eligible for ranking. Students are sorted in descending order based on their final composite performance scores obtained through the Z-MABAC method. The grade assignment is conducted based on percentile thresholds derived from the distribution vector *π*, with ranking intervals determined as shown in Table 5. For example, assuming *M* = 24, the resulting grade intervals are as follows:

**Table 5:** Grade assignment intervals based on percentile thresholds.

Grade Level	Proportion (π)	Index Interval (Student Rank)	Proportion (case)	Index Interval (case)
A	$\pi_{A}$	$i\in \left[1,\text{\textbackslash{}hspace\{0.33em}\}\left\lfloor \pi_{A}\cdot M\right\rfloor \right]$	0.20	1-5
B	$\pi_{B}$	$i\in \left[\left\lfloor \pi_{A}\cdot M\right\rfloor +1,\text{\textbackslash{}hspace\{0.33em}\}\left\lfloor (\pi_{A}+\pi_{B})\cdot M\right\rfloor \right]$	0.45	6-16
C	$\pi_{C}$	$i\in \left[\left\lfloor (\pi_{A}+\pi_{B})\cdot M\right\rfloor +1,\text{\textbackslash{}hspace\{0.33em}\}\left\lfloor (\pi_{A}+\pi_{B}+\pi_{C})\cdot M\right\rfloor \right]$	0.30	17-23
D	$\pi_{D}$	$i\in \left[\left\lfloor (\pi_{A}+\pi_{B}+\pi_{C})\cdot M\right\rfloor +1,\text{\textbackslash{}hspace\{0.33em}\}M\right]$	0.05	24

- Grade A: Students ranked 1st to 5th
- Grade B: Students ranked 6th to 16th
- Grade C: Students ranked 17th to 23rd
- Grade D: Students ranked 24^th^

In order to emphasize the technical advantages of the Z-MABAC method compared to simpler evaluation approaches, Table 6 provides a comparative analysis across several key dimensions.

**Table 6:** Comparison of traditional and proposed assessment methods.

Assessment method	Evaluation dimensions	Flexibility	Evaluation type	Scalability	Diversity
Traditional	Single	Limited	Subjective	No	Low
Proposed method	Multi	Extensive	Objective	Yes	High

This grading mechanism ensures a consistent and fair mapping between performance rankings and grade levels, grounded in institutional experience.

## 4 Case study

### 4.1 Background description and the pass check phase

This study was conducted in the context of the course “Design investigation and analysis”, involving 24 students divided into four peer groups of six students each. In the present study, no student was disqualified during this phase. All 24 students demonstrated sufficient participation, attendance, and ethical behavior, and therefore passed the initial screening. As a result, the performance evaluation proceeded with the full cohort, without the need to assign Grade E.

**Fig 8:**
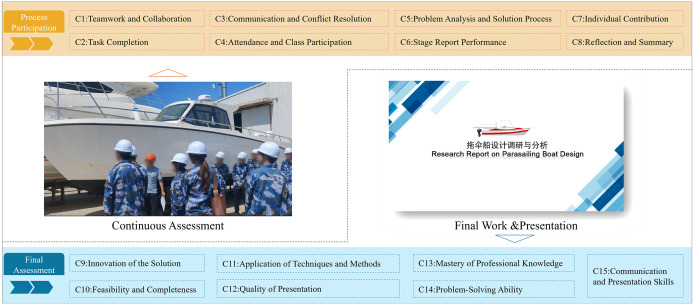
The assessment criteria in the course “Design investigation and analysis”.

Fig 8 shows the fifteen assessment criteria, which cover key dimensions of student learning outcomes and behaviors, including teamwork, task completion, communication, attendance, problem analysis, stage report quality, individual contributions, reflection, innovation, feasibility, technical application, presentation quality, professional knowledge mastery, problem-solving skills, and communication abilities.

The evaluation process incorporated a multi-expert structure to capture diverse perspectives: the peer groups, a core instructor team consisting of five members (T1 to T5), a primary instructor, and an industry expert. Each evaluator cluster was assigned specific roles in assessing fifteen criteria related to student performance, ensuring a comprehensive and balanced appraisal framework.

Expert weights were assigned based on the cluster roles, with peers and instructors sharing equal influence on C1, C3, C7, C8; while other criteria such as C6, C9 and C10 were evaluated solely by the instructor group or shared between the instructor team and industry expert. Criteria C6, C9, and C10 were weighed 60% by the instructor team and 40% by the industry expert, reflecting the relevance of practical industry insights. The related individual expert weights were then computed using the algorithmic approach, and the resulting values are presented in Table 7.

**Table 7:** Expert weight assignment.

No.	Expert Group 1	Percentage 1	Individuals/group	Individual expert weight
C1	Peer	0.5	5	0.1
	Instructor	0.5	1	0.5
C2	Instructor	1	1	1
C3	Peer	0.5	5	0.1
	Instructor	0.5	1	0.5
C4	Instructor	1	1	1
C5	Instructor	1	1	1
C6	Instructor Group	0.6	5	0.125
	Industry Expert	0.4	1	0.4
C7	Peer	0.5	5	0.1
	Instructor	0.5	1	0.5
C8	Peer	0.5	5	0.1
	Instructor	0.5	1	0.5
C9	Instructor Group	0.6	5	0.125
	Industry Expert	0.4	1	0.4
C10	Instructor Group	0.6	5	0.125
	Industry Expert	0.4	1	0.4
C11	Instructor Group	1	5	0.2
C12	Instructor Group	0.6	5	0.125
	Industry Expert	0.4	1	0.4
C13	Instructor	1	1	1
C14	Instructor	1	1	1
C15	Instructor Group	1	5	0.2

To address the inherent uncertainty and subjectivity in human evaluation, Z-numbers were employed to represent each expert’s judgment as a pair of fuzzy values: the restriction and reliability of the assessment. As shown in Table 4, linguistic terms for restriction ranged from “Very Inaccurate / Very Poor / Very Unimportant” to “Very Accurate / Excellent / Very Important,” modeled as TFNs, while reliability levels ranged from “Very Low / Very Uncertain” to “Almost Certain / Extremely Certain,” also expressed as TFNs.

### 4.2 Implementation of score determination and grading phases based on the proposed model

#### 4.2.1 Stage 1: Criteria weight determination.

In this stage, the importance weights of the evaluation criteria were determined following the procedure outlined in Section 3.2. Specifically, the Z-number-based approach was employed to capture both the perceived importance and the associated reliability of expert assessments. Take instructor T1 as an example, who rated the importance of each criterion using linguistic terms mapped to TFNs. Reliability levels, representing the evaluator’s confidence in each assessment, were similarly expressed through linguistic-to-fuzzy mappings. The detailed linguistic evaluations and their corresponding fuzzy representations are presented in Table 8.The complete linguistic evaluation tables for criteria importance (instructors T1–T5) are provided in the Supporting Information (S2 File).

**Table 8:** Linguistic evaluations of criteria importance and TFN transformations (instructor T1).

Criterion	Restriction	Reliability	Restriction (TFN)	Reliability (TFN)	Converted TFN
C1	VA	VC	(7, 9, 10)	(0.7, 0.9, 1.0)	(5.8577, 7.5218, 8.3666)
C2	VA	VC	(7, 9, 10)	(0.7, 0.9, 1.0)	(5.8577, 7.5218, 8.3666)
C3	VA	VC	(7, 9, 10)	(0.7, 0.9, 1.0)	(5.8577, 7.5218, 8.3666)
C4	A	VC	(5, 7, 9)	(0.7, 0.9, 1.0)	(4.1825, 5.8555, 7.5218)
C5	VA	AC	(7, 9, 10)	(0.9, 1.0, 1.0)	(6.6503, 8.4852, 9.4868)
C6	FA	VC	(6, 8, 10)	(0.7, 0.9, 1.0)	(5.0189, 6.6844, 8.3666)
C7	FA	VC	(6, 8, 10)	(0.7, 0.9, 1.0)	(5.0189, 6.6844, 8.3666)
C8	A	VC	(5, 7, 9)	(0.7, 0.9, 1.0)	(4.1825, 5.8555, 7.5218)
C9	A	M	(5, 7, 9)	(0.3, 0.5, 0.7)	(2.7386, 4.2862, 5.7632)
C10	VA	VC	(7, 9, 10)	(0.7, 0.9, 1.0)	(5.8577, 7.5218, 8.3666)
C11	VA	AC	(7, 9, 10)	(0.9, 1.0, 1.0)	(6.6503, 8.4852, 9.4868)
C12	FA	VC	(6, 8, 10)	(0.7, 0.9, 1.0)	(5.0189, 6.6844, 8.3666)
C13	FA	VC	(6, 8, 10)	(0.7, 0.9, 1.0)	(5.0189, 6.6844, 8.3666)
C14	VA	AC	(7, 9, 10)	(0.9, 1.0, 1.0)	(6.6503, 8.4852, 9.4868)
C15	FA	VC	(6, 8, 10)	(0.7, 0.9, 1.0)	(5.0189, 6.6844, 8.3666)

Table 8 lists the original linguistic restriction and reliability evaluations and their corresponding TFNs. The Z-number for each criterion was converted into a unified TFN according to the Eqs. (1) to (3). The resulting converted TFNs for importance assessments are shown in the “Converted TFN” column.

Following the Z-number conversion, the Entropy-CRITIC hybrid method was adopted to compute the final attribute weights. By integrating these two complementary perspectives, the final weight vector achieves greater robustness and objectivity. The resulting weights from both methods, as well as the aggregated combined weights, are summarized in Table 9 (*λ* = 0.5).

**Table 9:** Final attribute weights derived from the Entropy-CRITIC hybrid method.

	C1	C2	C3	C4	C5	C6	C7	C8	C9	C10	C11	C12	C13	C14	C15
**Entropy weight**	0.066	0.067	0.067	0.069	0.065	0.068	0.066	0.069	0.066	0.066	0.066	0.067	0.066	0.065	0.066
**CRITIC weight**	0.087	0.084	0.073	0.077	0.013	0.070	0.053	0.054	0.111	0.058	0.099	0.074	0.062	0.013	0.071
**Combined weight**	0.077	0.076	0.070	0.073	0.039	0.069	0.060	0.062	0.089	0.062	0.083	0.070	0.064	0.039	0.069

These combined weights were subsequently utilized in the Z-MABAC-based ranking stage to ensure the consistency and fairness of MCGDM under uncertainty.

#### 4.2.2 Stage 2: Students performance ranking via Z-MABAC method.

In the 2nd stage, the Z-MABAC method was employed to calculate the final performance scores and rankings of the 24 students based on the multi-source Z-number evaluations collected in the previous phase. The raw evaluation dataset comprised a total of 1,512 entries (Table 10), covering the assessments of all students across fifteen criteria (C1–C15) by various evaluators including peers, instructors, and an industry expert. Each evaluation record included the Z-number linguistic assessments for both restriction (importance) and reliability (confidence), expressed as triangular fuzzy numbers (TFNs), alongside the corresponding expert weight. The full expert evaluation dataset based on Z-number used in this study is available in the Supporting Information (S1 File).

**Table 10:** Expert evaluation data based on Z-number (Partial Data).

Criterion	Student	Evaluator	Restriction	Reliability	Restriction (TFN)	Reliability (TFN)	Individual expert weight
C1	S1	S2	F	VL	(4, 6, 8)	(0.0, 0.1, 0.3)	0.1
C1	S1	S3	FI	C	(3, 5, 7)	(0.5, 0.7, 0.9)	0.1
C1	S1	S4	A	VL	(5, 7, 9)	(0.0, 0.1, 0.3)	0.1
...	...	...	...	...	...	...	...
C15	S24	T4	A	C	(5, 7, 9)	(0.5, 0.7, 0.9)	0.2
C15	S24	T5	VA	AC	(7, 9, 10)	(0.9, 1.0, 1.0)	0.2

Following the transformation of the Z-number assessments into TFNs and the application of expert weights, a weighted normalized decision matrix was constructed, as shown in Table 11. The matrix reflects the aggregated and normalized performance of each student across the 15 criteria, incorporating both restriction and reliability through the Z-number framework. For brevity, only a subset of students’ normalized scores is presented here.

**Table 11:** Weighted normalized matrix of Z-number assessments (partial data).

Student	C1	C2	C3	C4	C5	C6	C7	C8	C9	C10	C11	C12	C13	C14	C15
S1	0.0400	0.0479	0.0258	0.0226	0.0293	0.0091	0.0322	0.0243	0.0104	0.0197	0.0159	0.0427	0.0555	0.0122	0.0574
S10	0.0076	0.0311	0.0000	0.0118	0.0000	0.0314	0.0018	0.0060	0.0199	0.0123	0.0324	0.0053	0.0026	0.0056	0.0000
S11	0.0391	0.0507	0.0452	0.0448	0.0331	0.0392	0.0371	0.0125	0.0367	0.0302	0.0196	0.0501	0.0479	0.0272	0.0453
...	...	...	...	...	...	...	...	...	...	...	...	...	...	...	...
S23	0.0447	0.0311	0.0396	0.0500	0.0349	0.0408	0.0334	0.0350	0.0481	0.0349	0.0731	0.0507	0.0453	0.0256	0.0631
S24	0.0213	0.0000	0.0407	0.0127	0.0069	0.0636	0.0127	0.0037	0.0739	0.0456	0.0560	0.0225	0.0227	0.0061	0.0549

After constructing the weighted normalized matrix, the Z-MABAC method was applied to compute the final closeness coefficients (MABAC scores) for each student. These scores reflect the integrated performance outcomes across all criteria, considering both the reliability and importance of the evaluations provided by multiple stakeholders. Table 12 presents the final results, where students are ranked according to their MABAC scores in descending order. Scores were rounded to three decimal places for reporting clarity.

**Table 12:** Final Z-MABAC scores and student ranking.

Rank	1	2	3	4	5	6	7	8	9	10	11	12
Student	S21	S9	S4	S13	S7	S23	S3	S15	S19	S5	S17	S11
Score	0.266	0.258	0.205	0.181	0.11	0.102	0.101	0.087	0.073	0.072	0.067	0.01
Rank	13	14	15	16	17	18	19	20	21	22	23	24
Student	S14	S12	S2	S18	S16	S1	S24	S22	S6	S8	S20	S10
Score	0.006	0	−0.018	−0.026	−0.044	−0.103	−0.105	−0.115	−0.179	−0.234	−0.334	−0.38

Following the ranking results obtained through the Z-MABAC method, this stage applies the grading criteria defined in Section 3.3 to categorize students into four performance bands. According to the grading policy, students falling into the top 20% are assigned Grade A, the next 45% are assigned Grade B, followed by 30% assigned Grade C, and the bottom 5% assigned Grade D. Given that the class size is *M* = 24, the detailed categorization based on MABAC scores is presented in Table 13.

**Table 13:** Grading results based on Z-MABAC ranking.

Grade	Student	Count	Proportion
A	S21, S9, S4, S13, S7	5	20.83%
B	S23, S3, S15, S19, S5, S17, S11, S14, S12, S2, S18	11	45.83%
C	S16, S1, S24, S22, S6, S8, S20	7	29.17%
D	S10	1	4.17%
E	–	0	0

**Fig 9:**
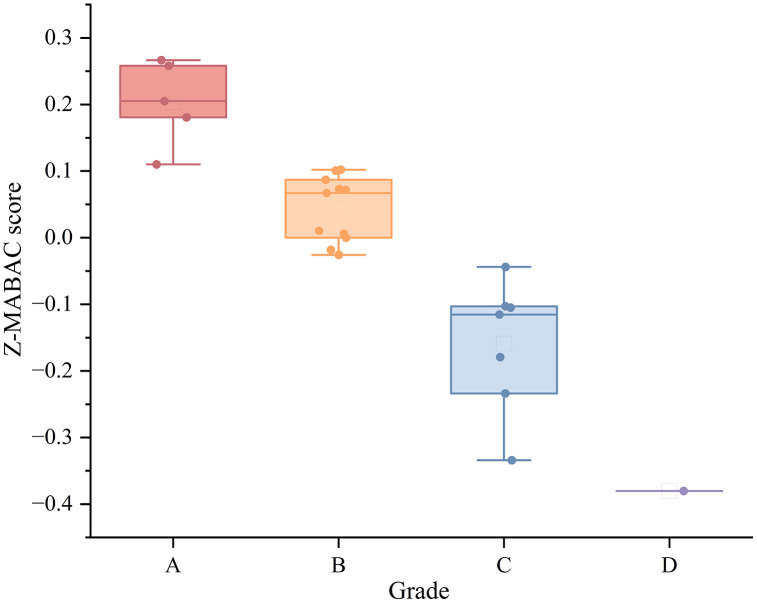
Boxplot of Z-MABAC scores across grade categories.

To further summarize the dispersion of the Z-MABAC results after grading, Fig 9 visualizes the distribution of Z-MABAC scores across grade categories. The boxplot provides a compact view of the median and variability within each grade group, based on the assignments reported in Table 13. As shown in Fig 9, Grade A corresponds to consistently positive and higher Z-MABAC scores, Grade B remains mostly positive but closer to zero with moderate spread, while Grade C shifts into the negative range with a wider dispersion. Grade D contains a single student with the lowest score, which appears as a single point.

## 5 Discussion

### 5.1 Sensitivity analysis

To evaluate the robustness of the proposed MABAC method under variations of key parameters, a comprehensive sensitivity analysis was conducted focusing on two dimensions: the variation of the confidence parameter λ in the Z-number framework, and incremental changes in individual attribute weights.

#### 5.1.1 Impact of criteria weight perturbations.

**Fig 10:**
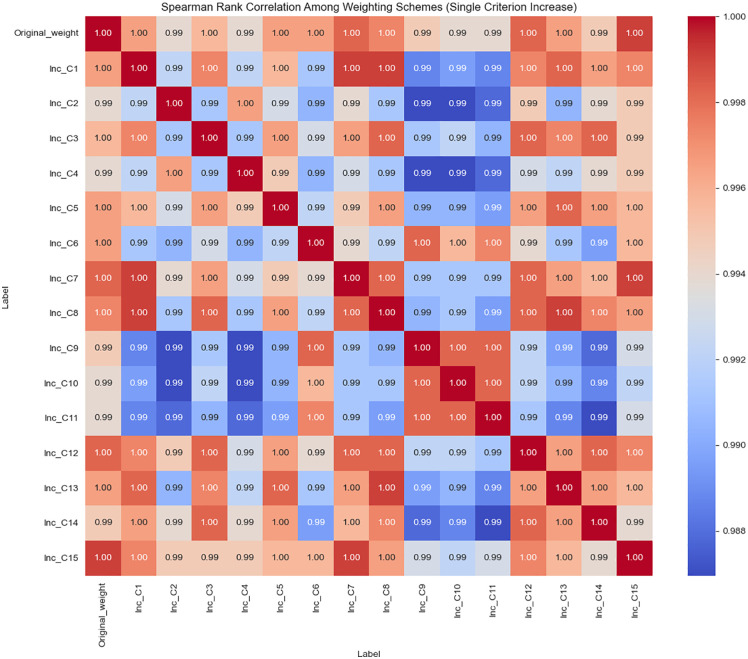
Spearman rank correlation coefficients under incremental increases of individual criterion weight.

The effect of amplifying each criterion’s weight individually was analyzed by recalculating the MABAC scores and ranks for the student alternatives. Spearman rank correlation coefficients between the original ranking and each perturbed ranking were computed to quantify stability. As shown in Fig 10, all Spearman coefficients remain above 0.98, indicating that the ranking order is highly stable against single-attribute weight increases.

**Fig 11:**
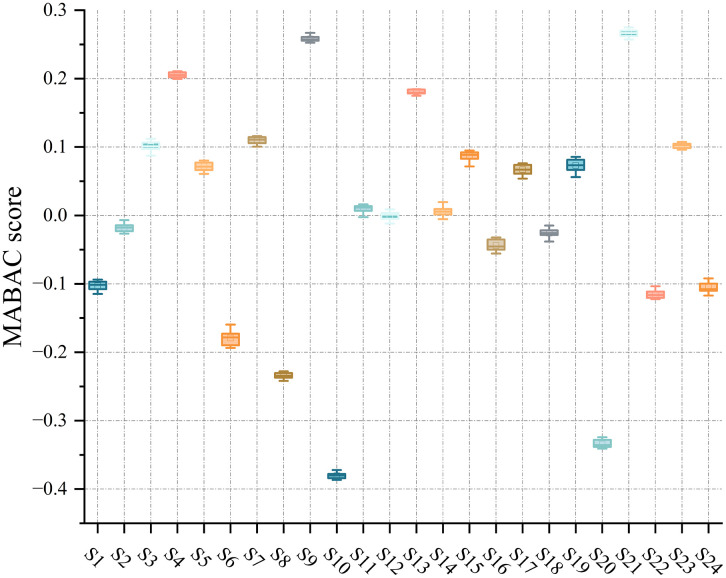
Line plot of MABAC score variations for students with individual criterion weight increment.

Correspondingly, the score variation under attribute weight increments is visualized in Fig 11, where individual student scores exhibit smooth and minor fluctuations, further confirming the robustness of MABAC with respect to moderate changes in attribute importance.

#### 5.1.2. Effect of *λ* Variation in the Z-number Model.

**Fig 12:**
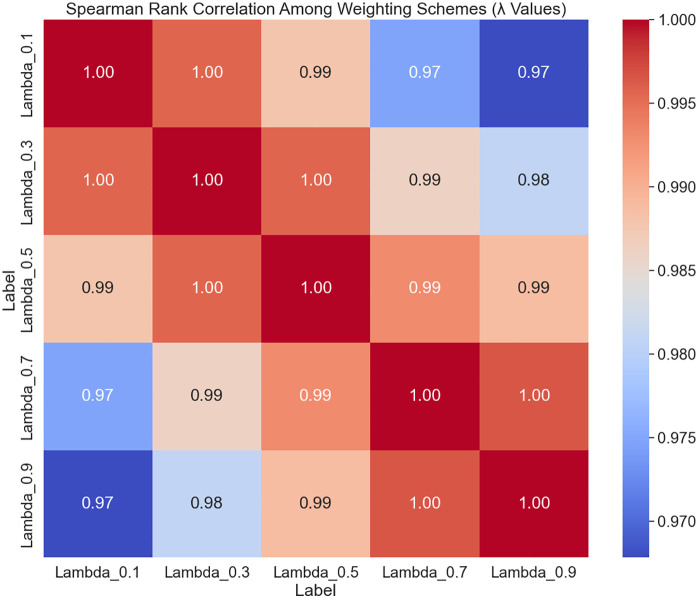
Spearman rank correlation coefficients under varying*λ* values in the Z-number model.

The confidence parameter *λ*, which balances the influence of the membership and reliability functions in the Z-number, varied systematically from 0.1 to 0.9 to assess its impact on student rankings and scores. Spearman rank correlations between rankings at different λ values and the original ranking are illustrated in Fig 12. These correlations are all above 0.99, evidencing very high rank stability across the entire λ range.

**Fig 13:**
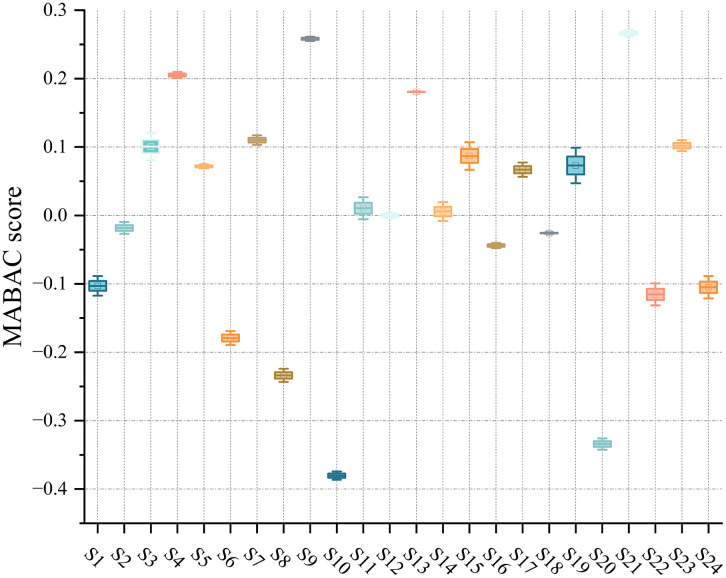
Line plot of MABAC score variations for students with different*λ* values.

The sensitivity of individual MABAC scores to *λ* changes is depicted in Fig 13. While scores vary progressively with *λ*, no abrupt changes or rank reversals occur, demonstrating that the proposed model maintains consistent evaluations even when the confidence weighting in Z-numbers is altered.

The sensitivity analyses collectively indicate that the MABAC method integrated with Z-numbers exhibits strong robustness to variations in both criterion weights and the *λ* parameter. The consistently Spearman rank correlation coefficients mostly exceeding 0.98, reflecting that the relative ordering of alternatives is largely unaffected by these perturbations. This robustness is crucial for ensuring reliable decision-making in uncertain environments where subjective weights and confidence levels may vary.

### 5.2 Comparison analysis

**Fig 14:**
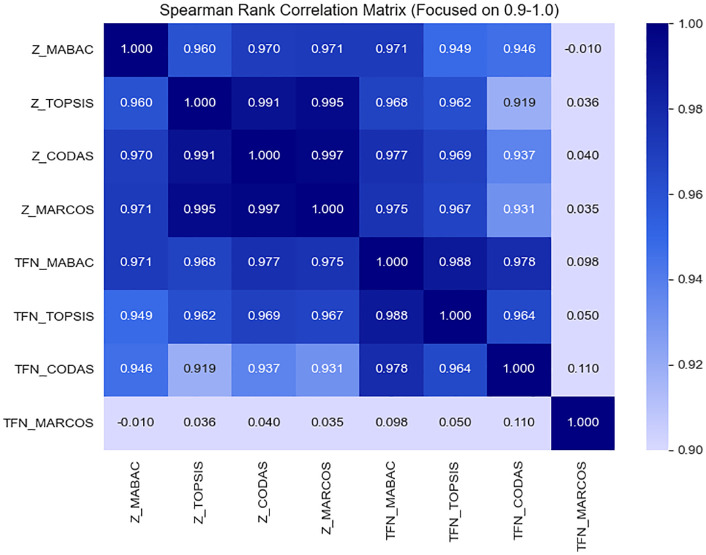
The Spearman correlation across 8 methods.

**Fig 15:**
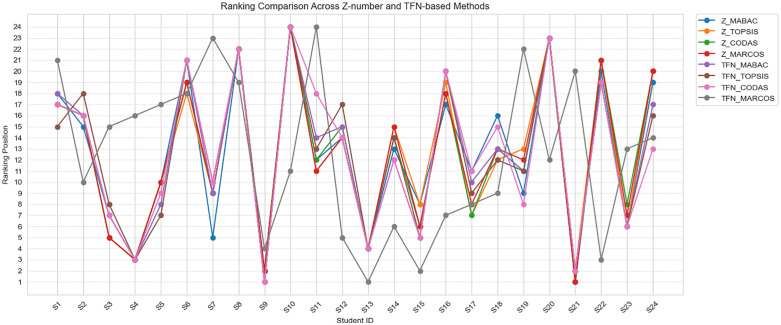
The ranking comparison of students across 8 methods.

To evaluate the consistency and discriminative capabilities of different decision-making methods, we compare eight approaches based on both Z-number and traditional TFN representations. The methods include Z-MABAC, Z-TOPSIS, Z-CODAS, Z-MARCOS, and their corresponding TFN-based versions. The Spearman rank correlation matrix is presented in Fig 14, while the ranking comparison of students across all methods is illustrated in Fig 15.

#### 5.2.1 Consistency analysis via Spearman correlation.

As shown in Fig 15, the Spearman rank correlation coefficients among the Z-number-based methods are exceptionally high, all exceeding 0.99, indicating an outstanding level of agreement. In particular, Z-MABAC shares a perfect or near-perfect correlation with Z-TOPSIS (*ρ* = 0.998), Z-CODAS (*ρ* = 0.997), and Z-MARCOS (*ρ* = 0.996). This high degree of consistency demonstrates the reliability of the Z-number framework in producing stable and coherent ranking outputs across different MCGDM techniques.

In contrast, TFN-based methods only consider the restrictions, exhibit relatively lower correlations, especially TFN-MARCOS, which shows a noticeably weaker correlation with both Z-number and other TFN approaches. This indicates that ignoring the reliability component in fuzzy evaluations may lead to inconsistencies in ranking, particularly in edge cases where uncertainty plays a significant role.

#### 5.2.2 Top and bottom rank consistency.

Fig 15 reveals that the highest-ranked student under Z-MABAC is S21, which is fully consistent with Z-CODAS and Z-MARCOS, while S9 is consistently ranked second or first in most methods. Conversely, S10 is ranked the lowest (rank 24) across all eight methods, showing excellent agreement on the least preferred alternative.

Such alignment at the extremes indicates that both Z-number and TFN-based models are robust in identifying the best and worst-performing alternatives. However, discrepancies in middle ranks are more noticeable in TFN-MARCOS and TFN-CODAS compared to their Z-number counterparts.

#### 5.2.3 Impact of reliability modeling through Z-numbers.

The integration of reliability in Z-numbers appears to enhance the stability and coherence of the rankings. Compared to TFN-based counterparts, Z-number methods yield tighter clustering of rankings, fewer rank inversions, and higher Spearman correlations.

This evidence shows the value of incorporating the reliability of Z-numbers, which effectively modulates the fuzzy restriction set by the DM’s confidence. The improved agreement among Z-number-based methods suggests that this additional layer of information leads to more informed, trustable, and consistent evaluations.

Furthermore, in comparison to TFN-based models, Z-number methods show a better balance between sensitivity and robustness, retaining the flexibility of fuzzy modeling while mitigating subjectivity through reliability weighting.

#### 5.2.4 Computational complexity of Stage 1 and Stage 2.

To assess the computational efficiency of the methods used in this study, the complexity of Stage 1 and Stage 2 is analyzed as follows:

Stage 1 (Criteria Weight Determination): The time complexity of this stage is O(N*K), where *N* is the number of criteria and *K* is the number of evaluators. Based on the actual data in this study, with N=15 criteria and K=5 evaluators, the overall time complexity for Stage 1 is calculated to be 75.
Stage 2 (Students’ Performance Ranking via Z-MABAC Method): The time complexity of this stage is O(M*N*K), where *M* is the number of students, *N* is the number of criteria, and *K* is the number of evaluators. With M=24 students,N=15 criteria, and K=5 evaluators, the overall time complexity for Stage 2 is calculated to be 1800.

This complexity analysis highlights the computational requirements of Stage 1 and Stage 2, providing insights into their efficiency in practical applications.

### 5.3 Inter-rater reliability analysis

To complement the robustness and comparison analyses presented above, an empirical inter-rater reliability (IRR) analysis was conducted based on the evaluator-level data reported in S1 and S2 Files. Specifically, S2 File provides the linguistic evaluations of criteria importance made by instructors T1–T5, together with the corresponding TFN transformations, and was therefore used to assess agreement in the criteria-weight determination stage. In contrast, S1 File contains the evaluator-level records for the case study, including criterion, student ID, evaluator identity, restriction and reliability linguistic ratings, the corresponding TFNs, and evaluator weights. From this file, the ratings of instructors T1–T5 on the six instructor-assessed criteria (C6, C9, C10, C11, C12, and C15) across the 24 students were extracted to evaluate agreement in the teacher-evaluation stage.

Because the original judgments were expressed as linguistic terms and transformed into triangular fuzzy numbers, the centroid values of the restriction TFNs were used as numeric scores for IRR estimation. A two-way random-effects intraclass correlation coefficient (ICC) with absolute agreement was adopted, and both the single-measure ICC [ICC(2,1)] and the average-measure ICC [ICC(2,k)] were reported.

First, agreement among the five instructors (T1–T5) in the criteria-weight determination stage was examined based on the 15 criteria recorded in S2 File. As summarized in Table 14, the results yielded ICC(2,1) = 0.351 and ICC(2,5) = 0.730. This indicates that, although individual-level agreement among instructors was moderate, the aggregated evaluations of the five-member instructor panel achieved acceptable reliability for group-based criteria weighting.

**Table 14:** Inter-rater reliability results for fixed instructor panels.

Stage	Targets	Raters	ICC(2,1)	ICC(2,k)
Stage 1 (weighting)	15 criteria	5	0.351	0.730
Stage 2 (evaluation, pooled)	24 students × 6 criteria	5	0.726	0.930

Second, agreement among the same instructor panel was examined in the teacher-evaluation stage using the records in S1 File for the six instructor-assessed criteria (C6, C9, C10, C11, C12, and C15) across the 24 students. The pooled analysis over all student–criterion combinations produced ICC(2,1) = 0.726 and ICC(2,5) = 0.930, indicating strong agreement among instructors, particularly when their evaluations were aggregated. The overall IRR results for Stage 1 and Stage 2 are presented in Table 14.

A criterion-wise analysis further confirmed this pattern. As shown in Table 15, the average-measure ICC values were 0.903 for C6, 0.886 for C9, 0.897 for C10, 0.879 for C11, 0.959 for C12, and 0.973 for C15. These findings provide an empirical complement to the Z-number-based modeling of evaluator reliability. In other words, besides representing uncertainty and confidence through Z-numbers, the observed agreement among the fixed instructor panels also supports the internal consistency of the proposed framework within the present case study. However, these findings should still be interpreted in light of the single-course and single-institution context.

**Table 15:** Criterion-wise inter-rater reliability for instructor-assessed criteria.

Criterion	ICC(2,1)	ICC(2,k)
C6	0.652	0.903
C9	0.609	0.886
C10	0.635	0.897
C11	0.593	0.879
C12	0.825	0.959
C15	0.880	0.973

### 5.4 Limitations

The empirical validation in this study is based on a single cohort of 24 students from one practical course at one institution. Therefore, the present findings should be interpreted as case-based evidence of feasibility and internal robustness within the studied setting, rather than as definitive evidence of general applicability. In particular, the evaluator composition, course design, and grading policy adopted in this case may differ from those in other disciplines, institutions, or assessment settings. Accordingly, claims regarding fairness, transparency, and practical applicability should be understood as context-dependent and limited to the current course environment. Future research should examine the proposed framework across multiple cohorts, courses, institutions, and evaluator configurations to establish broader external validity.

In addition, although the present study now includes a formal empirical inter-rater reliability analysis for the fixed instructor panels involved in the criteria-weight determination stage and the teacher-evaluation stage, this empirical check remains limited to comparable evaluator sets with complete repeated-rating structures. Other components of the framework, such as peer evaluations and single-expert judgments, were not examined through the same ICC-based design. Therefore, the reported IRR results should be understood as a partial empirical complement to the Z-number-based reliability modeling, rather than a full agreement assessment across all evaluator types. Future research should extend empirical agreement analysis to more diverse evaluator configurations and educational settings to further strengthen the empirical basis of the framework.

A further limitation concerns the elicitation of confidence information in the Z-number assessments. In the current implementation, some reliability inputs depend on evaluators’ self-reported confidence expressed through linguistic terms. Although this design is consistent with the conceptual structure of Z-numbers, such confidence judgments may still be affected by individual response tendencies, subjective bias, or differences in evaluators’ interpretation of linguistic labels. In addition, the final grade allocation in this case study follows an institution-specific proportion-based grading policy, which may not be directly transferable to all educational contexts. Future studies may improve the framework by combining self-reported reliability with empirical calibration strategies, behavioral indicators, or alternative grading policies that are better aligned with different institutional requirements.

## 6 Conclusion

This study proposes a novel Z-number-based multi-stage assessment framework for PBL practical courses. By integrating Z-number modeling, an Entropy-CRITIC weighting scheme, and the Z-MABAC ranking method, the framework provides a structured procedure for combining multi-source evaluations under uncertainty and varying levels of confidence. Within the studied course context, the results indicate that the proposed framework can support a more transparent, reliability-aware, and systematic assessment process than conventional single-dimensional grading approaches.

The case study demonstrates the feasibility of applying the framework to a real PBL course involving multiple evaluator roles, including instructors, peer groups, and an industry expert. The sensitivity and comparative analyses further show that the ranking results remain relatively stable under the tested settings, suggesting internal robustness of the proposed procedure within this specific application. In this sense, the framework offers a practical way to organize process-oriented and outcome-oriented assessment information in a unified decision-making structure.

At the same time, the findings should be interpreted with appropriate caution. The empirical evidence is derived from a single cohort of 24 students in one course at one institution, and the study does not yet provide broader external validation across different educational settings. In addition, although evaluator reliability is modeled through Z-numbers, further empirical work is needed to complement this representation with formal agreement measures and wider replication. Therefore, the present study should be regarded as a context-specific demonstration of feasibility rather than conclusive evidence of general applicability.

Overall, the proposed two-stage Z-number-based MCGDM framework provides a structured approach for incorporating both evaluation restrictions and reliability information into student performance assessment. The sensitivity and comparison analyses suggest that the framework can generate stable and coherent ranking results, and the additional inter-rater reliability analysis offers empirical support for the consistency of the fixed instructor panels involved in this case study. Future research should examine the framework across multiple cohorts, courses, institutions, and evaluator configurations, and further extend empirical agreement analysis to more diverse assessment settings.

## Supporting information

S1 File Expert evaluation data based on Z-number. Anonymized expert evaluation dataset used in this study, including each criterion’s restriction/reliability linguistic ratings, their corresponding triangular fuzzy numbers (TFNs), and expert weights.
(PDF)

S2 File Linguistic evaluations of criteria importance (instructors T1–T5). Anonymized linguistic evaluation tables for criteria importance provided by instructors (T1–T5), together with the corresponding TFN mappings for restriction and reliability.
(PDF)
