# Supplementary material for: A novel Z-number based multi-stage assessment framework for problem-based learning in practical courses
Source: PLoS One. 2026 May 18;21(5):e0349114. doi: 10.1371/journal.pone.0349114 (PMC13183222; doi:10.1371/journal.pone.0349114)
Supplement: S1 File — Anonymized expert evaluation dataset used in this study, including each criterion’s restriction/reliability linguistic ratings, their corresponding triangular fuzzy numbers (TFNs), and expert weights. (PDF) [file pone.0349114.s001.pdf]

Expert evaluation data based on Z-number

Criterion	Student_ID	Evaluator	Restriction	Reliability	Restriction_TFN	Reliability_TFN	Expert_weight
C1	S1	S2	F	VL	(4, 6, 8)	(0.0, 0.1, 0.3)	0.1
C1	S1	S3	FI	C	(3, 5, 7)	(0.5, 0.7, 0.9)	0.1
C1	S1	S4	A	VL	(5, 7, 9)	(0.0, 0.1, 0.3)	0.1
C1	S1	S5	VA	C	(7, 9, 10)	(0.5, 0.7, 0.9)	0.1
C1	S1	S6	F	VC	(4, 6, 8)	(0.7, 0.9, 1.0)	0.1
C1	S1	I	A	VC	(5, 7, 9)	(0.7, 0.9, 1.0)	0.5
C2	S1	I	FA	C	(6, 8, 10)	(0.5, 0.7, 0.9)	1
C3	S1	S2	F	C	(4, 6, 8)	(0.5, 0.7, 0.9)	0.1
C3	S1	S3	FA	VL	(6, 8, 10)	(0.0, 0.1, 0.3)	0.1
C3	S1	S4	VI	M	(1, 1, 3)	(0.3, 0.5, 0.7)	0.1
C3	S1	S5	I	L	(2, 4, 6)	(0.1, 0.3, 0.5)	0.1
C3	S1	S6	FI	VL	(3, 5, 7)	(0.0, 0.1, 0.3)	0.1
C3	S1	I	F	VC	(4, 6, 8)	(0.7, 0.9, 1.0)	0.5
C4	S1	I	A	M	(5, 7, 9)	(0.3, 0.5, 0.7)	1
C5	S1	I	FA	VC	(6, 8, 10)	(0.7, 0.9, 1.0)	1
C6	S1	T1	F	C	(4, 6, 8)	(0.5, 0.7, 0.9)	0.12
C6	S1	T2	F	C	(4, 6, 8)	(0.5, 0.7, 0.9)	0.12
C6	S1	T3	FI	M	(3, 5, 7)	(0.3, 0.5, 0.7)	0.12
C6	S1	T4	FI	VC	(3, 5, 7)	(0.7, 0.9, 1.0)	0.12
C6	S1	T5	F	AC	(4, 6, 8)	(0.9, 1.0, 1.0)	0.12
C6	S1	IE	FI	C	(3, 5, 7)	(0.5, 0.7, 0.9)	0.4
C7	S1	S2	I	AC	(2, 4, 6)	(0.9, 1.0, 1.0)	0.1
C7	S1	S3	FA	C	(6, 8, 10)	(0.5, 0.7, 0.9)	0.1
C7	S1	S4	FA	C	(6, 8, 10)	(0.5, 0.7, 0.9)	0.1
C7	S1	S5	FA	VC	(6, 8, 10)	(0.7, 0.9, 1.0)	0.1
C7	S1	S6	A	VL	(5, 7, 9)	(0.0, 0.1, 0.3)	0.1
C7	S1	I	A	M	(5, 7, 9)	(0.3, 0.5, 0.7)	0.5
C8	S1	S2	FA	VL	(6, 8, 10)	(0.0, 0.1, 0.3)	0.1
C8	S1	S3	FI	M	(3, 5, 7)	(0.3, 0.5, 0.7)	0.1
C8	S1	S4	FA	L	(6, 8, 10)	(0.1, 0.3, 0.5)	0.1
C8	S1	S5	A	L	(5, 7, 9)	(0.1, 0.3, 0.5)	0.1
C8	S1	S6	A	VC	(5, 7, 9)	(0.7, 0.9, 1.0)	0.1
C8	S1	I	F	AC	(4, 6, 8)	(0.9, 1.0, 1.0)	0.5
C9	S1	T1	F	M	(4, 6, 8)	(0.3, 0.5, 0.7)	0.12
C9	S1	T2	FI	VC	(3, 5, 7)	(0.7, 0.9, 1.0)	0.12
C9	S1	T3	I	C	(2, 4, 6)	(0.5, 0.7, 0.9)	0.12
C9	S1	T4	F	VC	(4, 6, 8)	(0.7, 0.9, 1.0)	0.12
C9	S1	T5	FI	M	(3, 5, 7)	(0.3, 0.5, 0.7)	0.12
C9	S1	IE	A	M	(5, 7, 9)	(0.3, 0.5, 0.7)	0.4
C10	S1	T1	F	C	(4, 6, 8)	(0.5, 0.7, 0.9)	0.12
C10	S1	T2	A	AC	(5, 7, 9)	(0.9, 1.0, 1.0)	0.12
C10	S1	T3	A	AC	(5, 7, 9)	(0.9, 1.0, 1.0)	0.12
C10	S1	T4	F	M	(4, 6, 8)	(0.3, 0.5, 0.7)	0.12
C10	S1	T5	F	M	(4, 6, 8)	(0.3, 0.5, 0.7)	0.12
C10	S1	IE	F	AC	(4, 6, 8)	(0.9, 1.0, 1.0)	0.4
C11	S1	T1	I	M	(2, 4, 6)	(0.3, 0.5, 0.7)	0.2

C11	S1	T2	F	C	(4, 6, 8)	(0.5, 0.7, 0.9)	0.2
C11	S1	T3	FI	C	(3, 5, 7)	(0.5, 0.7, 0.9)	0.2
C11	S1	T4	F	VC	(4, 6, 8)	(0.7, 0.9, 1.0)	0.2
C11	S1	T5	FI	AC	(3, 5, 7)	(0.9, 1.0, 1.0)	0.2
C12	S1	T1	A	M	(5, 7, 9)	(0.3, 0.5, 0.7)	0.12
C12	S1	T2	VA	VC	(7, 9, 10)	(0.7, 0.9, 1.0)	0.12
C12	S1	T3	FA	C	(6, 8, 10)	(0.5, 0.7, 0.9)	0.12
C12	S1	T4	FA	AC	(6, 8, 10)	(0.9, 1.0, 1.0)	0.12
C12	S1	T5	FA	C	(6, 8, 10)	(0.5, 0.7, 0.9)	0.12
C12	S1	IE	FA	M	(6, 8, 10)	(0.3, 0.5, 0.7)	0.4
C13	S1	I	FA	VC	(6, 8, 10)	(0.7, 0.9, 1.0)	1
C14	S1	I	A	M	(5, 7, 9)	(0.3, 0.5, 0.7)	1
C15	S1	T1	VA	M	(7, 9, 10)	(0.3, 0.5, 0.7)	0.2
C15	S1	T2	FA	AC	(6, 8, 10)	(0.9, 1.0, 1.0)	0.2
C15	S1	T3	FA	AC	(6, 8, 10)	(0.9, 1.0, 1.0)	0.2
C15	S1	T4	FA	M	(6, 8, 10)	(0.3, 0.5, 0.7)	0.2
C15	S1	T5	FA	AC	(6, 8, 10)	(0.9, 1.0, 1.0)	0.2
C1	S2	S1	F	M	(4, 6, 8)	(0.3, 0.5, 0.7)	0.1
C1	S2	S3	FI	M	(3, 5, 7)	(0.3, 0.5, 0.7)	0.1
C1	S2	S4	F	AC	(4, 6, 8)	(0.9, 1.0, 1.0)	0.1
C1	S2	S5	A	VL	(5, 7, 9)	(0.0, 0.1, 0.3)	0.1
C1	S2	S6	FI	M	(3, 5, 7)	(0.3, 0.5, 0.7)	0.1
C1	S2	I	FI	M	(3, 5, 7)	(0.3, 0.5, 0.7)	0.5
C2	S2	I	A	VC	(5, 7, 9)	(0.7, 0.9, 1.0)	1
C3	S2	S1	FI	L	(3, 5, 7)	(0.1, 0.3, 0.5)	0.1
C3	S2	S3	F	AC	(4, 6, 8)	(0.9, 1.0, 1.0)	0.1
C3	S2	S4	F	AC	(4, 6, 8)	(0.9, 1.0, 1.0)	0.1
C3	S2	S5	A	AC	(5, 7, 9)	(0.9, 1.0, 1.0)	0.1
C3	S2	S6	I	VL	(2, 4, 6)	(0.0, 0.1, 0.3)	0.1
C3	S2	I	I	VC	(2, 4, 6)	(0.7, 0.9, 1.0)	0.5
C4	S2	I	A	C	(5, 7, 9)	(0.5, 0.7, 0.9)	1
C5	S2	I	A	M	(5, 7, 9)	(0.3, 0.5, 0.7)	1
C6	S2	T1	FA	VC	(6, 8, 10)	(0.7, 0.9, 1.0)	0.12
C6	S2	T2	A	VC	(5, 7, 9)	(0.7, 0.9, 1.0)	0.12
C6	S2	T3	VA	C	(7, 9, 10)	(0.5, 0.7, 0.9)	0.12
C6	S2	T4	FA	AC	(6, 8, 10)	(0.9, 1.0, 1.0)	0.12
C6	S2	T5	FA	AC	(6, 8, 10)	(0.9, 1.0, 1.0)	0.12
C6	S2	IE	FA	C	(6, 8, 10)	(0.5, 0.7, 0.9)	0.4
C7	S2	S1	I	L	(2, 4, 6)	(0.1, 0.3, 0.5)	0.1
C7	S2	S3	VA	VL	(7, 9, 10)	(0.0, 0.1, 0.3)	0.1
C7	S2	S4	FI	AC	(3, 5, 7)	(0.9, 1.0, 1.0)	0.1
C7	S2	S5	F	C	(4, 6, 8)	(0.5, 0.7, 0.9)	0.1
C7	S2	S6	VI	VL	(1, 1, 3)	(0.0, 0.1, 0.3)	0.1
C7	S2	I	F	VC	(4, 6, 8)	(0.7, 0.9, 1.0)	0.5
C8	S2	S1	I	L	(2, 4, 6)	(0.1, 0.3, 0.5)	0.1
C8	S2	S3	F	VL	(4, 6, 8)	(0.0, 0.1, 0.3)	0.1
C8	S2	S4	FI	L	(3, 5, 7)	(0.1, 0.3, 0.5)	0.1
C8	S2	S5	I	VL	(2, 4, 6)	(0.0, 0.1, 0.3)	0.1
C8	S2	S6	I	M	(2, 4, 6)	(0.3, 0.5, 0.7)	0.1
C8	S2	I	F	VC	(4, 6, 8)	(0.7, 0.9, 1.0)	0.5
C9	S2	T1	F	AC	(4, 6, 8)	(0.9, 1.0, 1.0)	0.12

C9	S2	T2	F	AC	(4, 6, 8)	(0.9, 1.0, 1.0)	0.12
C9	S2	T3	FA	VC	(6, 8, 10)	(0.7, 0.9, 1.0)	0.12
C9	S2	T4	VA	M	(7, 9, 10)	(0.3, 0.5, 0.7)	0.12
C9	S2	T5	A	C	(5, 7, 9)	(0.5, 0.7, 0.9)	0.12
C9	S2	IE	F	VC	(4, 6, 8)	(0.7, 0.9, 1.0)	0.4
C10	S2	T1	FA	VC	(6, 8, 10)	(0.7, 0.9, 1.0)	0.12
C10	S2	T2	FA	M	(6, 8, 10)	(0.3, 0.5, 0.7)	0.12
C10	S2	T3	FA	VC	(6, 8, 10)	(0.7, 0.9, 1.0)	0.12
C10	S2	T4	FA	VC	(6, 8, 10)	(0.7, 0.9, 1.0)	0.12
C10	S2	T5	FA	M	(6, 8, 10)	(0.3, 0.5, 0.7)	0.12
C10	S2	IE	FA	VC	(6, 8, 10)	(0.7, 0.9, 1.0)	0.4
C11	S2	T1	A	AC	(5, 7, 9)	(0.9, 1.0, 1.0)	0.2
C11	S2	T2	FA	AC	(6, 8, 10)	(0.9, 1.0, 1.0)	0.2
C11	S2	T3	A	AC	(5, 7, 9)	(0.9, 1.0, 1.0)	0.2
C11	S2	T4	A	AC	(5, 7, 9)	(0.9, 1.0, 1.0)	0.2
C11	S2	T5	VA	VC	(7, 9, 10)	(0.7, 0.9, 1.0)	0.2
C12	S2	T1	FI	C	(3, 5, 7)	(0.5, 0.7, 0.9)	0.12
C12	S2	T2	F	AC	(4, 6, 8)	(0.9, 1.0, 1.0)	0.12
C12	S2	T3	F	AC	(4, 6, 8)	(0.9, 1.0, 1.0)	0.12
C12	S2	T4	FA	VC	(6, 8, 10)	(0.7, 0.9, 1.0)	0.12
C12	S2	T5	FA	VC	(6, 8, 10)	(0.7, 0.9, 1.0)	0.12
C12	S2	IE	FA	M	(6, 8, 10)	(0.3, 0.5, 0.7)	0.4
C13	S2	I	A	AC	(5, 7, 9)	(0.9, 1.0, 1.0)	1
C14	S2	I	VA	M	(7, 9, 10)	(0.3, 0.5, 0.7)	1
C15	S2	T1	F	AC	(4, 6, 8)	(0.9, 1.0, 1.0)	0.2
C15	S2	T2	A	C	(5, 7, 9)	(0.5, 0.7, 0.9)	0.2
C15	S2	T3	F	VC	(4, 6, 8)	(0.7, 0.9, 1.0)	0.2
C15	S2	T4	F	VC	(4, 6, 8)	(0.7, 0.9, 1.0)	0.2
C15	S2	T5	F	M	(4, 6, 8)	(0.3, 0.5, 0.7)	0.2
C1	S3	S1	FA	VL	(6, 8, 10)	(0.0, 0.1, 0.3)	0.1
C1	S3	S2	VA	M	(7, 9, 10)	(0.3, 0.5, 0.7)	0.1
C1	S3	S4	FA	AC	(6, 8, 10)	(0.9, 1.0, 1.0)	0.1
C1	S3	S5	FA	M	(6, 8, 10)	(0.3, 0.5, 0.7)	0.1
C1	S3	S6	VA	M	(7, 9, 10)	(0.3, 0.5, 0.7)	0.1
C1	S3	I	F	AC	(4, 6, 8)	(0.9, 1.0, 1.0)	0.5
C2	S3	I	VA	AC	(7, 9, 10)	(0.9, 1.0, 1.0)	1
C3	S3	S1	FA	L	(6, 8, 10)	(0.1, 0.3, 0.5)	0.1
C3	S3	S2	FA	AC	(6, 8, 10)	(0.9, 1.0, 1.0)	0.1
C3	S3	S4	A	C	(5, 7, 9)	(0.5, 0.7, 0.9)	0.1
C3	S3	S5	A	M	(5, 7, 9)	(0.3, 0.5, 0.7)	0.1
C3	S3	S6	FA	C	(6, 8, 10)	(0.5, 0.7, 0.9)	0.1
C3	S3	I	FA	M	(6, 8, 10)	(0.3, 0.5, 0.7)	0.5
C4	S3	I	FA	VC	(6, 8, 10)	(0.7, 0.9, 1.0)	1
C5	S3	I	VA	AC	(7, 9, 10)	(0.9, 1.0, 1.0)	1
C6	S3	T1	FA	VC	(6, 8, 10)	(0.7, 0.9, 1.0)	0.12
C6	S3	T2	FI	M	(3, 5, 7)	(0.3, 0.5, 0.7)	0.12
C6	S3	T3	F	M	(4, 6, 8)	(0.3, 0.5, 0.7)	0.12
C6	S3	T4	FA	AC	(6, 8, 10)	(0.9, 1.0, 1.0)	0.12
C6	S3	T5	A	VC	(5, 7, 9)	(0.7, 0.9, 1.0)	0.12
C6	S3	IE	A	C	(5, 7, 9)	(0.5, 0.7, 0.9)	0.4
C7	S3	S1	A	VL	(5, 7, 9)	(0.0, 0.1, 0.3)	0.1

C7	S3	S2	F	L	(4, 6, 8)	(0.1, 0.3, 0.5)	0.1
C7	S3	S4	A	VC	(5, 7, 9)	(0.7, 0.9, 1.0)	0.1
C7	S3	S5	VA	AC	(7, 9, 10)	(0.9, 1.0, 1.0)	0.1
C7	S3	S6	A	C	(5, 7, 9)	(0.5, 0.7, 0.9)	0.1
C7	S3	I	FA	M	(6, 8, 10)	(0.3, 0.5, 0.7)	0.5
C8	S3	S1	F	L	(4, 6, 8)	(0.1, 0.3, 0.5)	0.1
C8	S3	S2	FA	VL	(6, 8, 10)	(0.0, 0.1, 0.3)	0.1
C8	S3	S4	A	L	(5, 7, 9)	(0.1, 0.3, 0.5)	0.1
C8	S3	S5	A	AC	(5, 7, 9)	(0.9, 1.0, 1.0)	0.1
C8	S3	S6	FI	M	(3, 5, 7)	(0.3, 0.5, 0.7)	0.1
C8	S3	I	F	VC	(4, 6, 8)	(0.7, 0.9, 1.0)	0.5
C9	S3	T1	F	AC	(4, 6, 8)	(0.9, 1.0, 1.0)	0.12
C9	S3	T2	FI	M	(3, 5, 7)	(0.3, 0.5, 0.7)	0.12
C9	S3	T3	A	C	(5, 7, 9)	(0.5, 0.7, 0.9)	0.12
C9	S3	T4	I	AC	(2, 4, 6)	(0.9, 1.0, 1.0)	0.12
C9	S3	T5	FI	VC	(3, 5, 7)	(0.7, 0.9, 1.0)	0.12
C9	S3	IE	F	VC	(4, 6, 8)	(0.7, 0.9, 1.0)	0.4
C10	S3	T1	A	AC	(5, 7, 9)	(0.9, 1.0, 1.0)	0.12
C10	S3	T2	F	M	(4, 6, 8)	(0.3, 0.5, 0.7)	0.12
C10	S3	T3	F	C	(4, 6, 8)	(0.5, 0.7, 0.9)	0.12
C10	S3	T4	F	VC	(4, 6, 8)	(0.7, 0.9, 1.0)	0.12
C10	S3	T5	A	AC	(5, 7, 9)	(0.9, 1.0, 1.0)	0.12
C10	S3	IE	FA	VC	(6, 8, 10)	(0.7, 0.9, 1.0)	0.4
C11	S3	T1	F	VC	(4, 6, 8)	(0.7, 0.9, 1.0)	0.2
C11	S3	T2	A	C	(5, 7, 9)	(0.5, 0.7, 0.9)	0.2
C11	S3	T3	A	M	(5, 7, 9)	(0.3, 0.5, 0.7)	0.2
C11	S3	T4	A	M	(5, 7, 9)	(0.3, 0.5, 0.7)	0.2
C11	S3	T5	I	C	(2, 4, 6)	(0.5, 0.7, 0.9)	0.2
C12	S3	T1	VA	AC	(7, 9, 10)	(0.9, 1.0, 1.0)	0.12
C12	S3	T2	VA	AC	(7, 9, 10)	(0.9, 1.0, 1.0)	0.12
C12	S3	T3	VA	M	(7, 9, 10)	(0.3, 0.5, 0.7)	0.12
C12	S3	T4	VA	VC	(7, 9, 10)	(0.7, 0.9, 1.0)	0.12
C12	S3	T5	VA	VC	(7, 9, 10)	(0.7, 0.9, 1.0)	0.12
C12	S3	IE	VA	AC	(7, 9, 10)	(0.9, 1.0, 1.0)	0.4
C13	S3	I	FA	C	(6, 8, 10)	(0.5, 0.7, 0.9)	1
C14	S3	I	FA	VC	(6, 8, 10)	(0.7, 0.9, 1.0)	1
C15	S3	T1	FA	AC	(6, 8, 10)	(0.9, 1.0, 1.0)	0.2
C15	S3	T2	FA	C	(6, 8, 10)	(0.5, 0.7, 0.9)	0.2
C15	S3	T3	VA	M	(7, 9, 10)	(0.3, 0.5, 0.7)	0.2
C15	S3	T4	FA	C	(6, 8, 10)	(0.5, 0.7, 0.9)	0.2
C15	S3	T5	VA	M	(7, 9, 10)	(0.3, 0.5, 0.7)	0.2
C1	S4	S1	FA	C	(6, 8, 10)	(0.5, 0.7, 0.9)	0.1
C1	S4	S2	FI	L	(3, 5, 7)	(0.1, 0.3, 0.5)	0.1
C1	S4	S3	VA	AC	(7, 9, 10)	(0.9, 1.0, 1.0)	0.1
C1	S4	S5	FA	C	(6, 8, 10)	(0.5, 0.7, 0.9)	0.1
C1	S4	S6	FA	VL	(6, 8, 10)	(0.0, 0.1, 0.3)	0.1
C1	S4	I	A	VC	(5, 7, 9)	(0.7, 0.9, 1.0)	0.5
C2	S4	I	VA	VC	(7, 9, 10)	(0.7, 0.9, 1.0)	1
C3	S4	S1	VA	AC	(7, 9, 10)	(0.9, 1.0, 1.0)	0.1
C3	S4	S2	FA	VL	(6, 8, 10)	(0.0, 0.1, 0.3)	0.1
C3	S4	S3	VA	VC	(7, 9, 10)	(0.7, 0.9, 1.0)	0.1

C3	S4	S5	FA	VC	(6, 8, 10)	(0.7, 0.9, 1.0)	0.1
C3	S4	S6	VA	M	(7, 9, 10)	(0.3, 0.5, 0.7)	0.1
C3	S4	I	FA	VC	(6, 8, 10)	(0.7, 0.9, 1.0)	0.5
C4	S4	I	FA	C	(6, 8, 10)	(0.5, 0.7, 0.9)	1
C5	S4	I	FA	C	(6, 8, 10)	(0.5, 0.7, 0.9)	1
C6	S4	T1	FA	VC	(6, 8, 10)	(0.7, 0.9, 1.0)	0.12
C6	S4	T2	FA	C	(6, 8, 10)	(0.5, 0.7, 0.9)	0.12
C6	S4	T3	FA	M	(6, 8, 10)	(0.3, 0.5, 0.7)	0.12
C6	S4	T4	VA	C	(7, 9, 10)	(0.5, 0.7, 0.9)	0.12
C6	S4	T5	FA	C	(6, 8, 10)	(0.5, 0.7, 0.9)	0.12
C6	S4	IE	FA	C	(6, 8, 10)	(0.5, 0.7, 0.9)	0.4
C7	S4	S1	A	C	(5, 7, 9)	(0.5, 0.7, 0.9)	0.1
C7	S4	S2	F	VL	(4, 6, 8)	(0.0, 0.1, 0.3)	0.1
C7	S4	S3	VA	M	(7, 9, 10)	(0.3, 0.5, 0.7)	0.1
C7	S4	S5	A	VC	(5, 7, 9)	(0.7, 0.9, 1.0)	0.1
C7	S4	S6	FA	VC	(6, 8, 10)	(0.7, 0.9, 1.0)	0.1
C7	S4	I	VA	VC	(7, 9, 10)	(0.7, 0.9, 1.0)	0.5
C8	S4	S1	F	C	(4, 6, 8)	(0.5, 0.7, 0.9)	0.1
C8	S4	S2	A	VC	(5, 7, 9)	(0.7, 0.9, 1.0)	0.1
C8	S4	S3	FI	L	(3, 5, 7)	(0.1, 0.3, 0.5)	0.1
C8	S4	S5	VA	VC	(7, 9, 10)	(0.7, 0.9, 1.0)	0.1
C8	S4	S6	FA	VL	(6, 8, 10)	(0.0, 0.1, 0.3)	0.1
C8	S4	I	A	M	(5, 7, 9)	(0.3, 0.5, 0.7)	0.5
C9	S4	T1	A	VC	(5, 7, 9)	(0.7, 0.9, 1.0)	0.12
C9	S4	T2	FA	AC	(6, 8, 10)	(0.9, 1.0, 1.0)	0.12
C9	S4	T3	FA	AC	(6, 8, 10)	(0.9, 1.0, 1.0)	0.12
C9	S4	T4	VA	AC	(7, 9, 10)	(0.9, 1.0, 1.0)	0.12
C9	S4	T5	A	AC	(5, 7, 9)	(0.9, 1.0, 1.0)	0.12
C9	S4	IE	FA	M	(6, 8, 10)	(0.3, 0.5, 0.7)	0.4
C10	S4	T1	FA	C	(6, 8, 10)	(0.5, 0.7, 0.9)	0.12
C10	S4	T2	FA	VC	(6, 8, 10)	(0.7, 0.9, 1.0)	0.12
C10	S4	T3	FA	M	(6, 8, 10)	(0.3, 0.5, 0.7)	0.12
C10	S4	T4	FA	AC	(6, 8, 10)	(0.9, 1.0, 1.0)	0.12
C10	S4	T5	VA	AC	(7, 9, 10)	(0.9, 1.0, 1.0)	0.12
C10	S4	IE	VA	VC	(7, 9, 10)	(0.7, 0.9, 1.0)	0.4
C11	S4	T1	FA	C	(6, 8, 10)	(0.5, 0.7, 0.9)	0.2
C11	S4	T2	F	C	(4, 6, 8)	(0.5, 0.7, 0.9)	0.2
C11	S4	T3	FA	C	(6, 8, 10)	(0.5, 0.7, 0.9)	0.2
C11	S4	T4	A	AC	(5, 7, 9)	(0.9, 1.0, 1.0)	0.2
C11	S4	T5	FA	M	(6, 8, 10)	(0.3, 0.5, 0.7)	0.2
C12	S4	T1	FA	M	(6, 8, 10)	(0.3, 0.5, 0.7)	0.12
C12	S4	T2	A	C	(5, 7, 9)	(0.5, 0.7, 0.9)	0.12
C12	S4	T3	A	VC	(5, 7, 9)	(0.7, 0.9, 1.0)	0.12
C12	S4	T4	A	C	(5, 7, 9)	(0.5, 0.7, 0.9)	0.12
C12	S4	T5	FA	C	(6, 8, 10)	(0.5, 0.7, 0.9)	0.12
C12	S4	IE	FA	VC	(6, 8, 10)	(0.7, 0.9, 1.0)	0.4
C13	S4	I	VA	C	(7, 9, 10)	(0.5, 0.7, 0.9)	1
C14	S4	I	VA	VC	(7, 9, 10)	(0.7, 0.9, 1.0)	1
C15	S4	T1	A	VC	(5, 7, 9)	(0.7, 0.9, 1.0)	0.2
C15	S4	T2	A	M	(5, 7, 9)	(0.3, 0.5, 0.7)	0.2
C15	S4	T3	FA	VC	(6, 8, 10)	(0.7, 0.9, 1.0)	0.2

C15	S4	T4	F	C	(4, 6, 8)	(0.5, 0.7, 0.9)	0.2
C15	S4	T5	A	VC	(5, 7, 9)	(0.7, 0.9, 1.0)	0.2
C1	S5	S1	FA	L	(6, 8, 10)	(0.1, 0.3, 0.5)	0.1
C1	S5	S2	VA	AC	(7, 9, 10)	(0.9, 1.0, 1.0)	0.1
C1	S5	S3	FA	M	(6, 8, 10)	(0.3, 0.5, 0.7)	0.1
C1	S5	S4	A	VC	(5, 7, 9)	(0.7, 0.9, 1.0)	0.1
C1	S5	S6	FA	VC	(6, 8, 10)	(0.7, 0.9, 1.0)	0.1
C1	S5	I	FA	M	(6, 8, 10)	(0.3, 0.5, 0.7)	0.5
C2	S5	I	F	C	(4, 6, 8)	(0.5, 0.7, 0.9)	1
C3	S5	S1	VA	VC	(7, 9, 10)	(0.7, 0.9, 1.0)	0.1
C3	S5	S2	VA	VC	(7, 9, 10)	(0.7, 0.9, 1.0)	0.1
C3	S5	S3	VA	L	(7, 9, 10)	(0.1, 0.3, 0.5)	0.1
C3	S5	S4	VA	L	(7, 9, 10)	(0.1, 0.3, 0.5)	0.1
C3	S5	S6	A	AC	(5, 7, 9)	(0.9, 1.0, 1.0)	0.1
C3	S5	I	A	VC	(5, 7, 9)	(0.7, 0.9, 1.0)	0.5
C4	S5	I	A	M	(5, 7, 9)	(0.3, 0.5, 0.7)	1
C5	S5	I	A	M	(5, 7, 9)	(0.3, 0.5, 0.7)	1
C6	S5	T1	F	M	(4, 6, 8)	(0.3, 0.5, 0.7)	0.12
C6	S5	T2	A	M	(5, 7, 9)	(0.3, 0.5, 0.7)	0.12
C6	S5	T3	A	VC	(5, 7, 9)	(0.7, 0.9, 1.0)	0.12
C6	S5	T4	F	C	(4, 6, 8)	(0.5, 0.7, 0.9)	0.12
C6	S5	T5	A	AC	(5, 7, 9)	(0.9, 1.0, 1.0)	0.12
C6	S5	IE	F	M	(4, 6, 8)	(0.3, 0.5, 0.7)	0.4
C7	S5	S1	VA	C	(7, 9, 10)	(0.5, 0.7, 0.9)	0.1
C7	S5	S2	FA	AC	(6, 8, 10)	(0.9, 1.0, 1.0)	0.1
C7	S5	S3	VA	VC	(7, 9, 10)	(0.7, 0.9, 1.0)	0.1
C7	S5	S4	VA	M	(7, 9, 10)	(0.3, 0.5, 0.7)	0.1
C7	S5	S6	VA	AC	(7, 9, 10)	(0.9, 1.0, 1.0)	0.1
C7	S5	I	A	VC	(5, 7, 9)	(0.7, 0.9, 1.0)	0.5
C8	S5	S1	VA	C	(7, 9, 10)	(0.5, 0.7, 0.9)	0.1
C8	S5	S2	VA	L	(7, 9, 10)	(0.1, 0.3, 0.5)	0.1
C8	S5	S3	FA	AC	(6, 8, 10)	(0.9, 1.0, 1.0)	0.1
C8	S5	S4	VA	C	(7, 9, 10)	(0.5, 0.7, 0.9)	0.1
C8	S5	S6	FA	M	(6, 8, 10)	(0.3, 0.5, 0.7)	0.1
C8	S5	I	A	AC	(5, 7, 9)	(0.9, 1.0, 1.0)	0.5
C9	S5	T1	F	M	(4, 6, 8)	(0.3, 0.5, 0.7)	0.12
C9	S5	T2	FA	M	(6, 8, 10)	(0.3, 0.5, 0.7)	0.12
C9	S5	T3	F	C	(4, 6, 8)	(0.5, 0.7, 0.9)	0.12
C9	S5	T4	A	AC	(5, 7, 9)	(0.9, 1.0, 1.0)	0.12
C9	S5	T5	A	AC	(5, 7, 9)	(0.9, 1.0, 1.0)	0.12
C9	S5	IE	F	AC	(4, 6, 8)	(0.9, 1.0, 1.0)	0.4
C10	S5	T1	A	AC	(5, 7, 9)	(0.9, 1.0, 1.0)	0.12
C10	S5	T2	FA	C	(6, 8, 10)	(0.5, 0.7, 0.9)	0.12
C10	S5	T3	A	VC	(5, 7, 9)	(0.7, 0.9, 1.0)	0.12
C10	S5	T4	F	C	(4, 6, 8)	(0.5, 0.7, 0.9)	0.12
C10	S5	T5	FA	VC	(6, 8, 10)	(0.7, 0.9, 1.0)	0.12
C10	S5	IE	FA	VC	(6, 8, 10)	(0.7, 0.9, 1.0)	0.4
C11	S5	T1	FA	VC	(6, 8, 10)	(0.7, 0.9, 1.0)	0.2
C11	S5	T2	A	AC	(5, 7, 9)	(0.9, 1.0, 1.0)	0.2
C11	S5	T3	F	C	(4, 6, 8)	(0.5, 0.7, 0.9)	0.2
C11	S5	T4	A	VC	(5, 7, 9)	(0.7, 0.9, 1.0)	0.2

C11	S5	T5	FA	M	(6, 8, 10)	(0.3, 0.5, 0.7)	0.2
C12	S5	T1	VA	VC	(7, 9, 10)	(0.7, 0.9, 1.0)	0.12
C12	S5	T2	VA	C	(7, 9, 10)	(0.5, 0.7, 0.9)	0.12
C12	S5	T3	VA	AC	(7, 9, 10)	(0.9, 1.0, 1.0)	0.12
C12	S5	T4	VA	M	(7, 9, 10)	(0.3, 0.5, 0.7)	0.12
C12	S5	T5	VA	VC	(7, 9, 10)	(0.7, 0.9, 1.0)	0.12
C12	S5	IE	VA	AC	(7, 9, 10)	(0.9, 1.0, 1.0)	0.4
C13	S5	I	A	C	(5, 7, 9)	(0.5, 0.7, 0.9)	1
C14	S5	I	A	C	(5, 7, 9)	(0.5, 0.7, 0.9)	1
C15	S5	T1	VA	M	(7, 9, 10)	(0.3, 0.5, 0.7)	0.2
C15	S5	T2	VA	M	(7, 9, 10)	(0.3, 0.5, 0.7)	0.2
C15	S5	T3	FA	VC	(6, 8, 10)	(0.7, 0.9, 1.0)	0.2
C15	S5	T4	VA	AC	(7, 9, 10)	(0.9, 1.0, 1.0)	0.2
C15	S5	T5	VA	AC	(7, 9, 10)	(0.9, 1.0, 1.0)	0.2
C1	S6	S1	F	L	(4, 6, 8)	(0.1, 0.3, 0.5)	0.1
C1	S6	S2	FI	L	(3, 5, 7)	(0.1, 0.3, 0.5)	0.1
C1	S6	S3	F	M	(4, 6, 8)	(0.3, 0.5, 0.7)	0.1
C1	S6	S4	FI	VC	(3, 5, 7)	(0.7, 0.9, 1.0)	0.1
C1	S6	S5	FI	AC	(3, 5, 7)	(0.9, 1.0, 1.0)	0.1
C1	S6	I	A	VC	(5, 7, 9)	(0.7, 0.9, 1.0)	0.5
C2	S6	I	FA	C	(6, 8, 10)	(0.5, 0.7, 0.9)	1
C3	S6	S1	I	L	(2, 4, 6)	(0.1, 0.3, 0.5)	0.1
C3	S6	S2	F	C	(4, 6, 8)	(0.5, 0.7, 0.9)	0.1
C3	S6	S3	F	VC	(4, 6, 8)	(0.7, 0.9, 1.0)	0.1
C3	S6	S4	F	C	(4, 6, 8)	(0.5, 0.7, 0.9)	0.1
C3	S6	S5	FA	C	(6, 8, 10)	(0.5, 0.7, 0.9)	0.1
C3	S6	I	F	VC	(4, 6, 8)	(0.7, 0.9, 1.0)	0.5
C4	S6	I	VA	AC	(7, 9, 10)	(0.9, 1.0, 1.0)	1
C5	S6	I	FA	M	(6, 8, 10)	(0.3, 0.5, 0.7)	1
C6	S6	T1	FI	M	(3, 5, 7)	(0.3, 0.5, 0.7)	0.12
C6	S6	T2	I	AC	(2, 4, 6)	(0.9, 1.0, 1.0)	0.12
C6	S6	T3	FI	AC	(3, 5, 7)	(0.9, 1.0, 1.0)	0.12
C6	S6	T4	FI	C	(3, 5, 7)	(0.5, 0.7, 0.9)	0.12
C6	S6	T5	F	C	(4, 6, 8)	(0.5, 0.7, 0.9)	0.12
C6	S6	IE	I	VC	(2, 4, 6)	(0.7, 0.9, 1.0)	0.4
C7	S6	S1	VI	VL	(1, 1, 3)	(0.0, 0.1, 0.3)	0.1
C7	S6	S2	F	VL	(4, 6, 8)	(0.0, 0.1, 0.3)	0.1
C7	S6	S3	A	M	(5, 7, 9)	(0.3, 0.5, 0.7)	0.1
C7	S6	S4	F	C	(4, 6, 8)	(0.5, 0.7, 0.9)	0.1
C7	S6	S5	FA	VC	(6, 8, 10)	(0.7, 0.9, 1.0)	0.1
C7	S6	I	F	AC	(4, 6, 8)	(0.9, 1.0, 1.0)	0.5
C8	S6	S1	FI	M	(3, 5, 7)	(0.3, 0.5, 0.7)	0.1
C8	S6	S2	VI	VC	(1, 1, 3)	(0.7, 0.9, 1.0)	0.1
C8	S6	S3	FI	VL	(3, 5, 7)	(0.0, 0.1, 0.3)	0.1
C8	S6	S4	A	VL	(5, 7, 9)	(0.0, 0.1, 0.3)	0.1
C8	S6	S5	FI	C	(3, 5, 7)	(0.5, 0.7, 0.9)	0.1
C8	S6	I	F	C	(4, 6, 8)	(0.5, 0.7, 0.9)	0.5
C9	S6	T1	FI	VC	(3, 5, 7)	(0.7, 0.9, 1.0)	0.12
C9	S6	T2	I	VC	(2, 4, 6)	(0.7, 0.9, 1.0)	0.12
C9	S6	T3	FI	AC	(3, 5, 7)	(0.9, 1.0, 1.0)	0.12
C9	S6	T4	F	VC	(4, 6, 8)	(0.7, 0.9, 1.0)	0.12

C9	S6	T5	I	M	(2, 4, 6)	(0.3, 0.5, 0.7)	0.12
C9	S6	IE	I	AC	(2, 4, 6)	(0.9, 1.0, 1.0)	0.4
C10	S6	T1	FI	M	(3, 5, 7)	(0.3, 0.5, 0.7)	0.12
C10	S6	T2	I	C	(2, 4, 6)	(0.5, 0.7, 0.9)	0.12
C10	S6	T3	F	VC	(4, 6, 8)	(0.7, 0.9, 1.0)	0.12
C10	S6	T4	F	VC	(4, 6, 8)	(0.7, 0.9, 1.0)	0.12
C10	S6	T5	A	M	(5, 7, 9)	(0.3, 0.5, 0.7)	0.12
C10	S6	IE	FI	AC	(3, 5, 7)	(0.9, 1.0, 1.0)	0.4
C11	S6	T1	FI	AC	(3, 5, 7)	(0.9, 1.0, 1.0)	0.2
C11	S6	T2	I	VC	(2, 4, 6)	(0.7, 0.9, 1.0)	0.2
C11	S6	T3	FI	M	(3, 5, 7)	(0.3, 0.5, 0.7)	0.2
C11	S6	T4	I	C	(2, 4, 6)	(0.5, 0.7, 0.9)	0.2
C11	S6	T5	FI	M	(3, 5, 7)	(0.3, 0.5, 0.7)	0.2
C12	S6	T1	F	AC	(4, 6, 8)	(0.9, 1.0, 1.0)	0.12
C12	S6	T2	A	VC	(5, 7, 9)	(0.7, 0.9, 1.0)	0.12
C12	S6	T3	F	VC	(4, 6, 8)	(0.7, 0.9, 1.0)	0.12
C12	S6	T4	F	VC	(4, 6, 8)	(0.7, 0.9, 1.0)	0.12
C12	S6	T5	F	C	(4, 6, 8)	(0.5, 0.7, 0.9)	0.12
C12	S6	IE	F	C	(4, 6, 8)	(0.5, 0.7, 0.9)	0.4
C13	S6	I	FA	AC	(6, 8, 10)	(0.9, 1.0, 1.0)	1
C14	S6	I	FA	M	(6, 8, 10)	(0.3, 0.5, 0.7)	1
C15	S6	T1	F	M	(4, 6, 8)	(0.3, 0.5, 0.7)	0.2
C15	S6	T2	FI	M	(3, 5, 7)	(0.3, 0.5, 0.7)	0.2
C15	S6	T3	FI	C	(3, 5, 7)	(0.5, 0.7, 0.9)	0.2
C15	S6	T4	FI	VC	(3, 5, 7)	(0.7, 0.9, 1.0)	0.2
C15	S6	T5	FI	AC	(3, 5, 7)	(0.9, 1.0, 1.0)	0.2
C1	S7	S8	A	VC	(5, 7, 9)	(0.7, 0.9, 1.0)	0.1
C1	S7	S9	VA	C	(7, 9, 10)	(0.5, 0.7, 0.9)	0.1
C1	S7	S10	F	L	(4, 6, 8)	(0.1, 0.3, 0.5)	0.1
C1	S7	S11	A	VC	(5, 7, 9)	(0.7, 0.9, 1.0)	0.1
C1	S7	S12	A	VC	(5, 7, 9)	(0.7, 0.9, 1.0)	0.1
C1	S7	I	A	C	(5, 7, 9)	(0.5, 0.7, 0.9)	0.5
C2	S7	I	F	VC	(4, 6, 8)	(0.7, 0.9, 1.0)	1
C3	S7	S8	FA	VC	(6, 8, 10)	(0.7, 0.9, 1.0)	0.1
C3	S7	S9	FI	M	(3, 5, 7)	(0.3, 0.5, 0.7)	0.1
C3	S7	S10	FA	L	(6, 8, 10)	(0.1, 0.3, 0.5)	0.1
C3	S7	S11	FA	C	(6, 8, 10)	(0.5, 0.7, 0.9)	0.1
C3	S7	S12	FI	VC	(3, 5, 7)	(0.7, 0.9, 1.0)	0.1
C3	S7	I	FI	VC	(3, 5, 7)	(0.7, 0.9, 1.0)	0.5
C4	S7	I	FI	VC	(3, 5, 7)	(0.7, 0.9, 1.0)	1
C5	S7	I	A	AC	(5, 7, 9)	(0.9, 1.0, 1.0)	1
C6	S7	T1	VA	VC	(7, 9, 10)	(0.7, 0.9, 1.0)	0.12
C6	S7	T2	FA	AC	(6, 8, 10)	(0.9, 1.0, 1.0)	0.12
C6	S7	T3	A	M	(5, 7, 9)	(0.3, 0.5, 0.7)	0.12
C6	S7	T4	A	VC	(5, 7, 9)	(0.7, 0.9, 1.0)	0.12
C6	S7	T5	FA	VC	(6, 8, 10)	(0.7, 0.9, 1.0)	0.12
C6	S7	IE	FA	M	(6, 8, 10)	(0.3, 0.5, 0.7)	0.4
C7	S7	S8	F	C	(4, 6, 8)	(0.5, 0.7, 0.9)	0.1
C7	S7	S9	FA	VC	(6, 8, 10)	(0.7, 0.9, 1.0)	0.1
C7	S7	S10	A	M	(5, 7, 9)	(0.3, 0.5, 0.7)	0.1
C7	S7	S11	F	AC	(4, 6, 8)	(0.9, 1.0, 1.0)	0.1

C7	S7	S12	FA	AC	(6, 8, 10)	(0.9, 1.0, 1.0)	0.1
C7	S7	I	FA	M	(6, 8, 10)	(0.3, 0.5, 0.7)	0.5
C8	S7	S8	FA	VC	(6, 8, 10)	(0.7, 0.9, 1.0)	0.1
C8	S7	S9	FA	C	(6, 8, 10)	(0.5, 0.7, 0.9)	0.1
C8	S7	S10	F	M	(4, 6, 8)	(0.3, 0.5, 0.7)	0.1
C8	S7	S11	FA	AC	(6, 8, 10)	(0.9, 1.0, 1.0)	0.1
C8	S7	S12	FA	VC	(6, 8, 10)	(0.7, 0.9, 1.0)	0.1
C8	S7	I	A	C	(5, 7, 9)	(0.5, 0.7, 0.9)	0.5
C9	S7	T1	A	M	(5, 7, 9)	(0.3, 0.5, 0.7)	0.12
C9	S7	T2	A	AC	(5, 7, 9)	(0.9, 1.0, 1.0)	0.12
C9	S7	T3	FA	C	(6, 8, 10)	(0.5, 0.7, 0.9)	0.12
C9	S7	T4	VA	AC	(7, 9, 10)	(0.9, 1.0, 1.0)	0.12
C9	S7	T5	FA	C	(6, 8, 10)	(0.5, 0.7, 0.9)	0.12
C9	S7	IE	A	C	(5, 7, 9)	(0.5, 0.7, 0.9)	0.4
C10	S7	T1	FA	M	(6, 8, 10)	(0.3, 0.5, 0.7)	0.12
C10	S7	T2	FA	M	(6, 8, 10)	(0.3, 0.5, 0.7)	0.12
C10	S7	T3	VA	C	(7, 9, 10)	(0.5, 0.7, 0.9)	0.12
C10	S7	T4	FA	M	(6, 8, 10)	(0.3, 0.5, 0.7)	0.12
C10	S7	T5	VA	AC	(7, 9, 10)	(0.9, 1.0, 1.0)	0.12
C10	S7	IE	FA	VC	(6, 8, 10)	(0.7, 0.9, 1.0)	0.4
C11	S7	T1	FA	AC	(6, 8, 10)	(0.9, 1.0, 1.0)	0.2
C11	S7	T2	FA	C	(6, 8, 10)	(0.5, 0.7, 0.9)	0.2
C11	S7	T3	A	AC	(5, 7, 9)	(0.9, 1.0, 1.0)	0.2
C11	S7	T4	A	C	(5, 7, 9)	(0.5, 0.7, 0.9)	0.2
C11	S7	T5	VA	AC	(7, 9, 10)	(0.9, 1.0, 1.0)	0.2
C12	S7	T1	VA	AC	(7, 9, 10)	(0.9, 1.0, 1.0)	0.12
C12	S7	T2	VA	M	(7, 9, 10)	(0.3, 0.5, 0.7)	0.12
C12	S7	T3	VA	C	(7, 9, 10)	(0.5, 0.7, 0.9)	0.12
C12	S7	T4	VA	C	(7, 9, 10)	(0.5, 0.7, 0.9)	0.12
C12	S7	T5	VA	VC	(7, 9, 10)	(0.7, 0.9, 1.0)	0.12
C12	S7	IE	FA	AC	(6, 8, 10)	(0.9, 1.0, 1.0)	0.4
C13	S7	I	A	AC	(5, 7, 9)	(0.9, 1.0, 1.0)	1
C14	S7	I	FI	VC	(3, 5, 7)	(0.7, 0.9, 1.0)	1
C15	S7	T1	FA	C	(6, 8, 10)	(0.5, 0.7, 0.9)	0.2
C15	S7	T2	FA	AC	(6, 8, 10)	(0.9, 1.0, 1.0)	0.2
C15	S7	T3	VA	VC	(7, 9, 10)	(0.7, 0.9, 1.0)	0.2
C15	S7	T4	A	VC	(5, 7, 9)	(0.7, 0.9, 1.0)	0.2
C15	S7	T5	FA	VC	(6, 8, 10)	(0.7, 0.9, 1.0)	0.2
C1	S8	S7	VI	VC	(1, 1, 3)	(0.7, 0.9, 1.0)	0.1
C1	S8	S9	I	C	(2, 4, 6)	(0.5, 0.7, 0.9)	0.1
C1	S8	S10	I	L	(2, 4, 6)	(0.1, 0.3, 0.5)	0.1
C1	S8	S11	A	L	(5, 7, 9)	(0.1, 0.3, 0.5)	0.1
C1	S8	S12	F	M	(4, 6, 8)	(0.3, 0.5, 0.7)	0.1
C1	S8	I	I	VC	(2, 4, 6)	(0.7, 0.9, 1.0)	0.5
C2	S8	I	A	C	(5, 7, 9)	(0.5, 0.7, 0.9)	1
C3	S8	S7	FI	M	(3, 5, 7)	(0.3, 0.5, 0.7)	0.1
C3	S8	S9	FI	M	(3, 5, 7)	(0.3, 0.5, 0.7)	0.1
C3	S8	S10	VI	AC	(1, 1, 3)	(0.9, 1.0, 1.0)	0.1
C3	S8	S11	I	L	(2, 4, 6)	(0.1, 0.3, 0.5)	0.1
C3	S8	S12	I	AC	(2, 4, 6)	(0.9, 1.0, 1.0)	0.1
C3	S8	I	FI	AC	(3, 5, 7)	(0.9, 1.0, 1.0)	0.5

C4	S8	I	F	VC	(4, 6, 8)	(0.7, 0.9, 1.0)	1
C5	S8	I	A	AC	(5, 7, 9)	(0.9, 1.0, 1.0)	1
C6	S8	T1	A	VC	(5, 7, 9)	(0.7, 0.9, 1.0)	0.12
C6	S8	T2	FI	VC	(3, 5, 7)	(0.7, 0.9, 1.0)	0.12
C6	S8	T3	A	VC	(5, 7, 9)	(0.7, 0.9, 1.0)	0.12
C6	S8	T4	F	M	(4, 6, 8)	(0.3, 0.5, 0.7)	0.12
C6	S8	T5	F	AC	(4, 6, 8)	(0.9, 1.0, 1.0)	0.12
C6	S8	IE	F	AC	(4, 6, 8)	(0.9, 1.0, 1.0)	0.4
C7	S8	S7	I	M	(2, 4, 6)	(0.3, 0.5, 0.7)	0.1
C7	S8	S9	I	L	(2, 4, 6)	(0.1, 0.3, 0.5)	0.1
C7	S8	S10	F	M	(4, 6, 8)	(0.3, 0.5, 0.7)	0.1
C7	S8	S11	I	VL	(2, 4, 6)	(0.0, 0.1, 0.3)	0.1
C7	S8	S12	F	M	(4, 6, 8)	(0.3, 0.5, 0.7)	0.1
C7	S8	I	A	M	(5, 7, 9)	(0.3, 0.5, 0.7)	0.5
C8	S8	S7	VI	M	(1, 1, 3)	(0.3, 0.5, 0.7)	0.1
C8	S8	S9	I	C	(2, 4, 6)	(0.5, 0.7, 0.9)	0.1
C8	S8	S10	VI	AC	(1, 1, 3)	(0.9, 1.0, 1.0)	0.1
C8	S8	S11	I	M	(2, 4, 6)	(0.3, 0.5, 0.7)	0.1
C8	S8	S12	F	AC	(4, 6, 8)	(0.9, 1.0, 1.0)	0.1
C8	S8	I	FI	AC	(3, 5, 7)	(0.9, 1.0, 1.0)	0.5
C9	S8	T1	A	C	(5, 7, 9)	(0.5, 0.7, 0.9)	0.12
C9	S8	T2	A	C	(5, 7, 9)	(0.5, 0.7, 0.9)	0.12
C9	S8	T3	FA	VC	(6, 8, 10)	(0.7, 0.9, 1.0)	0.12
C9	S8	T4	FA	VC	(6, 8, 10)	(0.7, 0.9, 1.0)	0.12
C9	S8	T5	F	C	(4, 6, 8)	(0.5, 0.7, 0.9)	0.12
C9	S8	IE	FI	M	(3, 5, 7)	(0.3, 0.5, 0.7)	0.4
C10	S8	T1	A	C	(5, 7, 9)	(0.5, 0.7, 0.9)	0.12
C10	S8	T2	A	C	(5, 7, 9)	(0.5, 0.7, 0.9)	0.12
C10	S8	T3	FA	M	(6, 8, 10)	(0.3, 0.5, 0.7)	0.12
C10	S8	T4	F	C	(4, 6, 8)	(0.5, 0.7, 0.9)	0.12
C10	S8	T5	A	C	(5, 7, 9)	(0.5, 0.7, 0.9)	0.12
C10	S8	IE	FA	M	(6, 8, 10)	(0.3, 0.5, 0.7)	0.4
C11	S8	T1	A	M	(5, 7, 9)	(0.3, 0.5, 0.7)	0.2
C11	S8	T2	F	AC	(4, 6, 8)	(0.9, 1.0, 1.0)	0.2
C11	S8	T3	FA	VC	(6, 8, 10)	(0.7, 0.9, 1.0)	0.2
C11	S8	T4	FI	AC	(3, 5, 7)	(0.9, 1.0, 1.0)	0.2
C11	S8	T5	A	M	(5, 7, 9)	(0.3, 0.5, 0.7)	0.2
C12	S8	T1	FI	C	(3, 5, 7)	(0.5, 0.7, 0.9)	0.12
C12	S8	T2	FI	M	(3, 5, 7)	(0.3, 0.5, 0.7)	0.12
C12	S8	T3	FI	C	(3, 5, 7)	(0.5, 0.7, 0.9)	0.12
C12	S8	T4	F	AC	(4, 6, 8)	(0.9, 1.0, 1.0)	0.12
C12	S8	T5	FI	AC	(3, 5, 7)	(0.9, 1.0, 1.0)	0.12
C12	S8	IE	FI	AC	(3, 5, 7)	(0.9, 1.0, 1.0)	0.4
C13	S8	I	FI	C	(3, 5, 7)	(0.5, 0.7, 0.9)	1
C14	S8	I	F	VC	(4, 6, 8)	(0.7, 0.9, 1.0)	1
C15	S8	T1	FI	VC	(3, 5, 7)	(0.7, 0.9, 1.0)	0.2
C15	S8	T2	I	VC	(2, 4, 6)	(0.7, 0.9, 1.0)	0.2
C15	S8	T3	FI	M	(3, 5, 7)	(0.3, 0.5, 0.7)	0.2
C15	S8	T4	FI	M	(3, 5, 7)	(0.3, 0.5, 0.7)	0.2
C15	S8	T5	I	C	(2, 4, 6)	(0.5, 0.7, 0.9)	0.2
C1	S9	S7	VA	VC	(7, 9, 10)	(0.7, 0.9, 1.0)	0.1

C1	S9	S8	FA	AC	(6, 8, 10)	(0.9, 1.0, 1.0)	0.1
C1	S9	S10	VA	AC	(7, 9, 10)	(0.9, 1.0, 1.0)	0.1
C1	S9	S11	VA	VL	(7, 9, 10)	(0.0, 0.1, 0.3)	0.1
C1	S9	S12	FA	L	(6, 8, 10)	(0.1, 0.3, 0.5)	0.1
C1	S9	I	VA	AC	(7, 9, 10)	(0.9, 1.0, 1.0)	0.5
C2	S9	I	VA	C	(7, 9, 10)	(0.5, 0.7, 0.9)	1
C3	S9	S7	VA	VL	(7, 9, 10)	(0.0, 0.1, 0.3)	0.1
C3	S9	S8	VA	C	(7, 9, 10)	(0.5, 0.7, 0.9)	0.1
C3	S9	S10	VA	C	(7, 9, 10)	(0.5, 0.7, 0.9)	0.1
C3	S9	S11	A	L	(5, 7, 9)	(0.1, 0.3, 0.5)	0.1
C3	S9	S12	VA	AC	(7, 9, 10)	(0.9, 1.0, 1.0)	0.1
C3	S9	I	FA	VC	(6, 8, 10)	(0.7, 0.9, 1.0)	0.5
C4	S9	I	VA	M	(7, 9, 10)	(0.3, 0.5, 0.7)	1
C5	S9	I	VA	M	(7, 9, 10)	(0.3, 0.5, 0.7)	1
C6	S9	T1	A	VC	(5, 7, 9)	(0.7, 0.9, 1.0)	0.12
C6	S9	T2	FA	M	(6, 8, 10)	(0.3, 0.5, 0.7)	0.12
C6	S9	T3	FA	VC	(6, 8, 10)	(0.7, 0.9, 1.0)	0.12
C6	S9	T4	FA	VC	(6, 8, 10)	(0.7, 0.9, 1.0)	0.12
C6	S9	T5	A	AC	(5, 7, 9)	(0.9, 1.0, 1.0)	0.12
C6	S9	IE	A	AC	(5, 7, 9)	(0.9, 1.0, 1.0)	0.4
C7	S9	S7	F	L	(4, 6, 8)	(0.1, 0.3, 0.5)	0.1
C7	S9	S8	VA	VC	(7, 9, 10)	(0.7, 0.9, 1.0)	0.1
C7	S9	S10	FA	C	(6, 8, 10)	(0.5, 0.7, 0.9)	0.1
C7	S9	S11	VA	M	(7, 9, 10)	(0.3, 0.5, 0.7)	0.1
C7	S9	S12	VA	L	(7, 9, 10)	(0.1, 0.3, 0.5)	0.1
C7	S9	I	VA	M	(7, 9, 10)	(0.3, 0.5, 0.7)	0.5
C8	S9	S7	VA	M	(7, 9, 10)	(0.3, 0.5, 0.7)	0.1
C8	S9	S8	VA	L	(7, 9, 10)	(0.1, 0.3, 0.5)	0.1
C8	S9	S10	VA	AC	(7, 9, 10)	(0.9, 1.0, 1.0)	0.1
C8	S9	S11	VA	C	(7, 9, 10)	(0.5, 0.7, 0.9)	0.1
C8	S9	S12	VA	AC	(7, 9, 10)	(0.9, 1.0, 1.0)	0.1
C8	S9	I	VA	VC	(7, 9, 10)	(0.7, 0.9, 1.0)	0.5
C9	S9	T1	A	C	(5, 7, 9)	(0.5, 0.7, 0.9)	0.12
C9	S9	T2	FA	M	(6, 8, 10)	(0.3, 0.5, 0.7)	0.12
C9	S9	T3	FA	VC	(6, 8, 10)	(0.7, 0.9, 1.0)	0.12
C9	S9	T4	FA	VC	(6, 8, 10)	(0.7, 0.9, 1.0)	0.12
C9	S9	T5	A	C	(5, 7, 9)	(0.5, 0.7, 0.9)	0.12
C9	S9	IE	FA	C	(6, 8, 10)	(0.5, 0.7, 0.9)	0.4
C10	S9	T1	FA	AC	(6, 8, 10)	(0.9, 1.0, 1.0)	0.12
C10	S9	T2	A	AC	(5, 7, 9)	(0.9, 1.0, 1.0)	0.12
C10	S9	T3	VA	VC	(7, 9, 10)	(0.7, 0.9, 1.0)	0.12
C10	S9	T4	FA	M	(6, 8, 10)	(0.3, 0.5, 0.7)	0.12
C10	S9	T5	FA	AC	(6, 8, 10)	(0.9, 1.0, 1.0)	0.12
C10	S9	IE	FA	M	(6, 8, 10)	(0.3, 0.5, 0.7)	0.4
C11	S9	T1	A	C	(5, 7, 9)	(0.5, 0.7, 0.9)	0.2
C11	S9	T2	FA	C	(6, 8, 10)	(0.5, 0.7, 0.9)	0.2
C11	S9	T3	FA	AC	(6, 8, 10)	(0.9, 1.0, 1.0)	0.2
C11	S9	T4	FA	VC	(6, 8, 10)	(0.7, 0.9, 1.0)	0.2
C11	S9	T5	FA	C	(6, 8, 10)	(0.5, 0.7, 0.9)	0.2
C12	S9	T1	VA	M	(7, 9, 10)	(0.3, 0.5, 0.7)	0.12
C12	S9	T2	VA	VC	(7, 9, 10)	(0.7, 0.9, 1.0)	0.12

C12	S9	T3	VA	AC	(7, 9, 10)	(0.9, 1.0, 1.0)	0.12
C12	S9	T4	VA	C	(7, 9, 10)	(0.5, 0.7, 0.9)	0.12
C12	S9	T5	VA	AC	(7, 9, 10)	(0.9, 1.0, 1.0)	0.12
C12	S9	IE	VA	VC	(7, 9, 10)	(0.7, 0.9, 1.0)	0.4
C13	S9	I	VA	VC	(7, 9, 10)	(0.7, 0.9, 1.0)	1
C14	S9	I	FA	VC	(6, 8, 10)	(0.7, 0.9, 1.0)	1
C15	S9	T1	VA	M	(7, 9, 10)	(0.3, 0.5, 0.7)	0.2
C15	S9	T2	VA	VC	(7, 9, 10)	(0.7, 0.9, 1.0)	0.2
C15	S9	T3	VA	VC	(7, 9, 10)	(0.7, 0.9, 1.0)	0.2
C15	S9	T4	VA	C	(7, 9, 10)	(0.5, 0.7, 0.9)	0.2
C15	S9	T5	VA	M	(7, 9, 10)	(0.3, 0.5, 0.7)	0.2
C1	S10	S7	FI	AC	(3, 5, 7)	(0.9, 1.0, 1.0)	0.1
C1	S10	S8	F	VL	(4, 6, 8)	(0.0, 0.1, 0.3)	0.1
C1	S10	S9	I	C	(2, 4, 6)	(0.5, 0.7, 0.9)	0.1
C1	S10	S11	I	M	(2, 4, 6)	(0.3, 0.5, 0.7)	0.1
C1	S10	S12	I	L	(2, 4, 6)	(0.1, 0.3, 0.5)	0.1
C1	S10	I	I	AC	(2, 4, 6)	(0.9, 1.0, 1.0)	0.5
C2	S10	I	F	VC	(4, 6, 8)	(0.7, 0.9, 1.0)	1
C3	S10	S7	FI	AC	(3, 5, 7)	(0.9, 1.0, 1.0)	0.1
C3	S10	S8	I	VL	(2, 4, 6)	(0.0, 0.1, 0.3)	0.1
C3	S10	S9	FI	AC	(3, 5, 7)	(0.9, 1.0, 1.0)	0.1
C3	S10	S11	VI	M	(1, 1, 3)	(0.3, 0.5, 0.7)	0.1
C3	S10	S12	I	M	(2, 4, 6)	(0.3, 0.5, 0.7)	0.1
C3	S10	I	VI	C	(1, 1, 3)	(0.5, 0.7, 0.9)	0.5
C4	S10	I	FI	C	(3, 5, 7)	(0.5, 0.7, 0.9)	1
C5	S10	I	FI	C	(3, 5, 7)	(0.5, 0.7, 0.9)	1
C6	S10	T1	F	AC	(4, 6, 8)	(0.9, 1.0, 1.0)	0.12
C6	S10	T2	F	C	(4, 6, 8)	(0.5, 0.7, 0.9)	0.12
C6	S10	T3	A	AC	(5, 7, 9)	(0.9, 1.0, 1.0)	0.12
C6	S10	T4	FI	VC	(3, 5, 7)	(0.7, 0.9, 1.0)	0.12
C6	S10	T5	F	AC	(4, 6, 8)	(0.9, 1.0, 1.0)	0.12
C6	S10	IE	F	AC	(4, 6, 8)	(0.9, 1.0, 1.0)	0.4
C7	S10	S7	VI	VC	(1, 1, 3)	(0.7, 0.9, 1.0)	0.1
C7	S10	S8	I	AC	(2, 4, 6)	(0.9, 1.0, 1.0)	0.1
C7	S10	S9	I	L	(2, 4, 6)	(0.1, 0.3, 0.5)	0.1
C7	S10	S11	F	L	(4, 6, 8)	(0.1, 0.3, 0.5)	0.1
C7	S10	S12	FI	AC	(3, 5, 7)	(0.9, 1.0, 1.0)	0.1
C7	S10	I	I	M	(2, 4, 6)	(0.3, 0.5, 0.7)	0.5
C8	S10	S7	I	VC	(2, 4, 6)	(0.7, 0.9, 1.0)	0.1
C8	S10	S8	A	C	(5, 7, 9)	(0.5, 0.7, 0.9)	0.1
C8	S10	S9	I	VC	(2, 4, 6)	(0.7, 0.9, 1.0)	0.1
C8	S10	S11	I	AC	(2, 4, 6)	(0.9, 1.0, 1.0)	0.1
C8	S10	S12	FI	M	(3, 5, 7)	(0.3, 0.5, 0.7)	0.1
C8	S10	I	FI	M	(3, 5, 7)	(0.3, 0.5, 0.7)	0.5
C9	S10	T1	FI	M	(3, 5, 7)	(0.3, 0.5, 0.7)	0.12
C9	S10	T2	F	M	(4, 6, 8)	(0.3, 0.5, 0.7)	0.12
C9	S10	T3	FI	C	(3, 5, 7)	(0.5, 0.7, 0.9)	0.12
C9	S10	T4	F	C	(4, 6, 8)	(0.5, 0.7, 0.9)	0.12
C9	S10	T5	F	C	(4, 6, 8)	(0.5, 0.7, 0.9)	0.12
C9	S10	IE	F	VC	(4, 6, 8)	(0.7, 0.9, 1.0)	0.4
C10	S10	T1	FI	AC	(3, 5, 7)	(0.9, 1.0, 1.0)	0.12

C10	S10	T2	FI	M	(3, 5, 7)	(0.3, 0.5, 0.7)	0.12
C10	S10	T3	A	C	(5, 7, 9)	(0.5, 0.7, 0.9)	0.12
C10	S10	T4	F	AC	(4, 6, 8)	(0.9, 1.0, 1.0)	0.12
C10	S10	T5	F	M	(4, 6, 8)	(0.3, 0.5, 0.7)	0.12
C10	S10	IE	F	AC	(4, 6, 8)	(0.9, 1.0, 1.0)	0.4
C11	S10	T1	F	M	(4, 6, 8)	(0.3, 0.5, 0.7)	0.2
C11	S10	T2	FI	AC	(3, 5, 7)	(0.9, 1.0, 1.0)	0.2
C11	S10	T3	FI	C	(3, 5, 7)	(0.5, 0.7, 0.9)	0.2
C11	S10	T4	F	AC	(4, 6, 8)	(0.9, 1.0, 1.0)	0.2
C11	S10	T5	A	AC	(5, 7, 9)	(0.9, 1.0, 1.0)	0.2
C12	S10	T1	I	M	(2, 4, 6)	(0.3, 0.5, 0.7)	0.12
C12	S10	T2	F	AC	(4, 6, 8)	(0.9, 1.0, 1.0)	0.12
C12	S10	T3	FI	VC	(3, 5, 7)	(0.7, 0.9, 1.0)	0.12
C12	S10	T4	I	M	(2, 4, 6)	(0.3, 0.5, 0.7)	0.12
C12	S10	T5	FI	AC	(3, 5, 7)	(0.9, 1.0, 1.0)	0.12
C12	S10	IE	I	VC	(2, 4, 6)	(0.7, 0.9, 1.0)	0.4
C13	S10	I	FI	M	(3, 5, 7)	(0.3, 0.5, 0.7)	1
C14	S10	I	FI	C	(3, 5, 7)	(0.5, 0.7, 0.9)	1
C15	S10	T1	I	M	(2, 4, 6)	(0.3, 0.5, 0.7)	0.2
C15	S10	T2	I	M	(2, 4, 6)	(0.3, 0.5, 0.7)	0.2
C15	S10	T3	FI	AC	(3, 5, 7)	(0.9, 1.0, 1.0)	0.2
C15	S10	T4	I	VC	(2, 4, 6)	(0.7, 0.9, 1.0)	0.2
C15	S10	T5	I	M	(2, 4, 6)	(0.3, 0.5, 0.7)	0.2
C1	S11	S7	A	VC	(5, 7, 9)	(0.7, 0.9, 1.0)	0.1
C1	S11	S8	FA	VL	(6, 8, 10)	(0.0, 0.1, 0.3)	0.1
C1	S11	S9	F	VL	(4, 6, 8)	(0.0, 0.1, 0.3)	0.1
C1	S11	S10	FA	C	(6, 8, 10)	(0.5, 0.7, 0.9)	0.1
C1	S11	S12	FA	VL	(6, 8, 10)	(0.0, 0.1, 0.3)	0.1
C1	S11	I	A	VC	(5, 7, 9)	(0.7, 0.9, 1.0)	0.5
C2	S11	I	A	AC	(5, 7, 9)	(0.9, 1.0, 1.0)	1
C3	S11	S7	I	L	(2, 4, 6)	(0.1, 0.3, 0.5)	0.1
C3	S11	S8	VI	L	(1, 1, 3)	(0.1, 0.3, 0.5)	0.1
C3	S11	S9	F	AC	(4, 6, 8)	(0.9, 1.0, 1.0)	0.1
C3	S11	S10	FA	AC	(6, 8, 10)	(0.9, 1.0, 1.0)	0.1
C3	S11	S12	I	AC	(2, 4, 6)	(0.9, 1.0, 1.0)	0.1
C3	S11	I	A	AC	(5, 7, 9)	(0.9, 1.0, 1.0)	0.5
C4	S11	I	A	VC	(5, 7, 9)	(0.7, 0.9, 1.0)	1
C5	S11	I	FA	AC	(6, 8, 10)	(0.9, 1.0, 1.0)	1
C6	S11	T1	A	AC	(5, 7, 9)	(0.9, 1.0, 1.0)	0.12
C6	S11	T2	F	AC	(4, 6, 8)	(0.9, 1.0, 1.0)	0.12
C6	S11	T3	A	AC	(5, 7, 9)	(0.9, 1.0, 1.0)	0.12
C6	S11	T4	F	M	(4, 6, 8)	(0.3, 0.5, 0.7)	0.12
C6	S11	T5	A	C	(5, 7, 9)	(0.5, 0.7, 0.9)	0.12
C6	S11	IE	A	VC	(5, 7, 9)	(0.7, 0.9, 1.0)	0.4
C7	S11	S7	A	VL	(5, 7, 9)	(0.0, 0.1, 0.3)	0.1
C7	S11	S8	VA	L	(7, 9, 10)	(0.1, 0.3, 0.5)	0.1
C7	S11	S9	FA	VC	(6, 8, 10)	(0.7, 0.9, 1.0)	0.1
C7	S11	S10	F	C	(4, 6, 8)	(0.5, 0.7, 0.9)	0.1
C7	S11	S12	A	VC	(5, 7, 9)	(0.7, 0.9, 1.0)	0.1
C7	S11	I	A	C	(5, 7, 9)	(0.5, 0.7, 0.9)	0.5
C8	S11	S7	F	AC	(4, 6, 8)	(0.9, 1.0, 1.0)	0.1

C8	S11	S8	VA	L	(7, 9, 10)	(0.1, 0.3, 0.5)	0.1
C8	S11	S9	A	VL	(5, 7, 9)	(0.0, 0.1, 0.3)	0.1
C8	S11	S10	I	VL	(2, 4, 6)	(0.0, 0.1, 0.3)	0.1
C8	S11	S12	FI	M	(3, 5, 7)	(0.3, 0.5, 0.7)	0.1
C8	S11	I	FI	AC	(3, 5, 7)	(0.9, 1.0, 1.0)	0.5
C9	S11	T1	F	AC	(4, 6, 8)	(0.9, 1.0, 1.0)	0.12
C9	S11	T2	FI	AC	(3, 5, 7)	(0.9, 1.0, 1.0)	0.12
C9	S11	T3	F	C	(4, 6, 8)	(0.5, 0.7, 0.9)	0.12
C9	S11	T4	FI	C	(3, 5, 7)	(0.5, 0.7, 0.9)	0.12
C9	S11	T5	A	AC	(5, 7, 9)	(0.9, 1.0, 1.0)	0.12
C9	S11	IE	F	VC	(4, 6, 8)	(0.7, 0.9, 1.0)	0.4
C10	S11	T1	A	C	(5, 7, 9)	(0.5, 0.7, 0.9)	0.12
C10	S11	T2	A	VC	(5, 7, 9)	(0.7, 0.9, 1.0)	0.12
C10	S11	T3	F	C	(4, 6, 8)	(0.5, 0.7, 0.9)	0.12
C10	S11	T4	A	M	(5, 7, 9)	(0.3, 0.5, 0.7)	0.12
C10	S11	T5	FA	M	(6, 8, 10)	(0.3, 0.5, 0.7)	0.12
C10	S11	IE	A	AC	(5, 7, 9)	(0.9, 1.0, 1.0)	0.4
C11	S11	T1	F	M	(4, 6, 8)	(0.3, 0.5, 0.7)	0.2
C11	S11	T2	FI	C	(3, 5, 7)	(0.5, 0.7, 0.9)	0.2
C11	S11	T3	F	C	(4, 6, 8)	(0.5, 0.7, 0.9)	0.2
C11	S11	T4	A	M	(5, 7, 9)	(0.3, 0.5, 0.7)	0.2
C11	S11	T5	A	M	(5, 7, 9)	(0.3, 0.5, 0.7)	0.2
C12	S11	T1	FA	M	(6, 8, 10)	(0.3, 0.5, 0.7)	0.12
C12	S11	T2	FA	AC	(6, 8, 10)	(0.9, 1.0, 1.0)	0.12
C12	S11	T3	FA	M	(6, 8, 10)	(0.3, 0.5, 0.7)	0.12
C12	S11	T4	FA	C	(6, 8, 10)	(0.5, 0.7, 0.9)	0.12
C12	S11	T5	A	M	(5, 7, 9)	(0.3, 0.5, 0.7)	0.12
C12	S11	IE	FA	AC	(6, 8, 10)	(0.9, 1.0, 1.0)	0.4
C13	S11	I	A	AC	(5, 7, 9)	(0.9, 1.0, 1.0)	1
C14	S11	I	FA	C	(6, 8, 10)	(0.5, 0.7, 0.9)	1
C15	S11	T1	FA	C	(6, 8, 10)	(0.5, 0.7, 0.9)	0.2
C15	S11	T2	FA	M	(6, 8, 10)	(0.3, 0.5, 0.7)	0.2
C15	S11	T3	F	C	(4, 6, 8)	(0.5, 0.7, 0.9)	0.2
C15	S11	T4	FA	VC	(6, 8, 10)	(0.7, 0.9, 1.0)	0.2
C15	S11	T5	FA	C	(6, 8, 10)	(0.5, 0.7, 0.9)	0.2
C1	S12	S7	A	AC	(5, 7, 9)	(0.9, 1.0, 1.0)	0.1
C1	S12	S8	A	VC	(5, 7, 9)	(0.7, 0.9, 1.0)	0.1
C1	S12	S9	A	M	(5, 7, 9)	(0.3, 0.5, 0.7)	0.1
C1	S12	S10	FA	VC	(6, 8, 10)	(0.7, 0.9, 1.0)	0.1
C1	S12	S11	I	VC	(2, 4, 6)	(0.7, 0.9, 1.0)	0.1
C1	S12	I	FI	VC	(3, 5, 7)	(0.7, 0.9, 1.0)	0.5
C2	S12	I	A	C	(5, 7, 9)	(0.5, 0.7, 0.9)	1
C3	S12	S7	VI	M	(1, 1, 3)	(0.3, 0.5, 0.7)	0.1
C3	S12	S8	A	L	(5, 7, 9)	(0.1, 0.3, 0.5)	0.1
C3	S12	S9	A	AC	(5, 7, 9)	(0.9, 1.0, 1.0)	0.1
C3	S12	S10	FI	VC	(3, 5, 7)	(0.7, 0.9, 1.0)	0.1
C3	S12	S11	FI	L	(3, 5, 7)	(0.1, 0.3, 0.5)	0.1
C3	S12	I	FI	AC	(3, 5, 7)	(0.9, 1.0, 1.0)	0.5
C4	S12	I	A	VC	(5, 7, 9)	(0.7, 0.9, 1.0)	1
C5	S12	I	A	VC	(5, 7, 9)	(0.7, 0.9, 1.0)	1
C6	S12	T1	FA	M	(6, 8, 10)	(0.3, 0.5, 0.7)	0.12

C6	S12	T2	FA	C	(6, 8, 10)	(0.5, 0.7, 0.9)	0.12
C6	S12	T3	FA	AC	(6, 8, 10)	(0.9, 1.0, 1.0)	0.12
C6	S12	T4	FA	AC	(6, 8, 10)	(0.9, 1.0, 1.0)	0.12
C6	S12	T5	A	M	(5, 7, 9)	(0.3, 0.5, 0.7)	0.12
C6	S12	IE	VA	VC	(7, 9, 10)	(0.7, 0.9, 1.0)	0.4
C7	S12	S7	I	AC	(2, 4, 6)	(0.9, 1.0, 1.0)	0.1
C7	S12	S8	F	AC	(4, 6, 8)	(0.9, 1.0, 1.0)	0.1
C7	S12	S9	FA	C	(6, 8, 10)	(0.5, 0.7, 0.9)	0.1
C7	S12	S10	FI	VC	(3, 5, 7)	(0.7, 0.9, 1.0)	0.1
C7	S12	S11	A	M	(5, 7, 9)	(0.3, 0.5, 0.7)	0.1
C7	S12	I	A	AC	(5, 7, 9)	(0.9, 1.0, 1.0)	0.5
C8	S12	S7	I	C	(2, 4, 6)	(0.5, 0.7, 0.9)	0.1
C8	S12	S8	F	L	(4, 6, 8)	(0.1, 0.3, 0.5)	0.1
C8	S12	S9	FI	M	(3, 5, 7)	(0.3, 0.5, 0.7)	0.1
C8	S12	S10	FI	C	(3, 5, 7)	(0.5, 0.7, 0.9)	0.1
C8	S12	S11	VA	L	(7, 9, 10)	(0.1, 0.3, 0.5)	0.1
C8	S12	I	FI	AC	(3, 5, 7)	(0.9, 1.0, 1.0)	0.5
C9	S12	T1	FA	C	(6, 8, 10)	(0.5, 0.7, 0.9)	0.12
C9	S12	T2	A	C	(5, 7, 9)	(0.5, 0.7, 0.9)	0.12
C9	S12	T3	A	VC	(5, 7, 9)	(0.7, 0.9, 1.0)	0.12
C9	S12	T4	A	C	(5, 7, 9)	(0.5, 0.7, 0.9)	0.12
C9	S12	T5	FA	AC	(6, 8, 10)	(0.9, 1.0, 1.0)	0.12
C9	S12	IE	FA	M	(6, 8, 10)	(0.3, 0.5, 0.7)	0.4
C10	S12	T1	VA	AC	(7, 9, 10)	(0.9, 1.0, 1.0)	0.12
C10	S12	T2	VA	AC	(7, 9, 10)	(0.9, 1.0, 1.0)	0.12
C10	S12	T3	FA	C	(6, 8, 10)	(0.5, 0.7, 0.9)	0.12
C10	S12	T4	FA	AC	(6, 8, 10)	(0.9, 1.0, 1.0)	0.12
C10	S12	T5	FA	C	(6, 8, 10)	(0.5, 0.7, 0.9)	0.12
C10	S12	IE	FA	VC	(6, 8, 10)	(0.7, 0.9, 1.0)	0.4
C11	S12	T1	FA	C	(6, 8, 10)	(0.5, 0.7, 0.9)	0.2
C11	S12	T2	A	VC	(5, 7, 9)	(0.7, 0.9, 1.0)	0.2
C11	S12	T3	VA	M	(7, 9, 10)	(0.3, 0.5, 0.7)	0.2
C11	S12	T4	FA	AC	(6, 8, 10)	(0.9, 1.0, 1.0)	0.2
C11	S12	T5	F	VC	(4, 6, 8)	(0.7, 0.9, 1.0)	0.2
C12	S12	T1	F	M	(4, 6, 8)	(0.3, 0.5, 0.7)	0.12
C12	S12	T2	A	C	(5, 7, 9)	(0.5, 0.7, 0.9)	0.12
C12	S12	T3	A	M	(5, 7, 9)	(0.3, 0.5, 0.7)	0.12
C12	S12	T4	FI	VC	(3, 5, 7)	(0.7, 0.9, 1.0)	0.12
C12	S12	T5	A	AC	(5, 7, 9)	(0.9, 1.0, 1.0)	0.12
C12	S12	IE	I	M	(2, 4, 6)	(0.3, 0.5, 0.7)	0.4
C13	S12	I	A	M	(5, 7, 9)	(0.3, 0.5, 0.7)	1
C14	S12	I	FA	C	(6, 8, 10)	(0.5, 0.7, 0.9)	1
C15	S12	T1	FI	C	(3, 5, 7)	(0.5, 0.7, 0.9)	0.2
C15	S12	T2	FI	AC	(3, 5, 7)	(0.9, 1.0, 1.0)	0.2
C15	S12	T3	F	C	(4, 6, 8)	(0.5, 0.7, 0.9)	0.2
C15	S12	T4	FI	VC	(3, 5, 7)	(0.7, 0.9, 1.0)	0.2
C15	S12	T5	F	VC	(4, 6, 8)	(0.7, 0.9, 1.0)	0.2
C1	S13	S14	A	VL	(5, 7, 9)	(0.0, 0.1, 0.3)	0.1
C1	S13	S15	A	L	(5, 7, 9)	(0.1, 0.3, 0.5)	0.1
C1	S13	S16	FI	L	(3, 5, 7)	(0.1, 0.3, 0.5)	0.1
C1	S13	S17	FA	AC	(6, 8, 10)	(0.9, 1.0, 1.0)	0.1

C1	S13	S18	FA	VL	(6, 8, 10)	(0.0, 0.1, 0.3)	0.1
C1	S13	I	A	AC	(5, 7, 9)	(0.9, 1.0, 1.0)	0.5
C2	S13	I	FA	C	(6, 8, 10)	(0.5, 0.7, 0.9)	1
C3	S13	S14	FA	AC	(6, 8, 10)	(0.9, 1.0, 1.0)	0.1
C3	S13	S15	A	VC	(5, 7, 9)	(0.7, 0.9, 1.0)	0.1
C3	S13	S16	FA	AC	(6, 8, 10)	(0.9, 1.0, 1.0)	0.1
C3	S13	S17	FA	L	(6, 8, 10)	(0.1, 0.3, 0.5)	0.1
C3	S13	S18	F	C	(4, 6, 8)	(0.5, 0.7, 0.9)	0.1
C3	S13	I	VA	C	(7, 9, 10)	(0.5, 0.7, 0.9)	0.5
C4	S13	I	FA	VC	(6, 8, 10)	(0.7, 0.9, 1.0)	1
C5	S13	I	VA	VC	(7, 9, 10)	(0.7, 0.9, 1.0)	1
C6	S13	T1	FA	AC	(6, 8, 10)	(0.9, 1.0, 1.0)	0.12
C6	S13	T2	A	AC	(5, 7, 9)	(0.9, 1.0, 1.0)	0.12
C6	S13	T3	FA	VC	(6, 8, 10)	(0.7, 0.9, 1.0)	0.12
C6	S13	T4	A	VC	(5, 7, 9)	(0.7, 0.9, 1.0)	0.12
C6	S13	T5	F	VC	(4, 6, 8)	(0.7, 0.9, 1.0)	0.12
C6	S13	IE	A	VC	(5, 7, 9)	(0.7, 0.9, 1.0)	0.4
C7	S13	S14	A	VC	(5, 7, 9)	(0.7, 0.9, 1.0)	0.1
C7	S13	S15	A	C	(5, 7, 9)	(0.5, 0.7, 0.9)	0.1
C7	S13	S16	F	C	(4, 6, 8)	(0.5, 0.7, 0.9)	0.1
C7	S13	S17	VA	VL	(7, 9, 10)	(0.0, 0.1, 0.3)	0.1
C7	S13	S18	VA	AC	(7, 9, 10)	(0.9, 1.0, 1.0)	0.1
C7	S13	I	FA	AC	(6, 8, 10)	(0.9, 1.0, 1.0)	0.5
C8	S13	S14	VA	M	(7, 9, 10)	(0.3, 0.5, 0.7)	0.1
C8	S13	S15	A	L	(5, 7, 9)	(0.1, 0.3, 0.5)	0.1
C8	S13	S16	A	M	(5, 7, 9)	(0.3, 0.5, 0.7)	0.1
C8	S13	S17	F	M	(4, 6, 8)	(0.3, 0.5, 0.7)	0.1
C8	S13	S18	FA	C	(6, 8, 10)	(0.5, 0.7, 0.9)	0.1
C8	S13	I	FA	M	(6, 8, 10)	(0.3, 0.5, 0.7)	0.5
C9	S13	T1	A	C	(5, 7, 9)	(0.5, 0.7, 0.9)	0.12
C9	S13	T2	FA	C	(6, 8, 10)	(0.5, 0.7, 0.9)	0.12
C9	S13	T3	A	M	(5, 7, 9)	(0.3, 0.5, 0.7)	0.12
C9	S13	T4	F	VC	(4, 6, 8)	(0.7, 0.9, 1.0)	0.12
C9	S13	T5	A	AC	(5, 7, 9)	(0.9, 1.0, 1.0)	0.12
C9	S13	IE	FA	VC	(6, 8, 10)	(0.7, 0.9, 1.0)	0.4
C10	S13	T1	FA	C	(6, 8, 10)	(0.5, 0.7, 0.9)	0.12
C10	S13	T2	FA	C	(6, 8, 10)	(0.5, 0.7, 0.9)	0.12
C10	S13	T3	VA	VC	(7, 9, 10)	(0.7, 0.9, 1.0)	0.12
C10	S13	T4	VA	C	(7, 9, 10)	(0.5, 0.7, 0.9)	0.12
C10	S13	T5	FA	AC	(6, 8, 10)	(0.9, 1.0, 1.0)	0.12
C10	S13	IE	A	C	(5, 7, 9)	(0.5, 0.7, 0.9)	0.4
C11	S13	T1	F	AC	(4, 6, 8)	(0.9, 1.0, 1.0)	0.2
C11	S13	T2	A	VC	(5, 7, 9)	(0.7, 0.9, 1.0)	0.2
C11	S13	T3	FA	VC	(6, 8, 10)	(0.7, 0.9, 1.0)	0.2
C11	S13	T4	FA	AC	(6, 8, 10)	(0.9, 1.0, 1.0)	0.2
C11	S13	T5	FA	AC	(6, 8, 10)	(0.9, 1.0, 1.0)	0.2
C12	S13	T1	VA	AC	(7, 9, 10)	(0.9, 1.0, 1.0)	0.12
C12	S13	T2	FA	C	(6, 8, 10)	(0.5, 0.7, 0.9)	0.12
C12	S13	T3	VA	VC	(7, 9, 10)	(0.7, 0.9, 1.0)	0.12
C12	S13	T4	VA	C	(7, 9, 10)	(0.5, 0.7, 0.9)	0.12
C12	S13	T5	A	C	(5, 7, 9)	(0.5, 0.7, 0.9)	0.12

C12	S13	IE	FA	AC	(6, 8, 10)	(0.9, 1.0, 1.0)	0.4
C13	S13	I	FA	C	(6, 8, 10)	(0.5, 0.7, 0.9)	1
C14	S13	I	FA	M	(6, 8, 10)	(0.3, 0.5, 0.7)	1
C15	S13	T1	FA	M	(6, 8, 10)	(0.3, 0.5, 0.7)	0.2
C15	S13	T2	A	M	(5, 7, 9)	(0.3, 0.5, 0.7)	0.2
C15	S13	T3	FA	M	(6, 8, 10)	(0.3, 0.5, 0.7)	0.2
C15	S13	T4	FA	M	(6, 8, 10)	(0.3, 0.5, 0.7)	0.2
C15	S13	T5	FA	AC	(6, 8, 10)	(0.9, 1.0, 1.0)	0.2
C1	S14	S13	A	L	(5, 7, 9)	(0.1, 0.3, 0.5)	0.1
C1	S14	S15	A	AC	(5, 7, 9)	(0.9, 1.0, 1.0)	0.1
C1	S14	S16	FA	L	(6, 8, 10)	(0.1, 0.3, 0.5)	0.1
C1	S14	S17	I	VL	(2, 4, 6)	(0.0, 0.1, 0.3)	0.1
C1	S14	S18	FI	AC	(3, 5, 7)	(0.9, 1.0, 1.0)	0.1
C1	S14	I	F	C	(4, 6, 8)	(0.5, 0.7, 0.9)	0.5
C2	S14	I	F	M	(4, 6, 8)	(0.3, 0.5, 0.7)	1
C3	S14	S13	A	L	(5, 7, 9)	(0.1, 0.3, 0.5)	0.1
C3	S14	S15	A	VC	(5, 7, 9)	(0.7, 0.9, 1.0)	0.1
C3	S14	S16	F	C	(4, 6, 8)	(0.5, 0.7, 0.9)	0.1
C3	S14	S17	F	M	(4, 6, 8)	(0.3, 0.5, 0.7)	0.1
C3	S14	S18	FI	VL	(3, 5, 7)	(0.0, 0.1, 0.3)	0.1
C3	S14	I	A	AC	(5, 7, 9)	(0.9, 1.0, 1.0)	0.5
C4	S14	I	FI	AC	(3, 5, 7)	(0.9, 1.0, 1.0)	1
C5	S14	I	A	M	(5, 7, 9)	(0.3, 0.5, 0.7)	1
C6	S14	T1	FA	M	(6, 8, 10)	(0.3, 0.5, 0.7)	0.12
C6	S14	T2	FA	AC	(6, 8, 10)	(0.9, 1.0, 1.0)	0.12
C6	S14	T3	FA	M	(6, 8, 10)	(0.3, 0.5, 0.7)	0.12
C6	S14	T4	A	M	(5, 7, 9)	(0.3, 0.5, 0.7)	0.12
C6	S14	T5	FA	VC	(6, 8, 10)	(0.7, 0.9, 1.0)	0.12
C6	S14	IE	FA	VC	(6, 8, 10)	(0.7, 0.9, 1.0)	0.4
C7	S14	S13	I	VL	(2, 4, 6)	(0.0, 0.1, 0.3)	0.1
C7	S14	S15	A	VC	(5, 7, 9)	(0.7, 0.9, 1.0)	0.1
C7	S14	S16	FA	AC	(6, 8, 10)	(0.9, 1.0, 1.0)	0.1
C7	S14	S17	FA	AC	(6, 8, 10)	(0.9, 1.0, 1.0)	0.1
C7	S14	S18	F	C	(4, 6, 8)	(0.5, 0.7, 0.9)	0.1
C7	S14	I	A	C	(5, 7, 9)	(0.5, 0.7, 0.9)	0.5
C8	S14	S13	F	VL	(4, 6, 8)	(0.0, 0.1, 0.3)	0.1
C8	S14	S15	A	VL	(5, 7, 9)	(0.0, 0.1, 0.3)	0.1
C8	S14	S16	FA	VC	(6, 8, 10)	(0.7, 0.9, 1.0)	0.1
C8	S14	S17	FA	L	(6, 8, 10)	(0.1, 0.3, 0.5)	0.1
C8	S14	S18	A	L	(5, 7, 9)	(0.1, 0.3, 0.5)	0.1
C8	S14	I	F	C	(4, 6, 8)	(0.5, 0.7, 0.9)	0.5
C9	S14	T1	FA	VC	(6, 8, 10)	(0.7, 0.9, 1.0)	0.12
C9	S14	T2	A	VC	(5, 7, 9)	(0.7, 0.9, 1.0)	0.12
C9	S14	T3	FA	VC	(6, 8, 10)	(0.7, 0.9, 1.0)	0.12
C9	S14	T4	FA	AC	(6, 8, 10)	(0.9, 1.0, 1.0)	0.12
C9	S14	T5	FA	M	(6, 8, 10)	(0.3, 0.5, 0.7)	0.12
C9	S14	IE	FA	VC	(6, 8, 10)	(0.7, 0.9, 1.0)	0.4
C10	S14	T1	VA	AC	(7, 9, 10)	(0.9, 1.0, 1.0)	0.12
C10	S14	T2	FA	AC	(6, 8, 10)	(0.9, 1.0, 1.0)	0.12
C10	S14	T3	VA	C	(7, 9, 10)	(0.5, 0.7, 0.9)	0.12
C10	S14	T4	FA	C	(6, 8, 10)	(0.5, 0.7, 0.9)	0.12

C10	S14	T5	FA	VC	(6, 8, 10)	(0.7, 0.9, 1.0)	0.12
C10	S14	IE	VA	M	(7, 9, 10)	(0.3, 0.5, 0.7)	0.4
C11	S14	T1	FA	M	(6, 8, 10)	(0.3, 0.5, 0.7)	0.2
C11	S14	T2	A	M	(5, 7, 9)	(0.3, 0.5, 0.7)	0.2
C11	S14	T3	FA	AC	(6, 8, 10)	(0.9, 1.0, 1.0)	0.2
C11	S14	T4	VA	AC	(7, 9, 10)	(0.9, 1.0, 1.0)	0.2
C11	S14	T5	FA	AC	(6, 8, 10)	(0.9, 1.0, 1.0)	0.2
C12	S14	T1	VA	AC	(7, 9, 10)	(0.9, 1.0, 1.0)	0.12
C12	S14	T2	A	M	(5, 7, 9)	(0.3, 0.5, 0.7)	0.12
C12	S14	T3	A	M	(5, 7, 9)	(0.3, 0.5, 0.7)	0.12
C12	S14	T4	A	C	(5, 7, 9)	(0.5, 0.7, 0.9)	0.12
C12	S14	T5	A	M	(5, 7, 9)	(0.3, 0.5, 0.7)	0.12
C12	S14	IE	A	AC	(5, 7, 9)	(0.9, 1.0, 1.0)	0.4
C13	S14	I	FI	AC	(3, 5, 7)	(0.9, 1.0, 1.0)	1
C14	S14	I	F	VC	(4, 6, 8)	(0.7, 0.9, 1.0)	1
C15	S14	T1	FA	M	(6, 8, 10)	(0.3, 0.5, 0.7)	0.2
C15	S14	T2	FA	M	(6, 8, 10)	(0.3, 0.5, 0.7)	0.2
C15	S14	T3	VA	C	(7, 9, 10)	(0.5, 0.7, 0.9)	0.2
C15	S14	T4	FA	C	(6, 8, 10)	(0.5, 0.7, 0.9)	0.2
C15	S14	T5	A	AC	(5, 7, 9)	(0.9, 1.0, 1.0)	0.2
C1	S15	S13	FA	M	(6, 8, 10)	(0.3, 0.5, 0.7)	0.1
C1	S15	S14	FA	VL	(6, 8, 10)	(0.0, 0.1, 0.3)	0.1
C1	S15	S16	VA	VL	(7, 9, 10)	(0.0, 0.1, 0.3)	0.1
C1	S15	S17	FA	AC	(6, 8, 10)	(0.9, 1.0, 1.0)	0.1
C1	S15	S18	A	M	(5, 7, 9)	(0.3, 0.5, 0.7)	0.1
C1	S15	I	FA	M	(6, 8, 10)	(0.3, 0.5, 0.7)	0.5
C2	S15	I	VA	C	(7, 9, 10)	(0.5, 0.7, 0.9)	1
C3	S15	S13	VA	M	(7, 9, 10)	(0.3, 0.5, 0.7)	0.1
C3	S15	S14	FA	VL	(6, 8, 10)	(0.0, 0.1, 0.3)	0.1
C3	S15	S16	VA	L	(7, 9, 10)	(0.1, 0.3, 0.5)	0.1
C3	S15	S17	FI	VC	(3, 5, 7)	(0.7, 0.9, 1.0)	0.1
C3	S15	S18	FA	L	(6, 8, 10)	(0.1, 0.3, 0.5)	0.1
C3	S15	I	VA	AC	(7, 9, 10)	(0.9, 1.0, 1.0)	0.5
C4	S15	I	FA	M	(6, 8, 10)	(0.3, 0.5, 0.7)	1
C5	S15	I	VA	VC	(7, 9, 10)	(0.7, 0.9, 1.0)	1
C6	S15	T1	FI	C	(3, 5, 7)	(0.5, 0.7, 0.9)	0.12
C6	S15	T2	A	C	(5, 7, 9)	(0.5, 0.7, 0.9)	0.12
C6	S15	T3	FI	AC	(3, 5, 7)	(0.9, 1.0, 1.0)	0.12
C6	S15	T4	F	VC	(4, 6, 8)	(0.7, 0.9, 1.0)	0.12
C6	S15	T5	F	AC	(4, 6, 8)	(0.9, 1.0, 1.0)	0.12
C6	S15	IE	FA	C	(6, 8, 10)	(0.5, 0.7, 0.9)	0.4
C7	S15	S13	FA	AC	(6, 8, 10)	(0.9, 1.0, 1.0)	0.1
C7	S15	S14	FA	AC	(6, 8, 10)	(0.9, 1.0, 1.0)	0.1
C7	S15	S16	VA	VL	(7, 9, 10)	(0.0, 0.1, 0.3)	0.1
C7	S15	S17	VA	VC	(7, 9, 10)	(0.7, 0.9, 1.0)	0.1
C7	S15	S18	FA	AC	(6, 8, 10)	(0.9, 1.0, 1.0)	0.1
C7	S15	I	FA	VC	(6, 8, 10)	(0.7, 0.9, 1.0)	0.5
C8	S15	S13	F	VL	(4, 6, 8)	(0.0, 0.1, 0.3)	0.1
C8	S15	S14	A	VL	(5, 7, 9)	(0.0, 0.1, 0.3)	0.1
C8	S15	S16	VA	C	(7, 9, 10)	(0.5, 0.7, 0.9)	0.1
C8	S15	S17	FA	L	(6, 8, 10)	(0.1, 0.3, 0.5)	0.1

C8	S15	S18	FA	VL	(6, 8, 10)	(0.0, 0.1, 0.3)	0.1
C8	S15	I	FA	VC	(6, 8, 10)	(0.7, 0.9, 1.0)	0.5
C9	S15	T1	F	AC	(4, 6, 8)	(0.9, 1.0, 1.0)	0.12
C9	S15	T2	F	VC	(4, 6, 8)	(0.7, 0.9, 1.0)	0.12
C9	S15	T3	F	M	(4, 6, 8)	(0.3, 0.5, 0.7)	0.12
C9	S15	T4	A	VC	(5, 7, 9)	(0.7, 0.9, 1.0)	0.12
C9	S15	T5	A	AC	(5, 7, 9)	(0.9, 1.0, 1.0)	0.12
C9	S15	IE	I	AC	(2, 4, 6)	(0.9, 1.0, 1.0)	0.4
C10	S15	T1	A	C	(5, 7, 9)	(0.5, 0.7, 0.9)	0.12
C10	S15	T2	F	VC	(4, 6, 8)	(0.7, 0.9, 1.0)	0.12
C10	S15	T3	F	AC	(4, 6, 8)	(0.9, 1.0, 1.0)	0.12
C10	S15	T4	A	AC	(5, 7, 9)	(0.9, 1.0, 1.0)	0.12
C10	S15	T5	F	C	(4, 6, 8)	(0.5, 0.7, 0.9)	0.12
C10	S15	IE	A	AC	(5, 7, 9)	(0.9, 1.0, 1.0)	0.4
C11	S15	T1	A	M	(5, 7, 9)	(0.3, 0.5, 0.7)	0.2
C11	S15	T2	F	VC	(4, 6, 8)	(0.7, 0.9, 1.0)	0.2
C11	S15	T3	F	C	(4, 6, 8)	(0.5, 0.7, 0.9)	0.2
C11	S15	T4	F	M	(4, 6, 8)	(0.3, 0.5, 0.7)	0.2
C11	S15	T5	FI	M	(3, 5, 7)	(0.3, 0.5, 0.7)	0.2
C12	S15	T1	VA	VC	(7, 9, 10)	(0.7, 0.9, 1.0)	0.12
C12	S15	T2	VA	C	(7, 9, 10)	(0.5, 0.7, 0.9)	0.12
C12	S15	T3	VA	AC	(7, 9, 10)	(0.9, 1.0, 1.0)	0.12
C12	S15	T4	FA	C	(6, 8, 10)	(0.5, 0.7, 0.9)	0.12
C12	S15	T5	VA	AC	(7, 9, 10)	(0.9, 1.0, 1.0)	0.12
C12	S15	IE	VA	VC	(7, 9, 10)	(0.7, 0.9, 1.0)	0.4
C13	S15	I	VA	VC	(7, 9, 10)	(0.7, 0.9, 1.0)	1
C14	S15	I	VA	M	(7, 9, 10)	(0.3, 0.5, 0.7)	1
C15	S15	T1	VA	M	(7, 9, 10)	(0.3, 0.5, 0.7)	0.2
C15	S15	T2	FA	M	(6, 8, 10)	(0.3, 0.5, 0.7)	0.2
C15	S15	T3	FA	VC	(6, 8, 10)	(0.7, 0.9, 1.0)	0.2
C15	S15	T4	FA	AC	(6, 8, 10)	(0.9, 1.0, 1.0)	0.2
C15	S15	T5	VA	VC	(7, 9, 10)	(0.7, 0.9, 1.0)	0.2
C1	S16	S13	I	M	(2, 4, 6)	(0.3, 0.5, 0.7)	0.1
C1	S16	S14	VI	VL	(1, 1, 3)	(0.0, 0.1, 0.3)	0.1
C1	S16	S15	FI	L	(3, 5, 7)	(0.1, 0.3, 0.5)	0.1
C1	S16	S17	VI	VL	(1, 1, 3)	(0.0, 0.1, 0.3)	0.1
C1	S16	S18	FA	L	(6, 8, 10)	(0.1, 0.3, 0.5)	0.1
C1	S16	I	I	AC	(2, 4, 6)	(0.9, 1.0, 1.0)	0.5
C2	S16	I	F	AC	(4, 6, 8)	(0.9, 1.0, 1.0)	1
C3	S16	S13	FI	VC	(3, 5, 7)	(0.7, 0.9, 1.0)	0.1
C3	S16	S14	I	VC	(2, 4, 6)	(0.7, 0.9, 1.0)	0.1
C3	S16	S15	I	VC	(2, 4, 6)	(0.7, 0.9, 1.0)	0.1
C3	S16	S17	FI	C	(3, 5, 7)	(0.5, 0.7, 0.9)	0.1
C3	S16	S18	A	VL	(5, 7, 9)	(0.0, 0.1, 0.3)	0.1
C3	S16	I	FI	M	(3, 5, 7)	(0.3, 0.5, 0.7)	0.5
C4	S16	I	A	M	(5, 7, 9)	(0.3, 0.5, 0.7)	1
C5	S16	I	F	VC	(4, 6, 8)	(0.7, 0.9, 1.0)	1
C6	S16	T1	FA	M	(6, 8, 10)	(0.3, 0.5, 0.7)	0.12
C6	S16	T2	FA	VC	(6, 8, 10)	(0.7, 0.9, 1.0)	0.12
C6	S16	T3	FA	AC	(6, 8, 10)	(0.9, 1.0, 1.0)	0.12
C6	S16	T4	VA	M	(7, 9, 10)	(0.3, 0.5, 0.7)	0.12

C6	S16	T5	FA	VC	(6, 8, 10)	(0.7, 0.9, 1.0)	0.12
C6	S16	IE	FA	AC	(6, 8, 10)	(0.9, 1.0, 1.0)	0.4
C7	S16	S13	F	C	(4, 6, 8)	(0.5, 0.7, 0.9)	0.1
C7	S16	S14	VI	VL	(1, 1, 3)	(0.0, 0.1, 0.3)	0.1
C7	S16	S15	FI	AC	(3, 5, 7)	(0.9, 1.0, 1.0)	0.1
C7	S16	S17	FI	VC	(3, 5, 7)	(0.7, 0.9, 1.0)	0.1
C7	S16	S18	I	L	(2, 4, 6)	(0.1, 0.3, 0.5)	0.1
C7	S16	I	F	C	(4, 6, 8)	(0.5, 0.7, 0.9)	0.5
C8	S16	S13	VI	AC	(1, 1, 3)	(0.9, 1.0, 1.0)	0.1
C8	S16	S14	I	C	(2, 4, 6)	(0.5, 0.7, 0.9)	0.1
C8	S16	S15	FI	L	(3, 5, 7)	(0.1, 0.3, 0.5)	0.1
C8	S16	S17	A	L	(5, 7, 9)	(0.1, 0.3, 0.5)	0.1
C8	S16	S18	I	M	(2, 4, 6)	(0.3, 0.5, 0.7)	0.1
C8	S16	I	FI	VC	(3, 5, 7)	(0.7, 0.9, 1.0)	0.5
C9	S16	T1	FA	VC	(6, 8, 10)	(0.7, 0.9, 1.0)	0.12
C9	S16	T2	FA	AC	(6, 8, 10)	(0.9, 1.0, 1.0)	0.12
C9	S16	T3	A	C	(5, 7, 9)	(0.5, 0.7, 0.9)	0.12
C9	S16	T4	A	M	(5, 7, 9)	(0.3, 0.5, 0.7)	0.12
C9	S16	T5	FA	C	(6, 8, 10)	(0.5, 0.7, 0.9)	0.12
C9	S16	IE	A	AC	(5, 7, 9)	(0.9, 1.0, 1.0)	0.4
C10	S16	T1	VA	VC	(7, 9, 10)	(0.7, 0.9, 1.0)	0.12
C10	S16	T2	VA	AC	(7, 9, 10)	(0.9, 1.0, 1.0)	0.12
C10	S16	T3	FA	VC	(6, 8, 10)	(0.7, 0.9, 1.0)	0.12
C10	S16	T4	FA	AC	(6, 8, 10)	(0.9, 1.0, 1.0)	0.12
C10	S16	T5	VA	M	(7, 9, 10)	(0.3, 0.5, 0.7)	0.12
C10	S16	IE	FA	VC	(6, 8, 10)	(0.7, 0.9, 1.0)	0.4
C11	S16	T1	FA	VC	(6, 8, 10)	(0.7, 0.9, 1.0)	0.2
C11	S16	T2	A	C	(5, 7, 9)	(0.5, 0.7, 0.9)	0.2
C11	S16	T3	FA	AC	(6, 8, 10)	(0.9, 1.0, 1.0)	0.2
C11	S16	T4	VA	C	(7, 9, 10)	(0.5, 0.7, 0.9)	0.2
C11	S16	T5	FA	AC	(6, 8, 10)	(0.9, 1.0, 1.0)	0.2
C12	S16	T1	FI	M	(3, 5, 7)	(0.3, 0.5, 0.7)	0.12
C12	S16	T2	I	C	(2, 4, 6)	(0.5, 0.7, 0.9)	0.12
C12	S16	T3	F	VC	(4, 6, 8)	(0.7, 0.9, 1.0)	0.12
C12	S16	T4	FI	M	(3, 5, 7)	(0.3, 0.5, 0.7)	0.12
C12	S16	T5	F	AC	(4, 6, 8)	(0.9, 1.0, 1.0)	0.12
C12	S16	IE	FI	VC	(3, 5, 7)	(0.7, 0.9, 1.0)	0.4
C13	S16	I	A	AC	(5, 7, 9)	(0.9, 1.0, 1.0)	1
C14	S16	I	A	VC	(5, 7, 9)	(0.7, 0.9, 1.0)	1
C15	S16	T1	FI	AC	(3, 5, 7)	(0.9, 1.0, 1.0)	0.2
C15	S16	T2	F	VC	(4, 6, 8)	(0.7, 0.9, 1.0)	0.2
C15	S16	T3	A	AC	(5, 7, 9)	(0.9, 1.0, 1.0)	0.2
C15	S16	T4	F	C	(4, 6, 8)	(0.5, 0.7, 0.9)	0.2
C15	S16	T5	FI	VC	(3, 5, 7)	(0.7, 0.9, 1.0)	0.2
C1	S17	S13	FA	M	(6, 8, 10)	(0.3, 0.5, 0.7)	0.1
C1	S17	S14	FA	VC	(6, 8, 10)	(0.7, 0.9, 1.0)	0.1
C1	S17	S15	A	AC	(5, 7, 9)	(0.9, 1.0, 1.0)	0.1
C1	S17	S16	A	L	(5, 7, 9)	(0.1, 0.3, 0.5)	0.1
C1	S17	S18	A	C	(5, 7, 9)	(0.5, 0.7, 0.9)	0.1
C1	S17	I	FA	VC	(6, 8, 10)	(0.7, 0.9, 1.0)	0.5
C2	S17	I	F	C	(4, 6, 8)	(0.5, 0.7, 0.9)	1

C3	S17	S13	VA	C	(7, 9, 10)	(0.5, 0.7, 0.9)	0.1
C3	S17	S14	FA	M	(6, 8, 10)	(0.3, 0.5, 0.7)	0.1
C3	S17	S15	FA	M	(6, 8, 10)	(0.3, 0.5, 0.7)	0.1
C3	S17	S16	FA	AC	(6, 8, 10)	(0.9, 1.0, 1.0)	0.1
C3	S17	S18	A	AC	(5, 7, 9)	(0.9, 1.0, 1.0)	0.1
C3	S17	I	FA	AC	(6, 8, 10)	(0.9, 1.0, 1.0)	0.5
C4	S17	I	A	C	(5, 7, 9)	(0.5, 0.7, 0.9)	1
C5	S17	I	A	VC	(5, 7, 9)	(0.7, 0.9, 1.0)	1
C6	S17	T1	FI	AC	(3, 5, 7)	(0.9, 1.0, 1.0)	0.12
C6	S17	T2	A	VC	(5, 7, 9)	(0.7, 0.9, 1.0)	0.12
C6	S17	T3	A	AC	(5, 7, 9)	(0.9, 1.0, 1.0)	0.12
C6	S17	T4	F	VC	(4, 6, 8)	(0.7, 0.9, 1.0)	0.12
C6	S17	T5	F	M	(4, 6, 8)	(0.3, 0.5, 0.7)	0.12
C6	S17	IE	F	C	(4, 6, 8)	(0.5, 0.7, 0.9)	0.4
C7	S17	S13	A	VC	(5, 7, 9)	(0.7, 0.9, 1.0)	0.1
C7	S17	S14	FA	VC	(6, 8, 10)	(0.7, 0.9, 1.0)	0.1
C7	S17	S15	FA	L	(6, 8, 10)	(0.1, 0.3, 0.5)	0.1
C7	S17	S16	A	AC	(5, 7, 9)	(0.9, 1.0, 1.0)	0.1
C7	S17	S18	A	VC	(5, 7, 9)	(0.7, 0.9, 1.0)	0.1
C7	S17	I	FA	M	(6, 8, 10)	(0.3, 0.5, 0.7)	0.5
C8	S17	S13	FA	AC	(6, 8, 10)	(0.9, 1.0, 1.0)	0.1
C8	S17	S14	A	C	(5, 7, 9)	(0.5, 0.7, 0.9)	0.1
C8	S17	S15	VA	AC	(7, 9, 10)	(0.9, 1.0, 1.0)	0.1
C8	S17	S16	FA	L	(6, 8, 10)	(0.1, 0.3, 0.5)	0.1
C8	S17	S18	F	C	(4, 6, 8)	(0.5, 0.7, 0.9)	0.1
C8	S17	I	FA	VC	(6, 8, 10)	(0.7, 0.9, 1.0)	0.5
C9	S17	T1	A	M	(5, 7, 9)	(0.3, 0.5, 0.7)	0.12
C9	S17	T2	F	AC	(4, 6, 8)	(0.9, 1.0, 1.0)	0.12
C9	S17	T3	FI	AC	(3, 5, 7)	(0.9, 1.0, 1.0)	0.12
C9	S17	T4	A	C	(5, 7, 9)	(0.5, 0.7, 0.9)	0.12
C9	S17	T5	F	VC	(4, 6, 8)	(0.7, 0.9, 1.0)	0.12
C9	S17	IE	FI	C	(3, 5, 7)	(0.5, 0.7, 0.9)	0.4
C10	S17	T1	F	VC	(4, 6, 8)	(0.7, 0.9, 1.0)	0.12
C10	S17	T2	A	C	(5, 7, 9)	(0.5, 0.7, 0.9)	0.12
C10	S17	T3	A	AC	(5, 7, 9)	(0.9, 1.0, 1.0)	0.12
C10	S17	T4	A	AC	(5, 7, 9)	(0.9, 1.0, 1.0)	0.12
C10	S17	T5	A	M	(5, 7, 9)	(0.3, 0.5, 0.7)	0.12
C10	S17	IE	F	AC	(4, 6, 8)	(0.9, 1.0, 1.0)	0.4
C11	S17	T1	A	AC	(5, 7, 9)	(0.9, 1.0, 1.0)	0.2
C11	S17	T2	A	AC	(5, 7, 9)	(0.9, 1.0, 1.0)	0.2
C11	S17	T3	A	AC	(5, 7, 9)	(0.9, 1.0, 1.0)	0.2
C11	S17	T4	F	M	(4, 6, 8)	(0.3, 0.5, 0.7)	0.2
C11	S17	T5	A	VC	(5, 7, 9)	(0.7, 0.9, 1.0)	0.2
C12	S17	T1	FA	M	(6, 8, 10)	(0.3, 0.5, 0.7)	0.12
C12	S17	T2	VA	M	(7, 9, 10)	(0.3, 0.5, 0.7)	0.12
C12	S17	T3	VA	C	(7, 9, 10)	(0.5, 0.7, 0.9)	0.12
C12	S17	T4	FA	VC	(6, 8, 10)	(0.7, 0.9, 1.0)	0.12
C12	S17	T5	VA	C	(7, 9, 10)	(0.5, 0.7, 0.9)	0.12
C12	S17	IE	FA	M	(6, 8, 10)	(0.3, 0.5, 0.7)	0.4
C13	S17	I	FA	AC	(6, 8, 10)	(0.9, 1.0, 1.0)	1
C14	S17	I	A	VC	(5, 7, 9)	(0.7, 0.9, 1.0)	1

C15	S17	T1	FA	AC	(6, 8, 10)	(0.9, 1.0, 1.0)	0.2
C15	S17	T2	FA	M	(6, 8, 10)	(0.3, 0.5, 0.7)	0.2
C15	S17	T3	VA	M	(7, 9, 10)	(0.3, 0.5, 0.7)	0.2
C15	S17	T4	VA	M	(7, 9, 10)	(0.3, 0.5, 0.7)	0.2
C15	S17	T5	VA	VC	(7, 9, 10)	(0.7, 0.9, 1.0)	0.2
C1	S18	S13	FA	L	(6, 8, 10)	(0.1, 0.3, 0.5)	0.1
C1	S18	S14	FI	VC	(3, 5, 7)	(0.7, 0.9, 1.0)	0.1
C1	S18	S15	F	AC	(4, 6, 8)	(0.9, 1.0, 1.0)	0.1
C1	S18	S16	FA	VC	(6, 8, 10)	(0.7, 0.9, 1.0)	0.1
C1	S18	S17	A	VC	(5, 7, 9)	(0.7, 0.9, 1.0)	0.1
C1	S18	I	FI	C	(3, 5, 7)	(0.5, 0.7, 0.9)	0.5
C2	S18	I	FA	AC	(6, 8, 10)	(0.9, 1.0, 1.0)	1
C3	S18	S13	I	M	(2, 4, 6)	(0.3, 0.5, 0.7)	0.1
C3	S18	S14	FA	C	(6, 8, 10)	(0.5, 0.7, 0.9)	0.1
C3	S18	S15	FI	C	(3, 5, 7)	(0.5, 0.7, 0.9)	0.1
C3	S18	S16	VA	AC	(7, 9, 10)	(0.9, 1.0, 1.0)	0.1
C3	S18	S17	FA	VC	(6, 8, 10)	(0.7, 0.9, 1.0)	0.1
C3	S18	I	FI	C	(3, 5, 7)	(0.5, 0.7, 0.9)	0.5
C4	S18	I	FA	VC	(6, 8, 10)	(0.7, 0.9, 1.0)	1
C5	S18	I	FA	M	(6, 8, 10)	(0.3, 0.5, 0.7)	1
C6	S18	T1	A	AC	(5, 7, 9)	(0.9, 1.0, 1.0)	0.12
C6	S18	T2	F	M	(4, 6, 8)	(0.3, 0.5, 0.7)	0.12
C6	S18	T3	A	VC	(5, 7, 9)	(0.7, 0.9, 1.0)	0.12
C6	S18	T4	F	AC	(4, 6, 8)	(0.9, 1.0, 1.0)	0.12
C6	S18	T5	F	M	(4, 6, 8)	(0.3, 0.5, 0.7)	0.12
C6	S18	IE	F	M	(4, 6, 8)	(0.3, 0.5, 0.7)	0.4
C7	S18	S13	FA	AC	(6, 8, 10)	(0.9, 1.0, 1.0)	0.1
C7	S18	S14	A	L	(5, 7, 9)	(0.1, 0.3, 0.5)	0.1
C7	S18	S15	A	M	(5, 7, 9)	(0.3, 0.5, 0.7)	0.1
C7	S18	S16	FA	M	(6, 8, 10)	(0.3, 0.5, 0.7)	0.1
C7	S18	S17	I	M	(2, 4, 6)	(0.3, 0.5, 0.7)	0.1
C7	S18	I	A	AC	(5, 7, 9)	(0.9, 1.0, 1.0)	0.5
C8	S18	S13	A	L	(5, 7, 9)	(0.1, 0.3, 0.5)	0.1
C8	S18	S14	FA	VC	(6, 8, 10)	(0.7, 0.9, 1.0)	0.1
C8	S18	S15	I	C	(2, 4, 6)	(0.5, 0.7, 0.9)	0.1
C8	S18	S16	FA	M	(6, 8, 10)	(0.3, 0.5, 0.7)	0.1
C8	S18	S17	F	C	(4, 6, 8)	(0.5, 0.7, 0.9)	0.1
C8	S18	I	A	M	(5, 7, 9)	(0.3, 0.5, 0.7)	0.5
C9	S18	T1	A	AC	(5, 7, 9)	(0.9, 1.0, 1.0)	0.12
C9	S18	T2	F	AC	(4, 6, 8)	(0.9, 1.0, 1.0)	0.12
C9	S18	T3	F	VC	(4, 6, 8)	(0.7, 0.9, 1.0)	0.12
C9	S18	T4	F	M	(4, 6, 8)	(0.3, 0.5, 0.7)	0.12
C9	S18	T5	F	VC	(4, 6, 8)	(0.7, 0.9, 1.0)	0.12
C9	S18	IE	F	M	(4, 6, 8)	(0.3, 0.5, 0.7)	0.4
C10	S18	T1	F	M	(4, 6, 8)	(0.3, 0.5, 0.7)	0.12
C10	S18	T2	A	M	(5, 7, 9)	(0.3, 0.5, 0.7)	0.12
C10	S18	T3	FI	VC	(3, 5, 7)	(0.7, 0.9, 1.0)	0.12
C10	S18	T4	A	M	(5, 7, 9)	(0.3, 0.5, 0.7)	0.12
C10	S18	T5	A	C	(5, 7, 9)	(0.5, 0.7, 0.9)	0.12
C10	S18	IE	F	C	(4, 6, 8)	(0.5, 0.7, 0.9)	0.4
C11	S18	T1	F	C	(4, 6, 8)	(0.5, 0.7, 0.9)	0.2

C11	S18	T2	FI	C	(3, 5, 7)	(0.5, 0.7, 0.9)	0.2
C11	S18	T3	FA	VC	(6, 8, 10)	(0.7, 0.9, 1.0)	0.2
C11	S18	T4	F	AC	(4, 6, 8)	(0.9, 1.0, 1.0)	0.2
C11	S18	T5	F	AC	(4, 6, 8)	(0.9, 1.0, 1.0)	0.2
C12	S18	T1	F	C	(4, 6, 8)	(0.5, 0.7, 0.9)	0.12
C12	S18	T2	A	AC	(5, 7, 9)	(0.9, 1.0, 1.0)	0.12
C12	S18	T3	FA	C	(6, 8, 10)	(0.5, 0.7, 0.9)	0.12
C12	S18	T4	FA	C	(6, 8, 10)	(0.5, 0.7, 0.9)	0.12
C12	S18	T5	FA	AC	(6, 8, 10)	(0.9, 1.0, 1.0)	0.12
C12	S18	IE	FA	AC	(6, 8, 10)	(0.9, 1.0, 1.0)	0.4
C13	S18	I	FA	M	(6, 8, 10)	(0.3, 0.5, 0.7)	1
C14	S18	I	A	C	(5, 7, 9)	(0.5, 0.7, 0.9)	1
C15	S18	T1	A	AC	(5, 7, 9)	(0.9, 1.0, 1.0)	0.2
C15	S18	T2	A	AC	(5, 7, 9)	(0.9, 1.0, 1.0)	0.2
C15	S18	T3	FA	AC	(6, 8, 10)	(0.9, 1.0, 1.0)	0.2
C15	S18	T4	A	M	(5, 7, 9)	(0.3, 0.5, 0.7)	0.2
C15	S18	T5	FA	C	(6, 8, 10)	(0.5, 0.7, 0.9)	0.2
C1	S19	S20	FA	L	(6, 8, 10)	(0.1, 0.3, 0.5)	0.1
C1	S19	S21	F	M	(4, 6, 8)	(0.3, 0.5, 0.7)	0.1
C1	S19	S22	I	AC	(2, 4, 6)	(0.9, 1.0, 1.0)	0.1
C1	S19	S23	VA	VL	(7, 9, 10)	(0.0, 0.1, 0.3)	0.1
C1	S19	S24	VA	VC	(7, 9, 10)	(0.7, 0.9, 1.0)	0.1
C1	S19	I	F	M	(4, 6, 8)	(0.3, 0.5, 0.7)	0.5
C2	S19	I	A	C	(5, 7, 9)	(0.5, 0.7, 0.9)	1
C3	S19	S20	A	M	(5, 7, 9)	(0.3, 0.5, 0.7)	0.1
C3	S19	S21	A	M	(5, 7, 9)	(0.3, 0.5, 0.7)	0.1
C3	S19	S22	VA	L	(7, 9, 10)	(0.1, 0.3, 0.5)	0.1
C3	S19	S23	FA	C	(6, 8, 10)	(0.5, 0.7, 0.9)	0.1
C3	S19	S24	FA	VL	(6, 8, 10)	(0.0, 0.1, 0.3)	0.1
C3	S19	I	A	VC	(5, 7, 9)	(0.7, 0.9, 1.0)	0.5
C4	S19	I	FI	AC	(3, 5, 7)	(0.9, 1.0, 1.0)	1
C5	S19	I	FI	VC	(3, 5, 7)	(0.7, 0.9, 1.0)	1
C6	S19	T1	FA	AC	(6, 8, 10)	(0.9, 1.0, 1.0)	0.12
C6	S19	T2	FA	AC	(6, 8, 10)	(0.9, 1.0, 1.0)	0.12
C6	S19	T3	FA	M	(6, 8, 10)	(0.3, 0.5, 0.7)	0.12
C6	S19	T4	FA	VC	(6, 8, 10)	(0.7, 0.9, 1.0)	0.12
C6	S19	T5	FA	VC	(6, 8, 10)	(0.7, 0.9, 1.0)	0.12
C6	S19	IE	VA	AC	(7, 9, 10)	(0.9, 1.0, 1.0)	0.4
C7	S19	S20	A	VC	(5, 7, 9)	(0.7, 0.9, 1.0)	0.1
C7	S19	S21	FA	L	(6, 8, 10)	(0.1, 0.3, 0.5)	0.1
C7	S19	S22	FI	AC	(3, 5, 7)	(0.9, 1.0, 1.0)	0.1
C7	S19	S23	A	AC	(5, 7, 9)	(0.9, 1.0, 1.0)	0.1
C7	S19	S24	A	M	(5, 7, 9)	(0.3, 0.5, 0.7)	0.1
C7	S19	I	A	M	(5, 7, 9)	(0.3, 0.5, 0.7)	0.5
C8	S19	S20	A	AC	(5, 7, 9)	(0.9, 1.0, 1.0)	0.1
C8	S19	S21	I	L	(2, 4, 6)	(0.1, 0.3, 0.5)	0.1
C8	S19	S22	A	M	(5, 7, 9)	(0.3, 0.5, 0.7)	0.1
C8	S19	S23	FA	VL	(6, 8, 10)	(0.0, 0.1, 0.3)	0.1
C8	S19	S24	FA	L	(6, 8, 10)	(0.1, 0.3, 0.5)	0.1
C8	S19	I	FA	VC	(6, 8, 10)	(0.7, 0.9, 1.0)	0.5
C9	S19	T1	FA	M	(6, 8, 10)	(0.3, 0.5, 0.7)	0.12

C9	S19	T2	VA	C	(7, 9, 10)	(0.5, 0.7, 0.9)	0.12
C9	S19	T3	FA	VC	(6, 8, 10)	(0.7, 0.9, 1.0)	0.12
C9	S19	T4	VA	C	(7, 9, 10)	(0.5, 0.7, 0.9)	0.12
C9	S19	T5	A	AC	(5, 7, 9)	(0.9, 1.0, 1.0)	0.12
C9	S19	IE	FA	AC	(6, 8, 10)	(0.9, 1.0, 1.0)	0.4
C10	S19	T1	VA	C	(7, 9, 10)	(0.5, 0.7, 0.9)	0.12
C10	S19	T2	A	AC	(5, 7, 9)	(0.9, 1.0, 1.0)	0.12
C10	S19	T3	VA	AC	(7, 9, 10)	(0.9, 1.0, 1.0)	0.12
C10	S19	T4	VA	C	(7, 9, 10)	(0.5, 0.7, 0.9)	0.12
C10	S19	T5	VA	AC	(7, 9, 10)	(0.9, 1.0, 1.0)	0.12
C10	S19	IE	FA	C	(6, 8, 10)	(0.5, 0.7, 0.9)	0.4
C11	S19	T1	VA	C	(7, 9, 10)	(0.5, 0.7, 0.9)	0.2
C11	S19	T2	FA	AC	(6, 8, 10)	(0.9, 1.0, 1.0)	0.2
C11	S19	T3	VA	VC	(7, 9, 10)	(0.7, 0.9, 1.0)	0.2
C11	S19	T4	FA	C	(6, 8, 10)	(0.5, 0.7, 0.9)	0.2
C11	S19	T5	VA	C	(7, 9, 10)	(0.5, 0.7, 0.9)	0.2
C12	S19	T1	FA	VC	(6, 8, 10)	(0.7, 0.9, 1.0)	0.12
C12	S19	T2	A	AC	(5, 7, 9)	(0.9, 1.0, 1.0)	0.12
C12	S19	T3	F	M	(4, 6, 8)	(0.3, 0.5, 0.7)	0.12
C12	S19	T4	A	VC	(5, 7, 9)	(0.7, 0.9, 1.0)	0.12
C12	S19	T5	A	M	(5, 7, 9)	(0.3, 0.5, 0.7)	0.12
C12	S19	IE	FA	AC	(6, 8, 10)	(0.9, 1.0, 1.0)	0.4
C13	S19	I	I	C	(2, 4, 6)	(0.5, 0.7, 0.9)	1
C14	S19	I	FI	C	(3, 5, 7)	(0.5, 0.7, 0.9)	1
C15	S19	T1	FA	AC	(6, 8, 10)	(0.9, 1.0, 1.0)	0.2
C15	S19	T2	FA	AC	(6, 8, 10)	(0.9, 1.0, 1.0)	0.2
C15	S19	T3	FA	AC	(6, 8, 10)	(0.9, 1.0, 1.0)	0.2
C15	S19	T4	A	AC	(5, 7, 9)	(0.9, 1.0, 1.0)	0.2
C15	S19	T5	VA	AC	(7, 9, 10)	(0.9, 1.0, 1.0)	0.2
C1	S20	S19	FI	AC	(3, 5, 7)	(0.9, 1.0, 1.0)	0.1
C1	S20	S21	I	C	(2, 4, 6)	(0.5, 0.7, 0.9)	0.1
C1	S20	S22	FI	C	(3, 5, 7)	(0.5, 0.7, 0.9)	0.1
C1	S20	S23	I	L	(2, 4, 6)	(0.1, 0.3, 0.5)	0.1
C1	S20	S24	FI	VL	(3, 5, 7)	(0.0, 0.1, 0.3)	0.1
C1	S20	I	I	M	(2, 4, 6)	(0.3, 0.5, 0.7)	0.5
C2	S20	I	FI	C	(3, 5, 7)	(0.5, 0.7, 0.9)	1
C3	S20	S19	I	M	(2, 4, 6)	(0.3, 0.5, 0.7)	0.1
C3	S20	S21	VI	VL	(1, 1, 3)	(0.0, 0.1, 0.3)	0.1
C3	S20	S22	F	M	(4, 6, 8)	(0.3, 0.5, 0.7)	0.1
C3	S20	S23	VI	L	(1, 1, 3)	(0.1, 0.3, 0.5)	0.1
C3	S20	S24	VI	VC	(1, 1, 3)	(0.7, 0.9, 1.0)	0.1
C3	S20	I	I	VC	(2, 4, 6)	(0.7, 0.9, 1.0)	0.5
C4	S20	I	I	C	(2, 4, 6)	(0.5, 0.7, 0.9)	1
C5	S20	I	FI	AC	(3, 5, 7)	(0.9, 1.0, 1.0)	1
C6	S20	T1	FA	AC	(6, 8, 10)	(0.9, 1.0, 1.0)	0.12
C6	S20	T2	FA	M	(6, 8, 10)	(0.3, 0.5, 0.7)	0.12
C6	S20	T3	FA	M	(6, 8, 10)	(0.3, 0.5, 0.7)	0.12
C6	S20	T4	A	VC	(5, 7, 9)	(0.7, 0.9, 1.0)	0.12
C6	S20	T5	A	VC	(5, 7, 9)	(0.7, 0.9, 1.0)	0.12
C6	S20	IE	A	M	(5, 7, 9)	(0.3, 0.5, 0.7)	0.4
C7	S20	S19	VI	AC	(1, 1, 3)	(0.9, 1.0, 1.0)	0.1

C7	S20	S21	FI	C	(3, 5, 7)	(0.5, 0.7, 0.9)	0.1
C7	S20	S22	VI	VC	(1, 1, 3)	(0.7, 0.9, 1.0)	0.1
C7	S20	S23	A	L	(5, 7, 9)	(0.1, 0.3, 0.5)	0.1
C7	S20	S24	I	C	(2, 4, 6)	(0.5, 0.7, 0.9)	0.1
C7	S20	I	I	M	(2, 4, 6)	(0.3, 0.5, 0.7)	0.5
C8	S20	S19	VI	AC	(1, 1, 3)	(0.9, 1.0, 1.0)	0.1
C8	S20	S21	VI	AC	(1, 1, 3)	(0.9, 1.0, 1.0)	0.1
C8	S20	S22	A	L	(5, 7, 9)	(0.1, 0.3, 0.5)	0.1
C8	S20	S23	FI	M	(3, 5, 7)	(0.3, 0.5, 0.7)	0.1
C8	S20	S24	VI	VL	(1, 1, 3)	(0.0, 0.1, 0.3)	0.1
C8	S20	I	FI	AC	(3, 5, 7)	(0.9, 1.0, 1.0)	0.5
C9	S20	T1	FA	VC	(6, 8, 10)	(0.7, 0.9, 1.0)	0.12
C9	S20	T2	A	VC	(5, 7, 9)	(0.7, 0.9, 1.0)	0.12
C9	S20	T3	A	M	(5, 7, 9)	(0.3, 0.5, 0.7)	0.12
C9	S20	T4	A	AC	(5, 7, 9)	(0.9, 1.0, 1.0)	0.12
C9	S20	T5	FA	M	(6, 8, 10)	(0.3, 0.5, 0.7)	0.12
C9	S20	IE	F	C	(4, 6, 8)	(0.5, 0.7, 0.9)	0.4
C10	S20	T1	FA	C	(6, 8, 10)	(0.5, 0.7, 0.9)	0.12
C10	S20	T2	A	VC	(5, 7, 9)	(0.7, 0.9, 1.0)	0.12
C10	S20	T3	F	AC	(4, 6, 8)	(0.9, 1.0, 1.0)	0.12
C10	S20	T4	VA	C	(7, 9, 10)	(0.5, 0.7, 0.9)	0.12
C10	S20	T5	A	C	(5, 7, 9)	(0.5, 0.7, 0.9)	0.12
C10	S20	IE	FA	C	(6, 8, 10)	(0.5, 0.7, 0.9)	0.4
C11	S20	T1	F	C	(4, 6, 8)	(0.5, 0.7, 0.9)	0.2
C11	S20	T2	A	M	(5, 7, 9)	(0.3, 0.5, 0.7)	0.2
C11	S20	T3	A	VC	(5, 7, 9)	(0.7, 0.9, 1.0)	0.2
C11	S20	T4	F	VC	(4, 6, 8)	(0.7, 0.9, 1.0)	0.2
C11	S20	T5	FA	C	(6, 8, 10)	(0.5, 0.7, 0.9)	0.2
C12	S20	T1	I	VC	(2, 4, 6)	(0.7, 0.9, 1.0)	0.12
C12	S20	T2	FI	VC	(3, 5, 7)	(0.7, 0.9, 1.0)	0.12
C12	S20	T3	FI	VC	(3, 5, 7)	(0.7, 0.9, 1.0)	0.12
C12	S20	T4	I	VC	(2, 4, 6)	(0.7, 0.9, 1.0)	0.12
C12	S20	T5	I	M	(2, 4, 6)	(0.3, 0.5, 0.7)	0.12
C12	S20	IE	I	C	(2, 4, 6)	(0.5, 0.7, 0.9)	0.4
C13	S20	I	FI	AC	(3, 5, 7)	(0.9, 1.0, 1.0)	1
C14	S20	I	FI	M	(3, 5, 7)	(0.3, 0.5, 0.7)	1
C15	S20	T1	I	VC	(2, 4, 6)	(0.7, 0.9, 1.0)	0.2
C15	S20	T2	I	C	(2, 4, 6)	(0.5, 0.7, 0.9)	0.2
C15	S20	T3	I	C	(2, 4, 6)	(0.5, 0.7, 0.9)	0.2
C15	S20	T4	FI	C	(3, 5, 7)	(0.5, 0.7, 0.9)	0.2
C15	S20	T5	I	C	(2, 4, 6)	(0.5, 0.7, 0.9)	0.2
C1	S21	S19	VA	AC	(7, 9, 10)	(0.9, 1.0, 1.0)	0.1
C1	S21	S20	VA	M	(7, 9, 10)	(0.3, 0.5, 0.7)	0.1
C1	S21	S22	FA	L	(6, 8, 10)	(0.1, 0.3, 0.5)	0.1
C1	S21	S23	VA	AC	(7, 9, 10)	(0.9, 1.0, 1.0)	0.1
C1	S21	S24	A	C	(5, 7, 9)	(0.5, 0.7, 0.9)	0.1
C1	S21	I	VA	AC	(7, 9, 10)	(0.9, 1.0, 1.0)	0.5
C2	S21	I	A	C	(5, 7, 9)	(0.5, 0.7, 0.9)	1
C3	S21	S19	FA	VC	(6, 8, 10)	(0.7, 0.9, 1.0)	0.1
C3	S21	S20	VA	AC	(7, 9, 10)	(0.9, 1.0, 1.0)	0.1
C3	S21	S22	F	M	(4, 6, 8)	(0.3, 0.5, 0.7)	0.1

C3	S21	S23	FA	AC	(6, 8, 10)	(0.9, 1.0, 1.0)	0.1
C3	S21	S24	A	C	(5, 7, 9)	(0.5, 0.7, 0.9)	0.1
C3	S21	I	FA	AC	(6, 8, 10)	(0.9, 1.0, 1.0)	0.5
C4	S21	I	VA	AC	(7, 9, 10)	(0.9, 1.0, 1.0)	1
C5	S21	I	VA	M	(7, 9, 10)	(0.3, 0.5, 0.7)	1
C6	S21	T1	F	M	(4, 6, 8)	(0.3, 0.5, 0.7)	0.12
C6	S21	T2	A	AC	(5, 7, 9)	(0.9, 1.0, 1.0)	0.12
C6	S21	T3	FA	VC	(6, 8, 10)	(0.7, 0.9, 1.0)	0.12
C6	S21	T4	FA	VC	(6, 8, 10)	(0.7, 0.9, 1.0)	0.12
C6	S21	T5	VA	AC	(7, 9, 10)	(0.9, 1.0, 1.0)	0.12
C6	S21	IE	VA	AC	(7, 9, 10)	(0.9, 1.0, 1.0)	0.4
C7	S21	S19	FA	M	(6, 8, 10)	(0.3, 0.5, 0.7)	0.1
C7	S21	S20	VA	VC	(7, 9, 10)	(0.7, 0.9, 1.0)	0.1
C7	S21	S22	FA	VL	(6, 8, 10)	(0.0, 0.1, 0.3)	0.1
C7	S21	S23	A	AC	(5, 7, 9)	(0.9, 1.0, 1.0)	0.1
C7	S21	S24	FA	VL	(6, 8, 10)	(0.0, 0.1, 0.3)	0.1
C7	S21	I	A	AC	(5, 7, 9)	(0.9, 1.0, 1.0)	0.5
C8	S21	S19	VA	AC	(7, 9, 10)	(0.9, 1.0, 1.0)	0.1
C8	S21	S20	VA	AC	(7, 9, 10)	(0.9, 1.0, 1.0)	0.1
C8	S21	S22	A	AC	(5, 7, 9)	(0.9, 1.0, 1.0)	0.1
C8	S21	S23	FA	M	(6, 8, 10)	(0.3, 0.5, 0.7)	0.1
C8	S21	S24	FA	VL	(6, 8, 10)	(0.0, 0.1, 0.3)	0.1
C8	S21	I	A	AC	(5, 7, 9)	(0.9, 1.0, 1.0)	0.5
C9	S21	T1	F	C	(4, 6, 8)	(0.5, 0.7, 0.9)	0.12
C9	S21	T2	FA	AC	(6, 8, 10)	(0.9, 1.0, 1.0)	0.12
C9	S21	T3	A	C	(5, 7, 9)	(0.5, 0.7, 0.9)	0.12
C9	S21	T4	A	AC	(5, 7, 9)	(0.9, 1.0, 1.0)	0.12
C9	S21	T5	FA	VC	(6, 8, 10)	(0.7, 0.9, 1.0)	0.12
C9	S21	IE	VA	VC	(7, 9, 10)	(0.7, 0.9, 1.0)	0.4
C10	S21	T1	FA	VC	(6, 8, 10)	(0.7, 0.9, 1.0)	0.12
C10	S21	T2	FA	VC	(6, 8, 10)	(0.7, 0.9, 1.0)	0.12
C10	S21	T3	FA	C	(6, 8, 10)	(0.5, 0.7, 0.9)	0.12
C10	S21	T4	FA	AC	(6, 8, 10)	(0.9, 1.0, 1.0)	0.12
C10	S21	T5	FA	VC	(6, 8, 10)	(0.7, 0.9, 1.0)	0.12
C10	S21	IE	FA	M	(6, 8, 10)	(0.3, 0.5, 0.7)	0.4
C11	S21	T1	FA	M	(6, 8, 10)	(0.3, 0.5, 0.7)	0.2
C11	S21	T2	A	M	(5, 7, 9)	(0.3, 0.5, 0.7)	0.2
C11	S21	T3	VA	AC	(7, 9, 10)	(0.9, 1.0, 1.0)	0.2
C11	S21	T4	VA	M	(7, 9, 10)	(0.3, 0.5, 0.7)	0.2
C11	S21	T5	A	M	(5, 7, 9)	(0.3, 0.5, 0.7)	0.2
C12	S21	T1	VA	C	(7, 9, 10)	(0.5, 0.7, 0.9)	0.12
C12	S21	T2	VA	AC	(7, 9, 10)	(0.9, 1.0, 1.0)	0.12
C12	S21	T3	FA	AC	(6, 8, 10)	(0.9, 1.0, 1.0)	0.12
C12	S21	T4	VA	M	(7, 9, 10)	(0.3, 0.5, 0.7)	0.12
C12	S21	T5	VA	AC	(7, 9, 10)	(0.9, 1.0, 1.0)	0.12
C12	S21	IE	FA	C	(6, 8, 10)	(0.5, 0.7, 0.9)	0.4
C13	S21	I	VA	C	(7, 9, 10)	(0.5, 0.7, 0.9)	1
C14	S21	I	FA	VC	(6, 8, 10)	(0.7, 0.9, 1.0)	1
C15	S21	T1	VA	C	(7, 9, 10)	(0.5, 0.7, 0.9)	0.2
C15	S21	T2	FA	VC	(6, 8, 10)	(0.7, 0.9, 1.0)	0.2
C15	S21	T3	FA	M	(6, 8, 10)	(0.3, 0.5, 0.7)	0.2

C15	S21	T4	VA	VC	(7, 9, 10)	(0.7, 0.9, 1.0)	0.2
C15	S21	T5	VA	VC	(7, 9, 10)	(0.7, 0.9, 1.0)	0.2
C1	S22	S19	I	VL	(2, 4, 6)	(0.0, 0.1, 0.3)	0.1
C1	S22	S20	I	M	(2, 4, 6)	(0.3, 0.5, 0.7)	0.1
C1	S22	S21	I	AC	(2, 4, 6)	(0.9, 1.0, 1.0)	0.1
C1	S22	S23	F	AC	(4, 6, 8)	(0.9, 1.0, 1.0)	0.1
C1	S22	S24	F	L	(4, 6, 8)	(0.1, 0.3, 0.5)	0.1
C1	S22	I	FI	M	(3, 5, 7)	(0.3, 0.5, 0.7)	0.5
C2	S22	I	F	AC	(4, 6, 8)	(0.9, 1.0, 1.0)	1
C3	S22	S19	FI	AC	(3, 5, 7)	(0.9, 1.0, 1.0)	0.1
C3	S22	S20	FI	M	(3, 5, 7)	(0.3, 0.5, 0.7)	0.1
C3	S22	S21	FA	AC	(6, 8, 10)	(0.9, 1.0, 1.0)	0.1
C3	S22	S23	FI	AC	(3, 5, 7)	(0.9, 1.0, 1.0)	0.1
C3	S22	S24	FA	L	(6, 8, 10)	(0.1, 0.3, 0.5)	0.1
C3	S22	I	I	AC	(2, 4, 6)	(0.9, 1.0, 1.0)	0.5
C4	S22	I	F	M	(4, 6, 8)	(0.3, 0.5, 0.7)	1
C5	S22	I	F	M	(4, 6, 8)	(0.3, 0.5, 0.7)	1
C6	S22	T1	VA	M	(7, 9, 10)	(0.3, 0.5, 0.7)	0.12
C6	S22	T2	VA	C	(7, 9, 10)	(0.5, 0.7, 0.9)	0.12
C6	S22	T3	FA	AC	(6, 8, 10)	(0.9, 1.0, 1.0)	0.12
C6	S22	T4	FA	M	(6, 8, 10)	(0.3, 0.5, 0.7)	0.12
C6	S22	T5	FA	C	(6, 8, 10)	(0.5, 0.7, 0.9)	0.12
C6	S22	IE	A	C	(5, 7, 9)	(0.5, 0.7, 0.9)	0.4
C7	S22	S19	A	VC	(5, 7, 9)	(0.7, 0.9, 1.0)	0.1
C7	S22	S20	F	AC	(4, 6, 8)	(0.9, 1.0, 1.0)	0.1
C7	S22	S21	F	C	(4, 6, 8)	(0.5, 0.7, 0.9)	0.1
C7	S22	S23	I	VL	(2, 4, 6)	(0.0, 0.1, 0.3)	0.1
C7	S22	S24	FA	VL	(6, 8, 10)	(0.0, 0.1, 0.3)	0.1
C7	S22	I	FI	VC	(3, 5, 7)	(0.7, 0.9, 1.0)	0.5
C8	S22	S19	F	VL	(4, 6, 8)	(0.0, 0.1, 0.3)	0.1
C8	S22	S20	VI	C	(1, 1, 3)	(0.5, 0.7, 0.9)	0.1
C8	S22	S21	FI	AC	(3, 5, 7)	(0.9, 1.0, 1.0)	0.1
C8	S22	S23	F	L	(4, 6, 8)	(0.1, 0.3, 0.5)	0.1
C8	S22	S24	F	C	(4, 6, 8)	(0.5, 0.7, 0.9)	0.1
C8	S22	I	FI	M	(3, 5, 7)	(0.3, 0.5, 0.7)	0.5
C9	S22	T1	FA	C	(6, 8, 10)	(0.5, 0.7, 0.9)	0.12
C9	S22	T2	A	AC	(5, 7, 9)	(0.9, 1.0, 1.0)	0.12
C9	S22	T3	FA	M	(6, 8, 10)	(0.3, 0.5, 0.7)	0.12
C9	S22	T4	FA	M	(6, 8, 10)	(0.3, 0.5, 0.7)	0.12
C9	S22	T5	FA	M	(6, 8, 10)	(0.3, 0.5, 0.7)	0.12
C9	S22	IE	FA	VC	(6, 8, 10)	(0.7, 0.9, 1.0)	0.4
C10	S22	T1	FA	VC	(6, 8, 10)	(0.7, 0.9, 1.0)	0.12
C10	S22	T2	FA	VC	(6, 8, 10)	(0.7, 0.9, 1.0)	0.12
C10	S22	T3	A	C	(5, 7, 9)	(0.5, 0.7, 0.9)	0.12
C10	S22	T4	VA	AC	(7, 9, 10)	(0.9, 1.0, 1.0)	0.12
C10	S22	T5	VA	C	(7, 9, 10)	(0.5, 0.7, 0.9)	0.12
C10	S22	IE	VA	C	(7, 9, 10)	(0.5, 0.7, 0.9)	0.4
C11	S22	T1	FA	M	(6, 8, 10)	(0.3, 0.5, 0.7)	0.2
C11	S22	T2	A	M	(5, 7, 9)	(0.3, 0.5, 0.7)	0.2
C11	S22	T3	FA	C	(6, 8, 10)	(0.5, 0.7, 0.9)	0.2
C11	S22	T4	FA	AC	(6, 8, 10)	(0.9, 1.0, 1.0)	0.2

C11	S22	T5	FA	VC	(6, 8, 10)	(0.7, 0.9, 1.0)	0.2
C12	S22	T1	F	C	(4, 6, 8)	(0.5, 0.7, 0.9)	0.12
C12	S22	T2	F	C	(4, 6, 8)	(0.5, 0.7, 0.9)	0.12
C12	S22	T3	F	AC	(4, 6, 8)	(0.9, 1.0, 1.0)	0.12
C12	S22	T4	F	AC	(4, 6, 8)	(0.9, 1.0, 1.0)	0.12
C12	S22	T5	F	C	(4, 6, 8)	(0.5, 0.7, 0.9)	0.12
C12	S22	IE	A	M	(5, 7, 9)	(0.3, 0.5, 0.7)	0.4
C13	S22	I	A	M	(5, 7, 9)	(0.3, 0.5, 0.7)	1
C14	S22	I	F	C	(4, 6, 8)	(0.5, 0.7, 0.9)	1
C15	S22	T1	FI	M	(3, 5, 7)	(0.3, 0.5, 0.7)	0.2
C15	S22	T2	F	AC	(4, 6, 8)	(0.9, 1.0, 1.0)	0.2
C15	S22	T3	A	VC	(5, 7, 9)	(0.7, 0.9, 1.0)	0.2
C15	S22	T4	F	AC	(4, 6, 8)	(0.9, 1.0, 1.0)	0.2
C15	S22	T5	F	VC	(4, 6, 8)	(0.7, 0.9, 1.0)	0.2
C1	S23	S19	F	L	(4, 6, 8)	(0.1, 0.3, 0.5)	0.1
C1	S23	S20	FA	L	(6, 8, 10)	(0.1, 0.3, 0.5)	0.1
C1	S23	S21	A	M	(5, 7, 9)	(0.3, 0.5, 0.7)	0.1
C1	S23	S22	FA	AC	(6, 8, 10)	(0.9, 1.0, 1.0)	0.1
C1	S23	S24	VA	VL	(7, 9, 10)	(0.0, 0.1, 0.3)	0.1
C1	S23	I	FA	C	(6, 8, 10)	(0.5, 0.7, 0.9)	0.5
C2	S23	I	F	VC	(4, 6, 8)	(0.7, 0.9, 1.0)	1
C3	S23	S19	FI	AC	(3, 5, 7)	(0.9, 1.0, 1.0)	0.1
C3	S23	S20	A	VL	(5, 7, 9)	(0.0, 0.1, 0.3)	0.1
C3	S23	S21	FI	AC	(3, 5, 7)	(0.9, 1.0, 1.0)	0.1
C3	S23	S22	FA	M	(6, 8, 10)	(0.3, 0.5, 0.7)	0.1
C3	S23	S24	A	L	(5, 7, 9)	(0.1, 0.3, 0.5)	0.1
C3	S23	I	A	C	(5, 7, 9)	(0.5, 0.7, 0.9)	0.5
C4	S23	I	A	AC	(5, 7, 9)	(0.9, 1.0, 1.0)	1
C5	S23	I	VA	VC	(7, 9, 10)	(0.7, 0.9, 1.0)	1
C6	S23	T1	VA	M	(7, 9, 10)	(0.3, 0.5, 0.7)	0.12
C6	S23	T2	A	C	(5, 7, 9)	(0.5, 0.7, 0.9)	0.12
C6	S23	T3	FA	AC	(6, 8, 10)	(0.9, 1.0, 1.0)	0.12
C6	S23	T4	FA	AC	(6, 8, 10)	(0.9, 1.0, 1.0)	0.12
C6	S23	T5	A	M	(5, 7, 9)	(0.3, 0.5, 0.7)	0.12
C6	S23	IE	A	C	(5, 7, 9)	(0.5, 0.7, 0.9)	0.4
C7	S23	S19	F	C	(4, 6, 8)	(0.5, 0.7, 0.9)	0.1
C7	S23	S20	F	AC	(4, 6, 8)	(0.9, 1.0, 1.0)	0.1
C7	S23	S21	FA	VC	(6, 8, 10)	(0.7, 0.9, 1.0)	0.1
C7	S23	S22	A	VL	(5, 7, 9)	(0.0, 0.1, 0.3)	0.1
C7	S23	S24	I	VC	(2, 4, 6)	(0.7, 0.9, 1.0)	0.1
C7	S23	I	FA	M	(6, 8, 10)	(0.3, 0.5, 0.7)	0.5
C8	S23	S19	A	M	(5, 7, 9)	(0.3, 0.5, 0.7)	0.1
C8	S23	S20	VA	M	(7, 9, 10)	(0.3, 0.5, 0.7)	0.1
C8	S23	S21	FA	L	(6, 8, 10)	(0.1, 0.3, 0.5)	0.1
C8	S23	S22	FI	C	(3, 5, 7)	(0.5, 0.7, 0.9)	0.1
C8	S23	S24	I	VC	(2, 4, 6)	(0.7, 0.9, 1.0)	0.1
C8	S23	I	A	AC	(5, 7, 9)	(0.9, 1.0, 1.0)	0.5
C9	S23	T1	A	VC	(5, 7, 9)	(0.7, 0.9, 1.0)	0.12
C9	S23	T2	FA	C	(6, 8, 10)	(0.5, 0.7, 0.9)	0.12
C9	S23	T3	A	AC	(5, 7, 9)	(0.9, 1.0, 1.0)	0.12
C9	S23	T4	FA	M	(6, 8, 10)	(0.3, 0.5, 0.7)	0.12

C9	S23	T5	F	M	(4, 6, 8)	(0.3, 0.5, 0.7)	0.12
C9	S23	IE	FA	M	(6, 8, 10)	(0.3, 0.5, 0.7)	0.4
C10	S23	T1	FA	M	(6, 8, 10)	(0.3, 0.5, 0.7)	0.12
C10	S23	T2	F	VC	(4, 6, 8)	(0.7, 0.9, 1.0)	0.12
C10	S23	T3	VA	C	(7, 9, 10)	(0.5, 0.7, 0.9)	0.12
C10	S23	T4	FA	M	(6, 8, 10)	(0.3, 0.5, 0.7)	0.12
C10	S23	T5	FA	M	(6, 8, 10)	(0.3, 0.5, 0.7)	0.12
C10	S23	IE	A	AC	(5, 7, 9)	(0.9, 1.0, 1.0)	0.4
C11	S23	T1	FA	VC	(6, 8, 10)	(0.7, 0.9, 1.0)	0.2
C11	S23	T2	A	AC	(5, 7, 9)	(0.9, 1.0, 1.0)	0.2
C11	S23	T3	FA	AC	(6, 8, 10)	(0.9, 1.0, 1.0)	0.2
C11	S23	T4	FA	AC	(6, 8, 10)	(0.9, 1.0, 1.0)	0.2
C11	S23	T5	A	M	(5, 7, 9)	(0.3, 0.5, 0.7)	0.2
C12	S23	T1	VA	C	(7, 9, 10)	(0.5, 0.7, 0.9)	0.12
C12	S23	T2	VA	VC	(7, 9, 10)	(0.7, 0.9, 1.0)	0.12
C12	S23	T3	VA	M	(7, 9, 10)	(0.3, 0.5, 0.7)	0.12
C12	S23	T4	FA	VC	(6, 8, 10)	(0.7, 0.9, 1.0)	0.12
C12	S23	T5	VA	M	(7, 9, 10)	(0.3, 0.5, 0.7)	0.12
C12	S23	IE	FA	C	(6, 8, 10)	(0.5, 0.7, 0.9)	0.4
C13	S23	I	FA	C	(6, 8, 10)	(0.5, 0.7, 0.9)	1
C14	S23	I	A	VC	(5, 7, 9)	(0.7, 0.9, 1.0)	1
C15	S23	T1	FA	AC	(6, 8, 10)	(0.9, 1.0, 1.0)	0.2
C15	S23	T2	VA	M	(7, 9, 10)	(0.3, 0.5, 0.7)	0.2
C15	S23	T3	VA	VC	(7, 9, 10)	(0.7, 0.9, 1.0)	0.2
C15	S23	T4	FA	AC	(6, 8, 10)	(0.9, 1.0, 1.0)	0.2
C15	S23	T5	VA	C	(7, 9, 10)	(0.5, 0.7, 0.9)	0.2
C1	S24	S19	FA	VL	(6, 8, 10)	(0.0, 0.1, 0.3)	0.1
C1	S24	S20	FI	L	(3, 5, 7)	(0.1, 0.3, 0.5)	0.1
C1	S24	S21	FA	VL	(6, 8, 10)	(0.0, 0.1, 0.3)	0.1
C1	S24	S22	I	C	(2, 4, 6)	(0.5, 0.7, 0.9)	0.1
C1	S24	S23	FI	VL	(3, 5, 7)	(0.0, 0.1, 0.3)	0.1
C1	S24	I	F	AC	(4, 6, 8)	(0.9, 1.0, 1.0)	0.5
C2	S24	I	FI	M	(3, 5, 7)	(0.3, 0.5, 0.7)	1
C3	S24	S19	F	M	(4, 6, 8)	(0.3, 0.5, 0.7)	0.1
C3	S24	S20	F	AC	(4, 6, 8)	(0.9, 1.0, 1.0)	0.1
C3	S24	S21	FI	AC	(3, 5, 7)	(0.9, 1.0, 1.0)	0.1
C3	S24	S22	A	AC	(5, 7, 9)	(0.9, 1.0, 1.0)	0.1
C3	S24	S23	FI	AC	(3, 5, 7)	(0.9, 1.0, 1.0)	0.1
C3	S24	I	F	C	(4, 6, 8)	(0.5, 0.7, 0.9)	0.5
C4	S24	I	F	M	(4, 6, 8)	(0.3, 0.5, 0.7)	1
C5	S24	I	A	M	(5, 7, 9)	(0.3, 0.5, 0.7)	1
C6	S24	T1	VA	C	(7, 9, 10)	(0.5, 0.7, 0.9)	0.12
C6	S24	T2	FA	M	(6, 8, 10)	(0.3, 0.5, 0.7)	0.12
C6	S24	T3	VA	C	(7, 9, 10)	(0.5, 0.7, 0.9)	0.12
C6	S24	T4	FA	AC	(6, 8, 10)	(0.9, 1.0, 1.0)	0.12
C6	S24	T5	FA	VC	(6, 8, 10)	(0.7, 0.9, 1.0)	0.12
C6	S24	IE	VA	VC	(7, 9, 10)	(0.7, 0.9, 1.0)	0.4
C7	S24	S19	FI	L	(3, 5, 7)	(0.1, 0.3, 0.5)	0.1
C7	S24	S20	FA	M	(6, 8, 10)	(0.3, 0.5, 0.7)	0.1
C7	S24	S21	FA	L	(6, 8, 10)	(0.1, 0.3, 0.5)	0.1
C7	S24	S22	I	AC	(2, 4, 6)	(0.9, 1.0, 1.0)	0.1

C7	S24	S23	FI	M	(3, 5, 7)	(0.3, 0.5, 0.7)	0.1
C7	S24	I	FI	M	(3, 5, 7)	(0.3, 0.5, 0.7)	0.5
C8	S24	S19	FI	VL	(3, 5, 7)	(0.0, 0.1, 0.3)	0.1
C8	S24	S20	A	M	(5, 7, 9)	(0.3, 0.5, 0.7)	0.1
C8	S24	S21	F	C	(4, 6, 8)	(0.5, 0.7, 0.9)	0.1
C8	S24	S22	FI	M	(3, 5, 7)	(0.3, 0.5, 0.7)	0.1
C8	S24	S23	FA	VC	(6, 8, 10)	(0.7, 0.9, 1.0)	0.1
C8	S24	I	I	M	(2, 4, 6)	(0.3, 0.5, 0.7)	0.5
C9	S24	T1	VA	VC	(7, 9, 10)	(0.7, 0.9, 1.0)	0.12
C9	S24	T2	FA	M	(6, 8, 10)	(0.3, 0.5, 0.7)	0.12
C9	S24	T3	FA	AC	(6, 8, 10)	(0.9, 1.0, 1.0)	0.12
C9	S24	T4	FA	AC	(6, 8, 10)	(0.9, 1.0, 1.0)	0.12
C9	S24	T5	VA	VC	(7, 9, 10)	(0.7, 0.9, 1.0)	0.12
C9	S24	IE	FA	M	(6, 8, 10)	(0.3, 0.5, 0.7)	0.4
C10	S24	T1	VA	AC	(7, 9, 10)	(0.9, 1.0, 1.0)	0.12
C10	S24	T2	VA	C	(7, 9, 10)	(0.5, 0.7, 0.9)	0.12
C10	S24	T3	FA	VC	(6, 8, 10)	(0.7, 0.9, 1.0)	0.12
C10	S24	T4	FA	C	(6, 8, 10)	(0.5, 0.7, 0.9)	0.12
C10	S24	T5	VA	VC	(7, 9, 10)	(0.7, 0.9, 1.0)	0.12
C10	S24	IE	FA	M	(6, 8, 10)	(0.3, 0.5, 0.7)	0.4
C11	S24	T1	FA	M	(6, 8, 10)	(0.3, 0.5, 0.7)	0.2
C11	S24	T2	VA	C	(7, 9, 10)	(0.5, 0.7, 0.9)	0.2
C11	S24	T3	VA	M	(7, 9, 10)	(0.3, 0.5, 0.7)	0.2
C11	S24	T4	VA	M	(7, 9, 10)	(0.3, 0.5, 0.7)	0.2
C11	S24	T5	VA	M	(7, 9, 10)	(0.3, 0.5, 0.7)	0.2
C12	S24	T1	F	C	(4, 6, 8)	(0.5, 0.7, 0.9)	0.12
C12	S24	T2	F	M	(4, 6, 8)	(0.3, 0.5, 0.7)	0.12
C12	S24	T3	FA	AC	(6, 8, 10)	(0.9, 1.0, 1.0)	0.12
C12	S24	T4	F	VC	(4, 6, 8)	(0.7, 0.9, 1.0)	0.12
C12	S24	T5	F	AC	(4, 6, 8)	(0.9, 1.0, 1.0)	0.12
C12	S24	IE	FI	C	(3, 5, 7)	(0.5, 0.7, 0.9)	0.4
C13	S24	I	F	C	(4, 6, 8)	(0.5, 0.7, 0.9)	1
C14	S24	I	F	M	(4, 6, 8)	(0.3, 0.5, 0.7)	1
C15	S24	T1	A	AC	(5, 7, 9)	(0.9, 1.0, 1.0)	0.2
C15	S24	T2	A	C	(5, 7, 9)	(0.5, 0.7, 0.9)	0.2
C15	S24	T3	FA	VC	(6, 8, 10)	(0.7, 0.9, 1.0)	0.2
C15	S24	T4	A	C	(5, 7, 9)	(0.5, 0.7, 0.9)	0.2
C15	S24	T5	VA	AC	(7, 9, 10)	(0.9, 1.0, 1.0)	0.2
