# Supplementary material for: A novel Z-number based multi-stage assessment framework for problem-based learning in practical courses
Source: PLoS One. 2026 May 18;21(5):e0349114. doi: 10.1371/journal.pone.0349114 (PMC13183222; doi:10.1371/journal.pone.0349114)
Supplement: S2 File — Anonymized linguistic evaluation tables for criteria importance provided by instructors (T1–T5), together with the corresponding TFN mappings for restriction and reliability. (PDF) [file pone.0349114.s002.pdf]

Linguistic evaluations of criteria importance（instructor T1）

Criterion	Restriction	Reliability	Restriction (TFN)	Reliability (TFN)
C1	VA	VC	(7,9,10)	(0.7,0.9,1.0)
C2	VA	VC	(7,9,10)	(0.7,0.9,1.0)
C3	VA	VC	(7,9,10)	(0.7,0.9,1.0)
C4	A	VC	(5,7,9)	(0.7,0.9,1.0)
C5	VA	AC	(7,9,10)	(0.9,1.0,1.0)
C6	FA	VC	(6,8,10)	(0.7,0.9,1.0)
C7	FA	VC	(6,8,10)	(0.7,0.9,1.0)
C8	A	VC	(5,7,9)	(0.7,0.9,1.0)
C9	A	M	(5,7,9)	(0.3,0.5,0.7)
C10	VA	VC	(7,9,10)	(0.7,0.9,1.0)
C11	VA	AC	(7,9,10)	(0.9,1.0,1.0)
C12	FA	VC	(6,8,10)	(0.7,0.9,1.0)
C13	FA	VC	(6,8,10)	(0.7,0.9,1.0)
C14	VA	AC	(7,9,10)	(0.9,1.0,1.0)
C15	FA	VC	(6,8,10)	(0.7,0.9,1.0)

Linguistic evaluations of criteria importance（instructor T2）

Criterion	Restriction	Reliability	Restriction (TFN)	Reliability (TFN)
C1	FA	C	(6,8,10)	(0.5,0.7,0.9)
C2	A	VC	(5,7,9)	(0.7,0.9,1.0)
C3	FA	C	(6,8,10)	(0.5,0.7,0.9)
C4	FA	VC	(6,8,10)	(0.7,0.9,1.0)
C5	VA	VC	(7,9,10)	(0.7,0.9,1.0)
C6	FA	C	(6,8,10)	(0.5,0.7,0.9)
C7	FA	VC	(6,8,10)	(0.7,0.9,1.0)
C8	FA	C	(6,8,10)	(0.5,0.7,0.9)
C9	VA	VC	(7,9,10)	(0.7,0.9,1.0)
C10	VA	VC	(7,9,10)	(0.7,0.9,1.0)
C11	FA	C	(6,8,10)	(0.5,0.7,0.9)
C12	FA	VC	(6,8,10)	(0.7,0.9,1.0)
C13	VA	VC	(7,9,10)	(0.7,0.9,1.0)
C14	VA	VC	(7,9,10)	(0.7,0.9,1.0)
C15	VA	VC	(7,9,10)	(0.7,0.9,1.0)

Linguistic evaluations of criteria importance（instructor T3）

Criterion	Restriction	Reliability	Restriction (TFN)	Reliability (TFN)
C1	VA	VC	(7,9,10)	(0.7,0.9,1.0)
C2	FA	VC	(6,8,10)	(0.7,0.9,1.0)
C3	A	C	(5,7,9)	(0.5,0.7,0.9)
C4	F	M	(4,6,8)	(0.3,0.5,0.7)
C5	VA	VC	(7,9,10)	(0.7,0.9,1.0)
C6	A	C	(5,7,9)	(0.5,0.7,0.9)
C7	FA	VC	(6,8,10)	(0.7,0.9,1.0)
C8	F	M	(4,6,8)	(0.3,0.5,0.7)
C9	VA	VC	(7,9,10)	(0.7,0.9,1.0)
C10	VA	VC	(7,9,10)	(0.7,0.9,1.0)
C11	A	C	(5,7,9)	(0.5,0.7,0.9)
C12	FA	VC	(6,8,10)	(0.7,0.9,1.0)
C13	A	C	(5,7,9)	(0.5,0.7,0.9)
C14	VA	VC	(7,9,10)	(0.7,0.9,1.0)
C15	FA	VC	(6,8,10)	(0.7,0.9,1.0)

Linguistic evaluations of criteria importance（instructor T4）

Criterion	Restriction	Reliability	Restriction (TFN)	Reliability (TFN)
C1	FA	VC	(6,8,10)	(0.7,0.9,1.0)
C2	A	VC	(5,7,9)	(0.7,0.9,1.0)
C3	FA	VC	(6,8,10)	(0.7,0.9,1.0)
C4	A	C	(5,7,9)	(0.5,0.7,0.9)
C5	VA	AC	(7,9,10)	(0.9,1.0,1.0)
C6	F	VC	(4,6,8)	(0.7,0.9,1.0)
C7	A	VC	(5,7,9)	(0.7,0.9,1.0)
C8	FA	C	(6,8,10)	(0.5,0.7,0.9)
C9	VA	C	(7,9,10)	(0.5,0.7,0.9)
C10	FA	VC	(6,8,10)	(0.7,0.9,1.0)
C11	VA	AC	(7,9,10)	(0.9,1.0,1.0)
C12	A	VC	(5,7,9)	(0.7,0.9,1.0)
C13	VA	AC	(7,9,10)	(0.9,1.0,1.0)
C14	VA	AC	(7,9,10)	(0.9,1.0,1.0)
C15	FA	VC	(6,8,10)	(0.7,0.9,1.0)

Linguistic evaluations of criteria importance（instructor T5）

Criterion	Restriction	Reliability	Restriction (TFN)	Reliability (TFN)
C1	VA	VC	(7,9,10)	(0.7,0.9,1.0)
C2	A	C	(5,7,9)	(0.5,0.7,0.9)
C3	A	C	(5,7,9)	(0.5,0.7,0.9)
C4	F	C	(4,6,8)	(0.5,0.7,0.9)
C5	VA	VC	(7,9,10)	(0.7,0.9,1.0)
C6	A	C	(5,7,9)	(0.5,0.7,0.9)
C7	FA	VC	(6,8,10)	(0.7,0.9,1.0)
C8	F	C	(4,6,8)	(0.5,0.7,0.9)
C9	VA	VC	(7,9,10)	(0.7,0.9,1.0)
C10	A	VC	(5,7,9)	(0.7,0.9,1.0)
C11	FA	C	(6,8,10)	(0.5,0.7,0.9)
C12	F	C	(4,6,8)	(0.5,0.7,0.9)
C13	VA	AC	(7,9,10)	(0.9,1.0,1.0)
C14	VA	VC	(7,9,10)	(0.7,0.9,1.0)
C15	A	C	(5,7,9)	(0.5,0.7,0.9)
